# Supplementary material for: Standard echocardiography versus handheld echocardiography for the detection of subclinical rheumatic heart disease: a systematic review and meta-analysis of diagnostic accuracy
Source: BMJ Open. 2020 Oct 29;10(10):e038449. doi: 10.1136/bmjopen-2020-038449 (PMC7597508; doi:10.1136/bmjopen-2020-038449)
Supplement: Supplementary data [file bmjopen-2020-038449supp001.pdf]

Supplementary file 1. Search strategy.

Database	Search Terms	Limits
**PubMed**	(((((((((((((((Hand-held) OR handheld) OR hand held) OR hand-carried) OR hand carried) OR HAND) OR HCU) OR HHCU) OR pocket size) OR pocket sized) OR portable) OR miniaturization) OR miniaturized) OR focused) OR focus)) AND (((("Echocardiography"[Mesh]) OR echocardiography) OR echocardiographic) OR cardiac ultrasound)) AND ((("Rheumatic Heart Disease"[Mesh]) OR rheumatic heart disease) OR RHD)<br>[MeSH terms were exploded during the search]	Limited to 2012-2020
**Scopus**	**1.** Hand-held OR handheld OR hand held OR hand-carried OR hand carried OR HAND OR HCU OR HHCU OR pocket size* OR portable OR miniatur* OR focus*<br>**2.** Echocardiograph* OR cardiac ultrasound<br>**3.** Rheumatic Heart Disease OR RHD<br>**#1 AND #2 AND #3**	Limited to 2012-2020
**ISI Web of Science**	**1.** Hand-held OR handheld OR hand held OR hand-carried OR hand carried OR HAND OR HCU OR HHCU OR pocket size OR pocket sized OR portable OR Miniaturization OR Miniaturized OR focused OR focus<br>**2.** Echocardiography OR Echocardiographic OR cardiac ultrasound<br>**3.** Rheumatic Heart Disease OR RHD<br>**Combine #1 AND #2 AND #3**	Limited to 2012–2020 filtering out MEDLINE
**EBSCO Host**	**S1.** Hand-held OR handheld OR hand held OR hand-carried OR hand carried OR HAND OR HCU OR HHCU OR pocket size OR pocket sized OR portable OR Miniaturization OR Miniaturized OR focused OR focus<br>**S2.** Echocardiography OR Echocardiographic OR cardiac ultrasound<br>**S3.** Rheumatic Heart Disease OR RHD<br>**S1 AND S2 AND S3**	Limited to 2012-2020
