# Supplementary material for: Standard echocardiography versus handheld echocardiography for the detection of subclinical rheumatic heart disease: a systematic review and meta-analysis of diagnostic accuracy
Source: BMJ Open. 2020 Oct 29;10(10):e038449. doi: 10.1136/bmjopen-2020-038449 (PMC7597508; doi:10.1136/bmjopen-2020-038449)
Supplement: Supplementary data [file bmjopen-2020-038449supp002.pdf]

Supplementary file 2. Forest plots and Hierarchical Summary Receiver Operating Characteristic (HSROC) plots for three categorisations of RHD; any RHD (definite or borderline), definite RHD only and borderline RHD only**.**

*All plots were generated using the Review Manager (RevMan) software package, version 5.3

Any RHD:

Study	TP	FP	FN	TN	Expertise	Sensitivity (95% CI)	Specificity (95% CI)
Beaton (2014)	37	6	4	78	Expert	0.90 [0.77, 0.97]	0.93 [0.85, 0.97]
Beaton (2015)	141	158	39	1076	Expert	0.78 [0.72, 0.84]	0.87 [0.85, 0.89]
Beaton (2016)	44	50	9	286	Non_expert	0.83 [0.70, 0.92]	0.85 [0.81, 0.89]
Mirabel (2015)	41	106	8	1062	Non_expert	0.84 [0.70, 0.93]	0.91 [0.89, 0.93]
Ploutz (2016)	32	194	11	719	Non_expert	0.74 [0.59, 0.86]	0.79 [0.76, 0.81]
Zuhlke (2016)	21	0	5	62	Expert	0.81 [0.61, 0.93]	1.00 [0.94, 1.00]

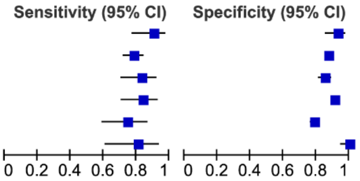

Fig 1. Forest plot of sensitivity and specificity of handheld echocardiography for any RHD

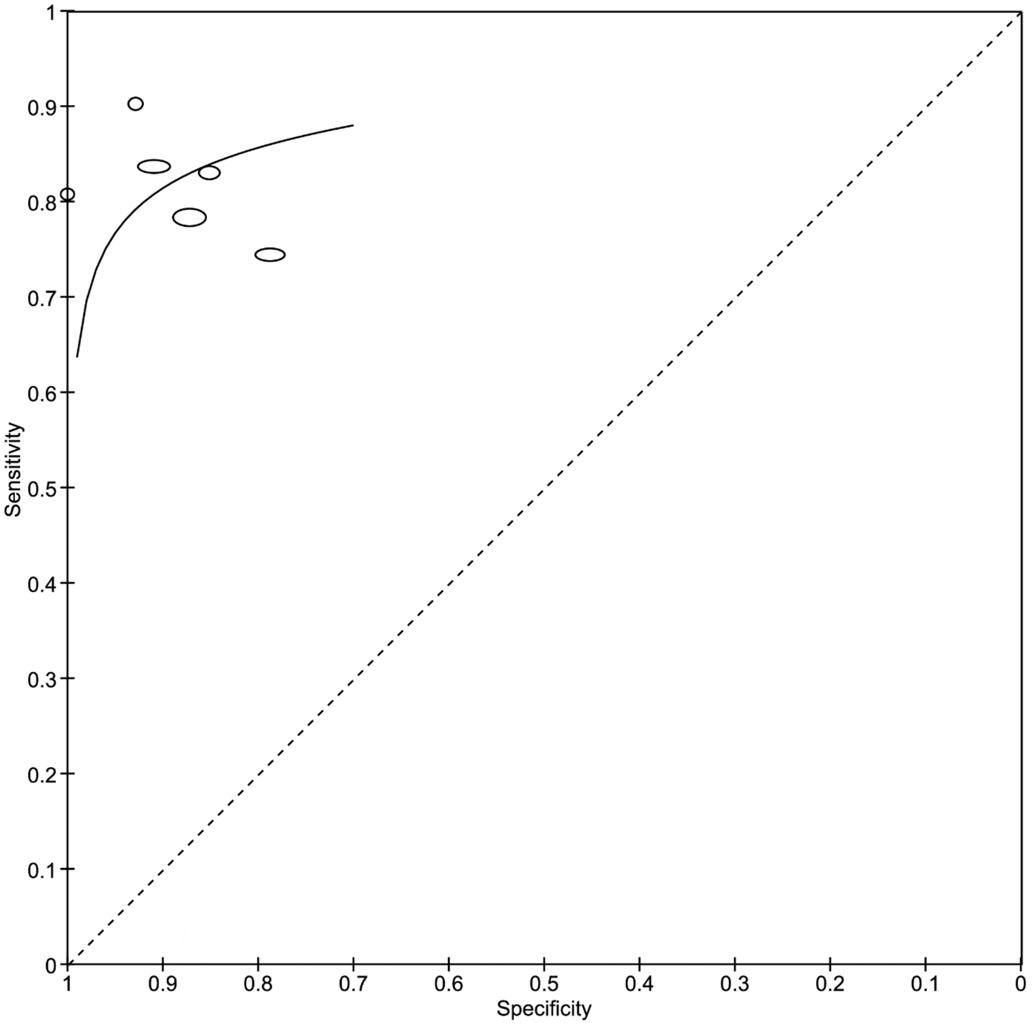

Fig 2. Summary ROC plot of sensitivity versus specificity of handheld echocardiography for any RHD

Definite RHD:

Study	TP	FP	FN	TN	Sensitivity (95% CI)	Specificity (95% CI)
Beaton (2014)	25	4	16	80	0.61 [0.45, 0.76]	0.95 [0.88, 0.99]
Beaton (2015)	46	158	1	1076	0.98 [0.89, 1.00]	0.87 [0.85, 0.89]
Mirabel (2015)	14	106	1	1062	0.93 [0.68, 1.00]	0.91 [0.89, 0.93]
Ploutz (2016)	10	194	1	719	0.91 [0.59, 1.00]	0.79 [0.76, 0.81]
Zuhlke (2016)	12	0	1	62	0.92 [0.64, 1.00]	1.00 [0.94, 1.00]

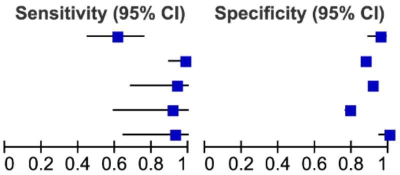

Fig 3. Forest plot of sensitivity and specificity of handheld echocardiography for definite RHD

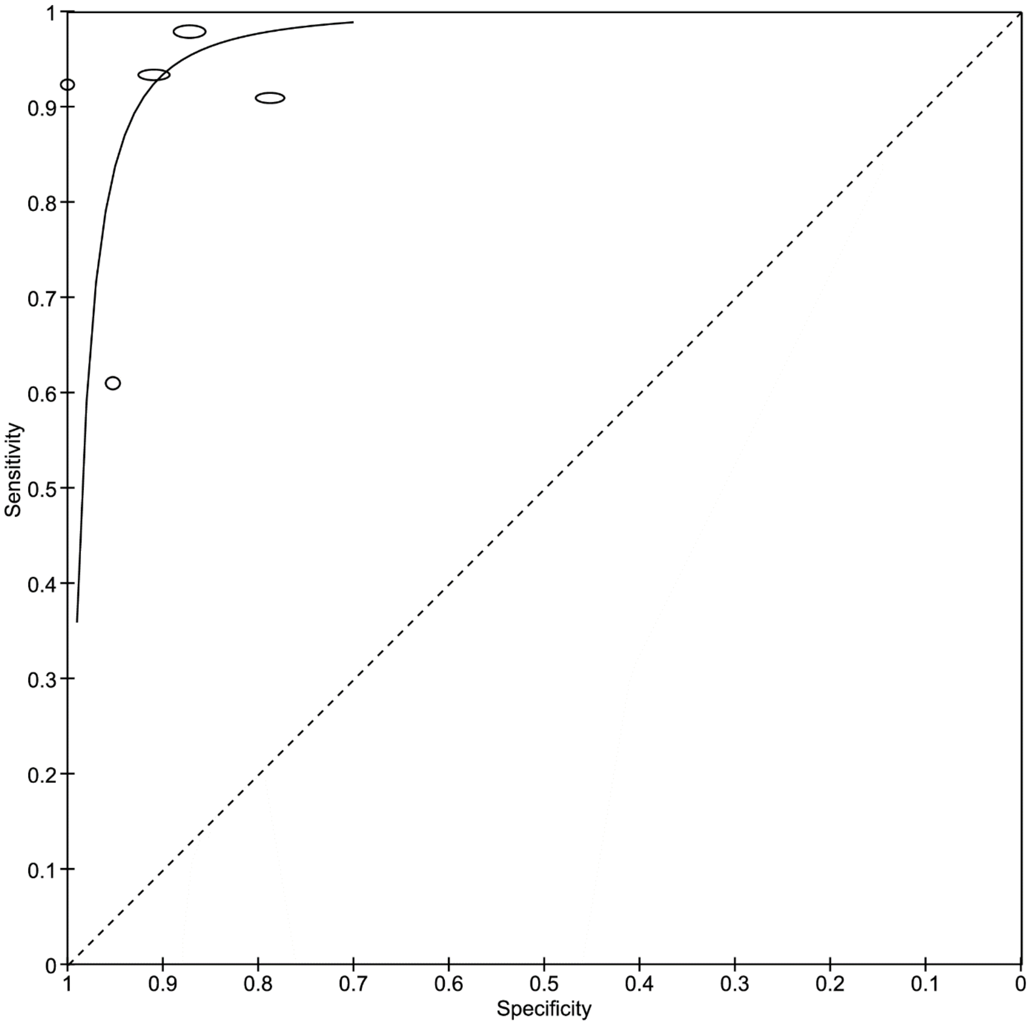

Fig 4. Summary ROC plot of sensitivity versus specificity of handheld echocardiography for definite RHD

Borderline RHD:

Study	TP	FP	FN	TN	Sensitivity (95% CI)	Specificity (95% CI)
Beaton (2014)	8	6	78	4	0.09 [0.04, 0.18]	0.40 [0.12, 0.74]
Beaton (2015)	95	158	38	1076	0.71 [0.63, 0.79]	0.87 [0.85, 0.89]
Mirabel (2015)	27	106	7	1062	0.79 [0.62, 0.91]	0.91 [0.89, 0.93]
Ploutz (2016)	22	194	10	719	0.69 [0.50, 0.84]	0.79 [0.76, 0.81]
Zuhlke (2016)	9	0	4	62	0.69 [0.39, 0.91]	1.00 [0.94, 1.00]

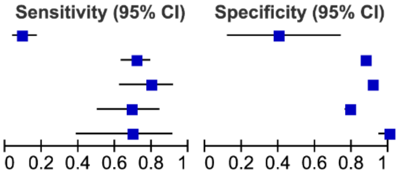

Fig 5. Forest plot of sensitivity and specificity of handheld echocardiography for borderline RHD

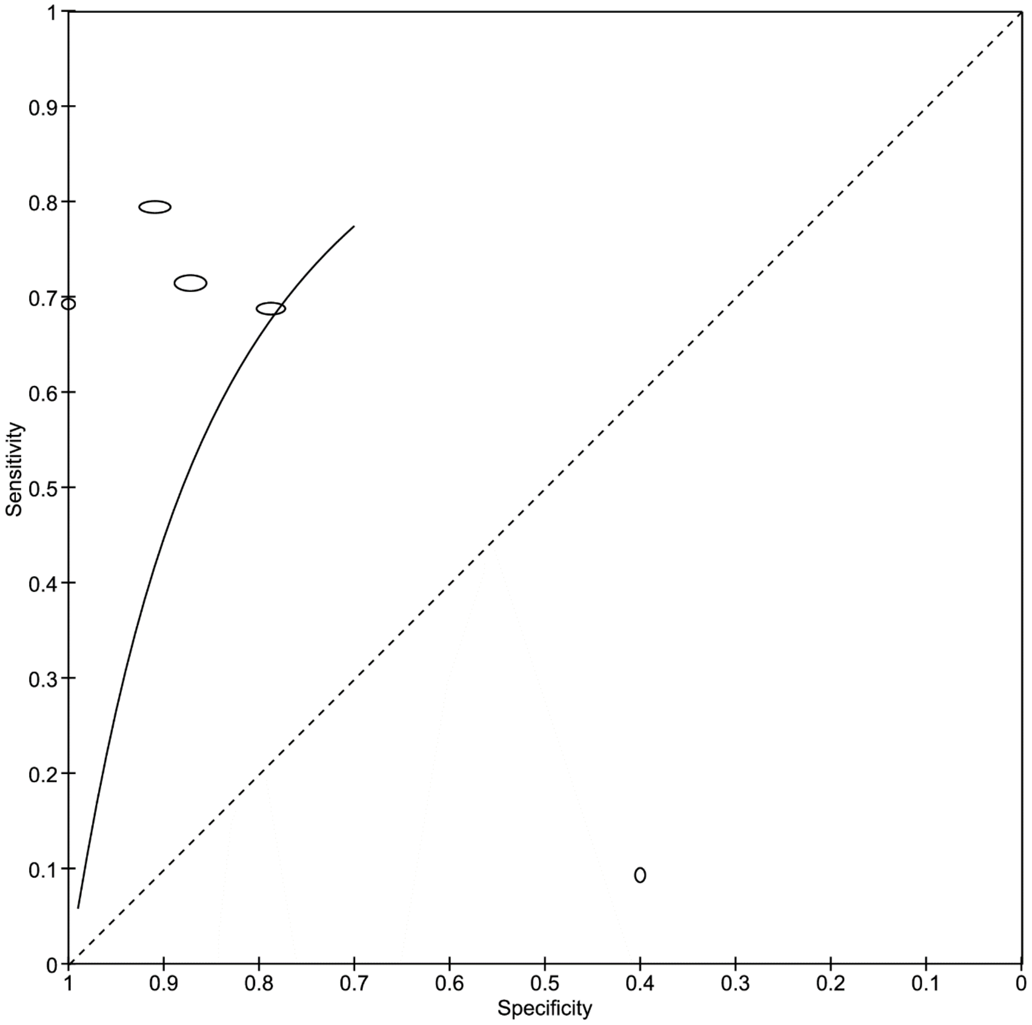

Fig 6. Summary ROC plot of sensitivity versus specificity of handheld echocardiography for borderline RHD
