# Supplementary material for: Standard echocardiography versus handheld echocardiography for the detection of subclinical rheumatic heart disease: a systematic review and meta-analysis of diagnostic accuracy
Source: BMJ Open. 2020 Oct 29;10(10):e038449. doi: 10.1136/bmjopen-2020-038449 (PMC7597508; doi:10.1136/bmjopen-2020-038449)
Supplement: Supplementary data [file bmjopen-2020-038449supp003.pdf]

Supplementary file 3. HSROC plot for subgroup analysis of echocardiographer expertise.

Subgroup Analysis:

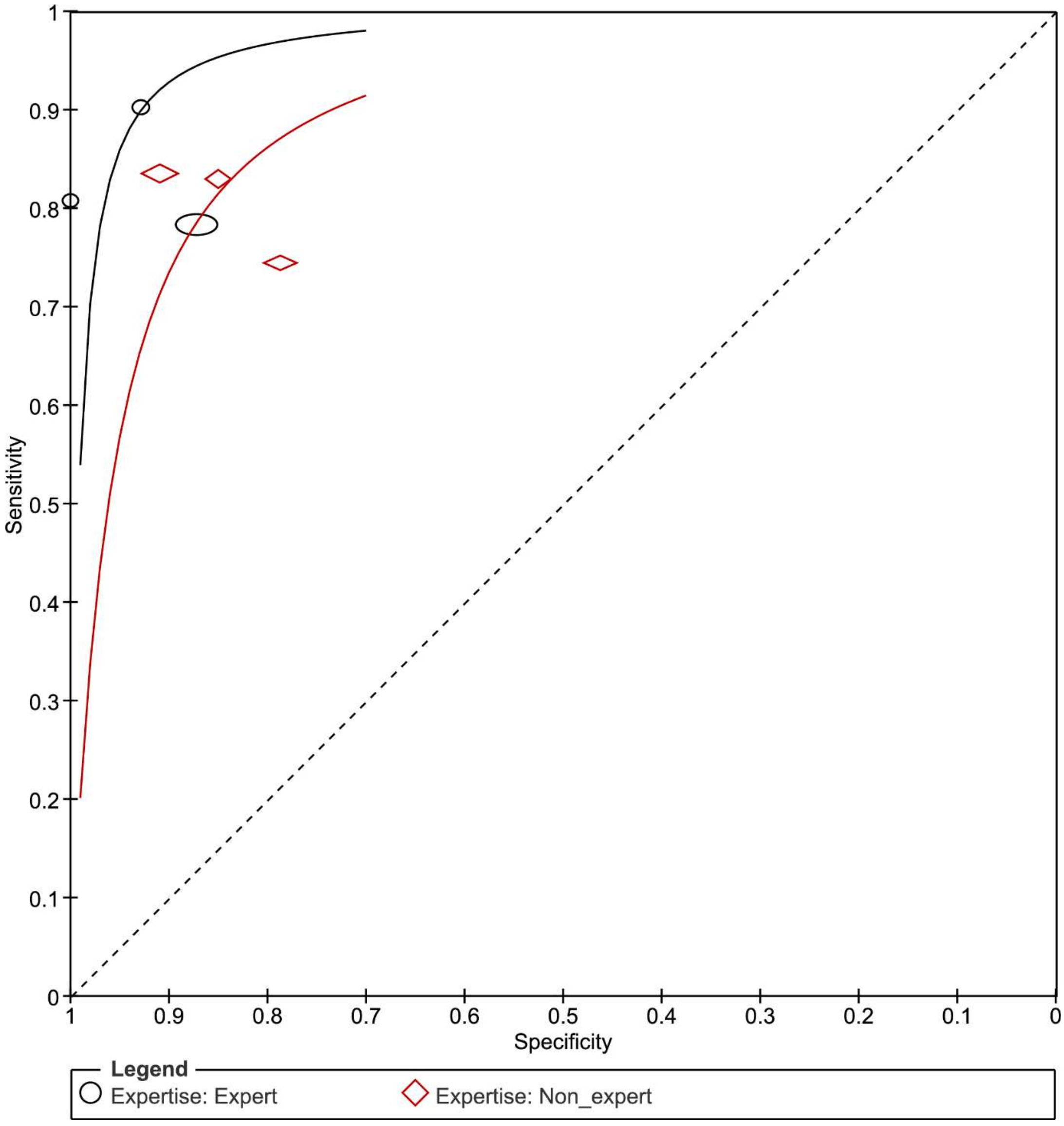

Fig 7. Summary ROC plot of sensitivity versus specificity of handheld echocardiography for any RHD according to echocardiographer expertise
