# Supplementary material for: Standard echocardiography versus handheld echocardiography for the detection of subclinical rheumatic heart disease: a systematic review and meta-analysis of diagnostic accuracy
Source: BMJ Open. 2020 Oct 29;10(10):e038449. doi: 10.1136/bmjopen-2020-038449 (PMC7597508; doi:10.1136/bmjopen-2020-038449)
Supplement: Author's manuscript [file bmjopen-2020-038449.draft_revisions.pdf]

BMJ Open is committed to open peer review. As part of this commitment we make the peer review history of every article we publish publicly available.

When an article is published we post the peer reviewers' comments and the authors' responses online. We also post the versions of the paper that were used during peer review. These are the versions that the peer review comments apply to.

The versions of the paper that follow are the versions that were submitted during the peer review process. They are not the versions of record or the final published versions. They should not be cited or distributed as the published version of this manuscript.

BMJ Open is an open access journal and the full, final, typeset and author-corrected version of record of the manuscript is available on our site with no access controls, subscription charges or pay-per-view fees (http://bmjopen.bmj.com).

If you have any questions on BMJ Open's open peer review process please email info.bmjopen@bmj.com

BMJ Open

Standard echocardiography versus handheld echocardiography for the detection of subclinical rheumatic heart disease: A systematic review and meta-analysis of diagnostic accuracy

Journal:	*BMJ Open*
Manuscript ID	bmjopen-2020-038449
Article Type:	Original research
Date Submitted by the Author:	11-Mar-2020

Complete List of Authors:	Telford, Lisa; University of Cape Town Faculty of Health Sciences, Department of Medicine, Groote Schuur Hospital; University of Cape Town Faculty of Health Sciences, Department of Paediatrics and Child Health, Red Cross War Memorial Children's Hospital<br>Abdullahi, Leila; University of Cape Town Faculty of Health Sciences, Department of Paediatrics and Child Health, Red Cross War Memorial Children's Hospital; African Institute for Development Policy (AFIDEP)<br>Ochodo, Eleanor; University of Stellenbosch Centre for Evidence-Based Health Care, Department of Global Health, Faculty of Medicine and Health Sciences<br>ZUHLKE, LIESL; University of Cape Town Faculty of Health Sciences, Division of Cardiology, Department of Paediatrics and Child Health, Red Cross War Memorial Children's Hospital; University of Cape Town Faculty of Health Sciences, Division of Cardiology, Department of Medicine, Groote Schuur Hospital<br>Engel, Mark; University of Cape Town Faculty of Health Sciences, Department of Medicine, Groote Schuur Hospital

Keywords:	Echocardiography < CARDIOLOGY, Paediatric cardiology < CARDIOLOGY, Valvular heart disease < CARDIOLOGY, PUBLIC HEALTH, Cardiovascular imaging < RADIOLOGY & IMAGING

SCHOLARONE™
Manuscripts

I, the Submitting Author has the right to grant and does grant on behalf of all authors of the Work (as defined in the below author licence), an exclusive licence and/or a non-exclusive licence for contributions from authors who are: i) UK Crown employees; ii) where BMJ has agreed a CC-BY licence shall apply, and/or iii) in accordance with the terms applicable for US Federal Government officers or employees acting as part of their official duties; on a worldwide, perpetual, irrevocable, royalty-free basis to BMJ Publishing Group Ltd ("BMJ") its licensees and where the relevant Journal is co-owned by BMJ to the co-owners of the Journal, to publish the Work in this journal and any other BMJ products and to exploit all rights, as set out in our licence.

The Submitting Author accepts and understands that any supply made under these terms is made by BMJ to the Submitting Author unless you are acting as an employee on behalf of your employer or a postgraduate student of an affiliated institution which is paying any applicable article publishing charge ("APC") for Open Access articles. Where the Submitting Author wishes to make the Work available on an Open Access basis (and intends to pay the relevant APC), the terms of reuse of such Open Access shall be governed by a Creative Commons licence – details of these licences and which Creative Commons licence will apply to this Work are set out in our licence referred to above.

Other than as permitted in any relevant BMJ Author's Self Archiving Policies, I confirm this Work has not been accepted for publication elsewhere, is not being considered for publication elsewhere and does not duplicate material already published. I confirm all authors consent to publication of this Work and authorise the granting of this licence.

For peer review only

Standard echocardiography versus handheld echocardiography for the detection of subclinical rheumatic heart disease: A systematic review and meta-analysis of diagnostic accuracy

For peer review only

Lisa H Telford[1,2], Leila H Abdullahi[2,3], Eleanor A Ochodo[4], Liesl J Zühlke[5,6], Mark E Engel[1]

Lisa H Telford

[1]Department of Medicine, Groote Schuur Hospital and University of Cape Town, Cape Town, South Africa.

[2]Department of Paediatrics and Child Health, Red Cross War Memorial Children's Hospital and University of Cape Town, Cape Town, South Africa.

Email: lisa.telford@uct.ac.za

ORCID ID: https://orcid.org/0000-0002-8554-057X

Leila H Abdullahi

[2]Department of Paediatrics and Child Health, Red Cross War Memorial Children's Hospital and University of Cape Town, Cape Town, South Africa.

[3]African Institute for Development Policy (AFIDEP), Nairobi, Kenya.

Email: leylaz@live.co.za

ORCID ID: None

Eleanor A Ochodo

[4]Centre for Evidence-based Health Care, Department of Global Health, Faculty of Medicine and Health Sciences, Stellenbosch University, Cape Town, South Africa.

Email: eochodo@sun.ac.za

ORCID ID: https://orcid.org/0000-0002-7951-3030

Liesl J Zühlke

[5]Division of Cardiology, Department of Medicine, Groote Schuur Hospital and University of Cape Town, Cape Town, South Africa.

[6]Division of Cardiology, Department of Paediatrics and Child Health, Red Cross War Memorial Children's Hospital and University of Cape Town, Cape Town, South Africa.

Email: liesl.zuhlke@uct.ac.za

ORCID ID: https://orcid.org/0000-0003-3961-2760

Mark E Engel

[1]Department of Medicine, Groote Schuur Hospital and University of Cape Town, Cape Town, South Africa.

Email: mark.engel@uct.ac.za

ORCID ID: https://orcid.org/0000-0002-1334-882

Correspondence to: Ms Lisa H Telford

Room F3, F-floor, Red Cross War Memorial Children's Hospital, Main Hospital Building, Klipfontein Road Rondebosch 7700, South Africa

+27 21 650 2438

lisa.telford@uct.ac.za

Keywords: rheumatic heart disease, echocardiography, diagnostic accuracy, systematic review, meta-analysis

Word count: 3192

For peer review only

ABSTRACT

Objective: To summarise the accuracy of handheld echocardiography (HAND) which, if shown to be sufficiently similar to that of the current gold standard, could usher in a new age of rheumatic heart disease (RHD) screening in endemic areas.

Design: Systematic review and meta-analysis.

Data sources and eligibility criteria: PubMed, Scopus, EBSCOHost and ISI Web of Science were searched for studies published from 2012 onwards. Studies assessing the accuracy of HAND compared to standard echocardiography (STAND) when performed by an experienced cardiologist in conjunction with the 2012 World Heart Federation (WHF) criteria among populations of children and adolescents living in endemic areas were included.

Main results: Six studies (N=4208) were included in the analysis. For any RHD detection, the pooled results from six studies were; sensitivity: 81.6% (95% CI: 76.5%-86.6%) and specificity: 89.8% (84.5%-95.0%). Meta-analytical results from five of the six included studies were; sensitivity: 91.1% (80.5%-100%) and specificity: 92.0% (85.6%-98.4%) for the detection of definite RHD only and sensitivity: 62.0% (31.8%-92.2%) and specificity: 82.3% (65.2%-99.5%) for the detection of borderline RHD only.

Conclusions: HAND displayed good accuracy for detecting definite RHD only and modest accuracy for detecting any RHD but demonstrated poor accuracy for the detection of borderline RHD alone. Findings from this review provide some evidence for the potential of HAND to increase access to echocardiographic screening for RHD in resource-limited and

For peer review only

remote settings, however, further research into feasibility and cost-effectiveness of wide-scale screening is still needed.

PROSPERO registration number: PROSPERO CRD42016051261.

Strengths and limitations of this study:

- Language restrictions were not imposed during the literature search to minimise the chance of missing studies.
- Data extraction was performed by two independent reviewers thereby reducing the risk of bias.
- Insufficient reporting limited our ability to adequately assess risk of bias and investigate potential sources of heterogeneity.
- The small number of included studies prevented us from performing meta-regression.

For peer review only

INTRODUCTION

Rheumatic heart disease (RHD) is an acquired permanent heart valve condition which results from an atypical immune reaction to group A streptococcal (GAS) infection typically occurring in childhood.[1,2] Disease progression leading to chronic RHD can result in irreversible heart valve damage, cardiac failure, and premature death.[3,4] RHD is, however, a preventable and treatable chronic condition which most often effects disadvantaged populations.[3,5]

Significantly, RHD can remain asymptomatic for many years, particularly during the initial stages, thereby hindering the timely implementation of penicillin prophylaxis.[6] Echocardiographic screening to identify those with subclinical disease has been advocated as a means to support secondary prevention and potentially slow disease progression to overt clinical RHD.[7,8] Yet the feasibility of wide scale echocardiographic screening remains hindered by high costs and the scarcity of trained personnel.[9] Alternative RHD screening tests which are both accurate and affordable are therefore needed in many endemic areas.

Handheld echocardiography (HAND) is a non-invasive, highly portable and comparatively less expensive device which has been presented in recent publications to be a promising alternative to standard echocardiography (STAND), despite some limitations such as a lack of spectral Doppler capabilities.[10,11] For HAND to be considered a suitable replacement for STAND, the device's accuracy needs to be similar to that of STAND.

We conducted a systematic review and meta-analysis of studies assessing the diagnostic accuracy of HAND for the detection of RHD in children and adolescents. The findings of this

For peer review only

review may offer direction to guideline developers as well as assist with the identification of gaps in diagnostic testing for RHD in endemic areas.

METHODS

This systematic review was prepared according to the Preferred Reporting Items for a Systematic Review and Meta-Analysis of Diagnostic Test Accuracy Studies (PRISMA-DTA) guidelines.[12] The protocol for this review is registered with the International Prospective Register of Systematic Reviews (PROSPERO) under the registration number CRD42016051261 and has been published in BMJ Open.

Data sources and study eligibility

Studies were considered eligible for inclusion if the following criteria were met: 1) the accuracy of HAND compared to STAND when performed by an experienced cardiologist and in conjunction with the 2012 World Heart Federation (WHF) criteria was evaluated, and 2) the sample consisted of populations of children and adolescents living in endemic areas. Only primary observational studies of either a cross-sectional, cohort or diagnostic case-control design were considered. Descriptive studies such case studies and case series were excluded as were studies reporting on the same data. Studies using non-handheld devices as the index test or criteria other than the 2012 WHF criteria in combination with STAND as the reference test were also excluded.

We conducted systematic electronic literature searches of four sources (PubMed, Scopus, EBSCOHost and ISI Web of Science) using predefined tailor-made strategies. No restrictions in terms of language were applied, however, searches were limited to articles published

For peer review only

from 2012 onwards. Both published and unpublished literature was considered eligible for inclusion. A manual search of the reference lists of all included studies as well as relevant review articles was also conducted.

The titles and/or abstracts of all identified articles were screened independently by two reviewers. During this process, and on the basis of predefined eligibility criteria, all clearly ineligible studies were excluded. Discrepancies regarding eligibility were resolved through discussion and consensus. Some authors were contacted for additional information on published data.

For peer

Data extraction and management

Using a predefined data extraction form, two reviewers independently extracted information on metrics of diagnostic accuracy: numbers of true positives (TP), false positives (FP), true negatives (TN) and false negatives (FN) as well as other covariates relating to study characteristics, population, reference and index test details, test outcome and number of missing or unavailable test results from all included studies.

Accuracy measures: sensitivity and specificity were calculated using the numbers of TP, FP, TN and FN in accordance with standard convention. Data extraction conflicts were resolved through discussion and with the assistance of a third reviewer where necessary. Information garnered through the data extraction process was used to determine each study’s quality as well as for synthesising evidence.

Assessment of methodological quality

For peer review only - http://bmjopen.bmj.com/site/about/guidelines.xhtml

A review-specific Quality Assessment of Diagnostic Accuracy Studies (QUADAS-2) tool was used to assess the risk of bias and concerns regarding applicability of all included studies.[13] The tool, encompassing four domains, was tailored to meet the specific requirements of this review. Two reviewers independently assessed the risk of bias in all included studies according to review-specific QUADAS-2 criteria. Discrepancies were resolved through discussion until consensus was reached and the assistance of a third reviewer was enlisted when necessary.

Statistical analysis and data synthesis

The Hierarchical Summary Receiver Operating Characteristic (HSROC) model was used for meta-analysis as it accounts for variations in test thresholds. Data were analysed according to three categorisations of RHD; any RHD (definite or borderline), definite RHD only and borderline RHD only. The any RHD category was selected as the main meta-analysis as it had the most complete data. We were unable to extract metrics of diagnostic accuracy for the definite and borderline RHD only categories from Beaton, 2016 and therefore excluded this study from these meta-analyses. We chose to use Nurse A's results for Mirabel, 2015 since Nurse A and Nurse B both interpreted the same HAND images which prevented the pooling of data.

Data from Zühlke, 2016 were included in the analysis and synthesis of data even though the age range of participants fell outside the predefined range for eligibility. It was determined that this study should be included, regardless, since the data overall were quite few and the variation in age was not significant enough to warrant exclusion. However, data from

For peer review only - http://bmjopen.bmj.com/site/about/guidelines.xhtml

Zühlke, 2016 were excluded from all summary estimates of disease prevalence since this study used a nested case-control design which predetermines disease prevalence by design.

Heterogeneity was examined for the main meta-analysis only. We were only able to investigate the relationship between test accuracy and echocardiographer expertise through subgroup analysis. A sensitivity analysis was performed instead of subgroup analysis for the categorical covariates; HAND protocol and geographic location due to the skewed distribution of studies within each subgroup. We were unable to perform meta-regression for the covariates; age and sex due to insufficient and inadequately reported data.[14] We were also unable to conduct a sensitivity analysis on risk of bias since no studies were found to have a high risk of bias. All plots were generated using the Review Manager (RevMan) software package, version 5.3.[15] Meta-analysis was performed using SAS® software, version 9.4.[16]

We did not investigate publication bias as methods of assessing publication bias for studies of diagnostic accuracy are still being developed. While the Deeks test has been suggested for use in diagnostic accuracy studies, the test has low power for detecting asymmetry in funnel plots, particularly when a large amount of heterogeneity is present.[14]

RESULTS

Results of the search

Results of the literature search are reported in accordance with the PRISMA Statement and the study selection process is illustrated in Fig 1.[17] All electronic searches were performed by two independent reviewers on September 27th, 2017. Combined, the search yielded a total of ninety two records, of which nine were duplicates. A total of sixty seven were

For peer review only

excluded based on title or abstract leaving sixteen for full-text review. Nine studies were excluded on the basis of a full-text review whilst an additional study was also excluded after consultation with study authors. Six studies which met the predefined eligibility criteria were included in this review.

Figure 1. PRISMA flow diagram illustrating study identification, selection, eligibility and inclusion. *From* Moher et al.[17]

The same search was re-run on March 3rd, 2020 to check for any additional eligilble studies. Only one potentially eligible study [18,19] was found but has been excluded on the basis of being an abstract only publication with no full-text available for review.

Included studies

A summary of notable characteristics of all included studies [20–25] is shown in Table 1. One study did not avoid a case-control design, however, cases and controls were sampled from the same population. Research has shown that case-control studies which use alternative diagnosis controls, controls from non-endemic areas or confirmed disease-free (healthy) controls tend to overestimate specificity.[26]

Significantly, all but two studies were conducted in Africa. Screening was performed in RHD endemic areas among children and adolescents with most studies being school-based. Combined, all six studies included a total of 4208 participants of which 54% were female. The pooled mean age of participant's was 10.8 years (SD: ±1.9).

All included studies used the same make of handheld device; the Vscan machine (General Electric, Medical Systems, Milwaukee, Wisconsin, USA) paired with a 1.7 to 3.4 MHz transducer. These machines provide both two-dimensional (2D) and colour imaging on an

For peer review only

integrated 8.9cm display.[21,23] Frame rates range from 25 to 30 Hz for greyscale imaging and 12 to 16 Hz for colour Doppler.[20,24] Vscan machines are, however, limited by a lack of spectral Doppler capabilities.[21]

For peer review only

Table 1. Summary of characteristics of included studies [ordered alphabetically by study author]

Study	Study Design	City, Country (Classification¤)	Recruitment Site/Setting	Participant Selection Method	Sample Size (N)	% Female	Mean Age (years)	SD
**Beaton, 2014**[20]	Spiked cohort	Kampala, Uganda (Low-income)	A School and the Mulago Hospital Complex	Unclear	125	55.2	10.8¥	-
**Beaton, 2015**[21]	Cross-sectional	Gulu, Uganda (Low-income)	5 public schools	A random 10% subset of the entire sample plus any child with mitral or aortic regurgitation were preselected to receive HAND	1420	53	10.8	±2.6
**Beaton, 2016**[22]	Cross-sectional	Belo Horizonte, Brazil (Upper-middle income)	2 primary and 3 secondary public schools	A subset of the sample containing all STAND abnormals plus a random 25% of all STAND normals were preselected for HAND	397	49.1	13.9	±2.6
**Mirabel, 2015**[23]	Cross-sectional	Nouméa, New Caledonia (High-income)	Primary schools	Consecutive	1217	50.5	9.6	±0.5
**Ploutz, 2016**[24]	Cross-sectional	Gulu, Uganda (Low-income)	2 primary schools	Consecutive	956	60.7	11.1	±2.5
**Zühlke, 2016**[25]	Nested case-control	Cape Town, South Africa (Upper-middle income)	Schools	Unclear	93	68.8	17¥†	-
**Total**					4208	54	10.8	±1.9

¤According to the World Bank's economic classification system; ¥excluded from pooled mean & SD calculations (incomplete or incomparable data); †median age (mean age not available)

Excluded studies

Ten studies [9,10,27–34] were excluded during full-text screening. Reasons for exclusion included abstract only publications, the use of ineligible reference or index tests, the use of duplicate data, not specifying the test threshold a-priori, and not being a study of diagnostic accuracy.

Methodological quality of included studies

Overall, only two of the six included studies were assessed as having a low risk of bias while the risk in the remaining four was unclear. Two studies had participant selection bias concerns. Of these both failed to adequately describe participant enrolment methods whilst one also did not avoid a case-control design. The risk of bias in terms of flow and timing was unclear in two studies. Of these, one study did not include all participants in the analysis due to technical difficulties while the time interval between the index and reference test was unclear in the other. Overall, time intervals between index and reference tests were poorly described. Likewise, reporting of quality control of the index test was uniformly poor across all included studies. Concerns regarding applicability were low in all six studies.

Findings

For any RHD

A total of six evaluations of HAND for any RHD were performed with data from six studies and a total of 4208 participants. Pooled prevalence of any RHD from five included studies was 12% (95% confidence interval (CI): 6%-19%). The forest plot revealed little variation in

For peer review only

estimates of sensitivity and specificity and the HSROC plot revealed moderate accuracy of the test. Meta-analytical sensitivity and specificity (95% CI) of data at mixed thresholds were 82% (77%-87%) and 90% (85%-95%) respectively.

For definite RHD

A total of five evaluations of HAND for definite RHD were performed with data from five studies and a total of 3588 participants. Pooled prevalence of definite RHD from four included studies was 6% (95% CI: 2%-12%). The forest plot revealed some variation in estimates of specificity while estimates of sensitivity were largely homogenous with the exception of a single outlier. The HSROC plot indicated good accuracy of the test. Meta-analytical sensitivity and specificity (95% CI) of data at mixed thresholds were 91% (81%-100%) and 92% (86%-98%) respectively.

For borderline RHD

A total of five evaluations of HAND for borderline RHD were performed with data from five studies and a total of 3685 participants. Pooled prevalence of borderline RHD from four included studies was 20% (95% CI: 6%-39%). The forest plot revealed some variation in estimates of specificity while estimates of sensitivity were largely homogenous with the exception of a single outlier. The HSROC plot indicated poor accuracy of the test. Meta-analytical sensitivity and specificity (95% CI) of data at mixed thresholds were 62% (32%-92%) and 82% (65%-100%) respectively.

For peer review only

Investigations of heterogeneity

Heterogeneity or variation between studies was investigated both visually as well as through subgroup analysis for the main meta-analysis only. We were only able to perform this analysis for the any RHD category as the data were too few to enable model convergence for the definite and borderline RHD only categories.

Co-variates in the models

We were only able to use one of the five pre-specified covariates to investigate heterogeneity due to insufficient and inadequately reported data. HAND echocardiographer expertise (expert vs non-expert) was investigated as a possible source of heterogeneity through subgroup analysis. Half of all included studies evaluated the accuracy of HAND when performed and interpreted by trained non-experts while the other half assessed its accuracy in the hands of experts.

Subgroup and sensitivity analyses

A subgroup analysis was performed to investigate variations in echocardiographer expertise as a potential source of heterogeneity. Since no studies were found to have a high risk of bias we did not explore the effect of excluding such studies on the accuracy of summary estimates**.** Sensitivity analyses were, however, conducted to investigate the effect of removing a single study on summary estimates of sensitivity and specificity for the covariates; geographic location and HAND protocol.

A subgroup analysis for the covariate; echocardiographer expertise, as shown in Table 2, revealed that both sensitivity (82.54% vs 80.76%) and specificity (94.57% vs 85.71%) were

higher for any RHD detection using HAND when tests were performed and interpreted by experts compared to non-experts.

Sensitivity analyses (see table 2) were performed to investigate the effect of excluding a) the single high-income country study and b) the study which employed a single view protocol on the accuracy of summary estimates. We found that both sensitivity (81.17% vs 80.4%) and specificity (90.07% vs 87.15%) increased compared to the overall analysis when only low- and middle-income country studies were considered whereas both sensitivity (80.98% vs 85.35%) and specificity (87.46% vs 88.8%) decreased in comparison with the overall analysis when only studies which employed multiple view protocols were considered.

Table 2. Sources of heterogeneity for handheld echocardiography for any RHD

**Group**	**Covariate**	**Subgroup**	**n (N=6)**	**Median Pooled Sensitivity** (95% Crl)	**Median Pooled Specificity** (95% Crl)
**Overall**			6	**81.56% (76.52-86.61)**	**89.75% (84.48-95.01)**
**Subgroup Analysis**	HAND Interpreter Expertise	Expert	3	82.54% (74.71-90.37)	94.57% (87.52-100)
		Non-expert	3	80.76% (73.18-88.33)	85.71% (79.91-91.51)
**Sensitivity Analysis**	Geographic Location	Overall		80.40% (64.96-95.84)	87.15% (76.35-97.96)
		Low- and middle-income countries	5	81.17% (75.63-86.71)	90.07% (82.98-97.15)
	HAND Protocol	Overall		85.35% (80.60-90.09)	88.80% (84.55-93.06)
		Multiple views	5	80.98% (75.38-86.59)	87.46% (83.24-91.67)

Abbreviations: HAND, handheld echocardiography; Crl, credible interval; n, number of studies; N, total number of included studies

Table 3. Summary of findings

What is the diagnostic accuracy of handheld echocardiography in detecting any RHD (definite or borderline)?	
**Patients/Population**	People residing in areas endemic for RHD (6 out of 6 studies)
**Prior testing with echo**	Yes (2 studies), No (5 studies)
**Settings**	5 out of 6 screening studies were field setting (communities and schools) based while 1 study was half hospital registry follow-up, half school based.<br>4 of the 6 studies were conducted in Africa with 3 of those from Uganda.
**Index test(s)**	General Electric (GE) Vscan handheld machine (6 out of 6 studies)
**Reference standard**	Standard echocardiography (2D, continuous-wave, and colour-Doppler echocardiography) performed by an experienced imager and in conjunction with the 2012 WHF criteria (6 out of 6 studies).<br>Test brands included; GE Vivid-I ultrasound machine (2 studies)<br>; GE Vivid-Q ultrasound machine (2 studies)<br>; Philips CX-50 ultrasound machine (1 study)

DISCUSSION

Summary of main findings

We evaluated the accuracy of HAND for three distinct disease categories and found that, overall, the test was both sensitive and specific for detecting definite RHD only and moderately accurate for detecting any RHD but demonstrated insufficient accuracy for detecting borderline RHD alone.

Findings from this review provide some evidence for the potential of HAND to increase access to echocardiographic screening for RHD in resource-limited and remote settings. A summary of the accuracy estimates produced by meta-analysis using the HSROC method is included in table 3.

review only

	; Either a GE Vivid-I or Q or Philips CX-50 ultrasound machine (1 study)	
**Importance**	AND is being used as first line replacement for STAND in disease screening programmes for RHD, as it is comparably inexpensive, quick, user friendly, easy to interpret, and may have similar sensitivity to STAND.	
**Studies**	Cross-sectional (n=4), spiked cohort (n=1) and nested case-control (n=1) studies. More than half (n=4) of all included studies did not explicitly state the study design used and were thus assigned a study design based on other reported characteristics and participant enrolment methods used.	
**Quality concerns**	Poor reporting of study design, participant characteristics and pre-test probability were common concerns. For the majority of studies the risk of bias was unclear in terms of 'patient selection' and 'flow and timing'. Concerns regarding applicability were low in all included studies.	
**Test types**	**Number of participants*** (N)	**Summary estimates** (95% credible CI)
HAND for **any RHD**	4208 (6)	Sensitivity; 81.56% (76.52-86.61)<br>Specificity; 89.75% (84.48-95.01)
HAND for **definite RHD**	3588 (5)	Sensitivity; 91.06% (80.46-100)<br>Specificity; 91.96% (85.57-98.36)
HAND for **borderline RHD**	3685 (5)	Sensitivity; 62.01% (31.8-92.22)<br>Specificity; 82.33% (65.15-99.52)
Abbreviations: CI, confidence interval; HAND, handheld echocardiography; N, number of studies; RHD, rheumatic heart disease; STAND, standard echocardiography *Excluding participants with other diagnoses on STAND		

Strengths and limitations of this review

Strengths

We have evaluated and summarised the accuracy of HAND for the detection of RHD in endemic areas, making the review relevant to current global agendas. This review also serves to highlight the existing gaps in evidence for which further research could be beneficial. We did not impose any limits in terms of language during the literature search so

as to minimise the chance of missing studies. Data extraction was performed by two independent reviewers thereby reducing the risk of bias.

Limitations

There were a number of shortcomings of this review, which included;

Eligibility: We were unable to include studies which used STAND in conjunction with criteria other than the 2012 WHF criteria as the reference standard which limited the number of studies eligible for inclusion.

Quality of included studies: Insufficient reporting of participant characteristics and study methods including study design, participant selection and test timing restricted our ability to adequately assess risk of bias and investigate potential sources of heterogeneity.

Paucity of data: Insufficient and inadequately reported data as well as the presentation of aggregate data limited the scope of our investigations of heterogeneity while the small number of included studies prevented us from performing meta-regression. Overall, the findings from this review may lack power due to the small sample size.

Applicability of findings to the review question

Concerns regarding the applicability of included studies to the review question were considered low according to review-specific QUADAS-2 criteria. Since all but one were conducted in low- or middle-income countries, and all studies with one exception were conducted in field settings, the results of this review are applicable for use in endemic areas for which screening programmes are frequently targeted. However, our limited assessment

For peer review only

of risk of bias and investigations into sources of heterogeneity such as age and gender due to insufficient reporting may lessen the applicability of findings to the review question.

In the context of disease control programmes, being able to demonstrate variation in test accuracy associated with factors such as age and gender would be beneficial for policy makers. Fully understanding included studies' risk of bias would also assist in objectively assessing the strength of evidence. For these reasons, prospective authors of diagnostic test accuracy studies are urged to make use of the Standards for Reporting of Diagnostic Accuracy Studies (STARD) guidelines [35] when reporting methods of study design and conduct.

CONCLUSION

This review provides a summary of the accuracy of HAND for the detection of RHD. In populations of children and adolescents living in RHD endemic areas, HAND is both sensitive and specific for detecting definite RHD. The device is less accurate in detecting any RHD and demonstrates substandard accuracy for the detection of borderline RHD only. Nonetheless, this test may hold value as a replacement for first line screening due to its high sensitivity for definite RHD detection and adequate accuracy for any RHD detection.

Implications for practice

We have summarised the accuracy of HAND when used as a screening tool, however, the device's potential value in terms of diagnostics has yet to be established. We therefore posit that HAND could be recommended as an acceptable replacement test for first line screening

For peer review only

in endemic areas provided a standardised set of device-specific diagnostic criteria are developed.

Another key consideration is the applicability of these findings for recommendations to integrate screening into routine clinical practice. A recent publication has reviewed the cost-effectiveness of screening in high-risk populations [36] and determined that screening all indigenous Australian 5 to 12 year-olds in half of their communities in alternate years was found to be cost-effective, if RHD can be detected at least 2 years earlier. However, this result was sensitive to a number of assumptions, including local costs and context. Other cost-effectiveness models have also suggested modestly improved outcomes at lower cost.[37] Neither of these studies included the significant cost-reduction using HAND instead of STAND, hence, we highly recommend adding a cost-effectiveness analysis into proposed new screening studies.

Finally, our findings demonstrate comparable results by non-experts, this has also been demonstrated in several other reports [28,38], but again there are no detailed cost-effectiveness analyses using non-experts and HAND.

Implications for research

The findings of this review highlight the need for a new set of evidence-based guidelines tailored to the capabilities of HAND in order to maximise the device's diagnostic potential. Further studies assessing the diagnostic accuracy of HAND when using a standardised protocol are needed as is further research into the feasibility, cost-effectiveness and consequences of implementing wide scale screening programs. Furthermore, the development of standardised training programs for non-experts is recommended as

For peer review only

screening for RHD in endemic areas inevitably rests on the success of task shifting.[24] We conclude that while HAND has been shown to be sufficiently accurate for the detection of RHD there is still a need for further research before its' wide scale use can be endorsed.

Author affiliations

[1]Department of Medicine, Groote Schuur Hospital and University of Cape Town, Cape Town, South Africa.

[2]Department of Paediatrics and Child Health, Red Cross War Memorial Children's Hospital and University of Cape Town, Cape Town, South Africa.

[3]African Institute for Development Policy (AFIDEP), Nairobi, Kenya.

[4]Centre for Evidence-based Health Care, Department of Global Health, Faculty of Medicine and Health Sciences, Stellenbosch University, Cape Town, South Africa.

[5]Division of Cardiology, Department of Medicine, Groote Schuur Hospital and University of Cape Town, Cape Town, South Africa.

[6]Division of Cardiology, Department of Paediatrics and Child Health, Red Cross War Memorial Children's Hospital and University of Cape Town, Cape Town, South Africa.

Author contributions

LJZ and MEE jointly conceived of the study and provided content advice. LHT and LHA performed the systematic review of the literature, independently extracted the data and are responsible for the quality control of study selection. EAO provided methodological advice and was responsible for conducting the meta-analysis. LHT significantly contributed to the interpretation of the data and wrote the first draft of the manuscript. All authors contributed to editing subsequent versions of the manuscript.

For peer review only

Funding

LJZ, LHT and LHA all receive funding from the Medtronic Foundation through support to RHD Action. LHT receives support from the National Research Foundation of South Africa (NRFSA) through its internship programme. LJZ is supported by both the NRFSA and Medical Research Council (MRC).

Competing interests

None declared.

Patient consent for publication

Not required.

Provenance and peer review

Not commissioned; externally peer reviewed.

Data availability statement

Dataset, https://doi.org/10.25375/uct.11902077.v1, and figures available from the ZivaHub repository. All other relevant data are included in the article or uploaded as supplementary information.

ORCID iDs

Lisa H Telford https://orcid.org/0000-0002-8554-057X

Eleanor A Ochodo https://orcid.org/0000-0002-7951-3030

For peer review only

Liesl J Zühlke https://orcid.org/0000-0003-3961-2760

Mark E Engel https://orcid.org/0000-0002-1334-882

References

1. Marijon E, Mirabel M, Celermajer DS, Jouven X. Rheumatic heart disease. *Lancet* [Internet] 2012;379(9819):953–64. Available from: https://doi.org/10.1016/S0140-6736(11)61171-9

2. Zühlke LJ, Beaton A, Engel ME, Hugo-Hamman CT, Karthikeyan G, Katzenellenbogen JM, et al. Group A Streptococcus, Acute Rheumatic Fever and Rheumatic Heart Disease: Epidemiology and Clinical Considerations. *Curr Treat Options Cardiovasc Med* [Internet] 2017;19(2):15. Available from: https://doi.org/10.1007/s11936-017-0513-y

3. World Heart Federation. Rheumatic Heart Disease: Neglected NCD of Poverty (Factsheet). Geneva: World Heart Federation; 2013.

4. Seckeler MD, Hoke TR. The worldwide epidemiology of acute rheumatic fever and rheumatic heart disease. *Clin Epidemiol* 2011;3(1):67–84.

5. Dougherty S, Khorsandi M, Herbst P. Rheumatic heart disease screening: Current concepts and challenges. *Ann Pediatr Cardiol* [Internet] 2017;10(1):39. Available from: http://www.annalspc.com/text.asp?2017/10/1/39/197051

6. Moloi AH, Mall S, Engel ME, Stafford R, Zhu ZW, Zuhlke LJ, et al. Rheumatic Heart Disease Epidemiology and Health Systems Barriers and Facilitators. *Glob Heart* 2017;12(1):5-15.e3.

For peer review only

7. Zühlke LJ, Watkins DA, Perkins S, Wyber R, Mwangi J, Markbreiter J, et al. A Comprehensive Needs Assessment Tool for Planning RHD Control Programs in Limited Resource Settings. *Glob Heart* [Internet] 2017;12(1):25–31. Available from: http://dx.doi.org/10.1016/j.gheart.2016.10.028

8. Palafox B, Mocumbi AO, Kumar RK, Ali SKM, Kennedy E, Haileamlak A, et al. The WHF Roadmap for Reducing CV Morbidity and Mortality Through Prevention and Control of RHD. *Glob Heart* [Internet] 2017;12(1):47–62. Available from: http://dx.doi.org/10.1016/j.gheart.2016.12.001

9. Lu JC, Sable C, Ensing GJ, Webb C, Scheel J, Aliku T, et al. Simplified rheumatic heart disease screening criteria for handheld echocardiography. *J Am Soc Echocardiogr* [Internet] 2015;28(4):463–9. Available from: http://dx.doi.org/10.1016/j.echo.2015.01.001

10. Godown J, Lu JC, Beaton A, Sable C, Mirembe G, Sanya R, et al. Handheld Echocardiography Versus Auscultation for Detection of Rheumatic Heart Disease. *Pediatrics* [Internet] 2015;135(4):e939–44. Available from: http://search.ebscohost.com/login.aspx?direct=true&db=cmedm&AN=25780068&site=ehost-live

11. Saxena A. Rheumatic heart disease screening by "point-of-care" echocardiography: an acceptable alternative in resource limited settings? *Transl Pediatr* [Internet] 2015;4(3):210–3. Available from: http://www.ncbi.nlm.nih.gov/pubmed/26835377

12. McInnes MDF, Moher D, Thombs BD, McGrath TA, Bossuyt PM, and the PRISMA-DTA Group, et al. Preferred Reporting Items for a Systematic Review and Meta-analysis of

For peer review only

Diagnostic Test Accuracy Studies: The PRISMA-DTA Statement. *JAMA* [Internet] 2018;319(4):388–96. Available from: http://jama.jamanetwork.com/article.aspx?doi=10.1001/jama.2017.19163

13. Whiting PF, Rutjes AWS, Westwood ME, Mallet S, Deeks JJ, Reitsma JB, et al. Research and Reporting Methods Accuracy Studies. *Ann Intern Med* 2011;155(4):529–36.

14. Macaskill P, Gatsonis C, Deeks JJ, Harbord RM, Takwoingi Y. Analysing and Presenting Results. In: Deeks JJ, Bossuyt PM, Gatsonis C, editors. Cochrane Handbook for Systematic Reviews of Diagnostic Test Accuracy Version 10 [Internet] The Cochrane Collaboration; 2010. Available from: http://srdta.cochrane.org/

15. Review Manager (RevMan). Copenhagen: The Nordic Cochrane Centre, The Cochrane Collaboration; 2014.

16. SAS/STAT Software. SAS Institute Inc.; 2011.

17. Moher D, Liberati A, Tetzlaff J, Altman DG, Altman DG, Antes G, et al. Preferred Reporting Items for Systematic Reviews and Meta-Analyses: The PRISMA Statement. *PLoS Med* [Internet] 2009;6(7):e1000097. Available from: http://www.ncbi.nlm.nih.gov/pubmed/19621072

18. Francis J, Fairhurst H, Kaethner A, Whalley G, Ryan C, Brown S, et al. Handheld PLAX View Echocardiography by Healthworkers for Detection of RHD. *Hear Lung Circ* [Internet] 2019;28:S51–2. Available from: http://dx.doi.org/10.1016/j.hlc.2019.05.133

19. Francis J, Fairhurst H, Kaethner A, Whalley G, Ryan C, Dos Santos J, et al. Single

For peer review only

parasternal long axis echocardiography by briefly trained health workers using handheld devices for detection of rheumatic heart disease: a prospective study of diagnostic accuracy. *Eur Heart J* 2019;40(Supplement_1):3133.

20. Beaton A, Aliku T, Okello E, Lubega S, McCarter R, Lwabi P, et al. The utility of handheld echocardiography for early diagnosis of rheumatic heart disease. *J Am Soc Echocardiogr* [Internet] 2014;27(1):42–9. Available from: http://dx.doi.org/10.1016/j.echo.2013.09.013

21. Beaton A, Lu JC, Aliku T, Dean P, Gaur L, Weinberg J, et al. The utility of handheld echocardiography for early rheumatic heart disease diagnosis: a field study. *Eur Hear J - Cardiovasc Imaging* [Internet] 2015;16(5):475–82. Available from: https://academic.oup.com/ehjcimaging/article-lookup/doi/10.1093/ehjci/jeu296

22. Beaton A, Nascimento BR, Diamantino AC, Pereira GTR, Lopes ELV, Miri CO, et al. Efficacy of a Standardized Computer-Based Training Curriculum to Teach Echocardiographic Identification of Rheumatic Heart Disease to Nonexpert Users. *Am J Cardiol* [Internet] 2016;117(11):1783–9. Available from: http://dx.doi.org/10.1016/j.amjcard.2016.03.006

23. Mirabel M, Bacquelin R, Tafflet M, Robillard C, Huon B, Corsenac P, et al. Screening for Rheumatic Heart Disease: Evaluation of a Focused Cardiac Ultrasound Approach. *Circ Cardiovasc Imaging* [Internet] 2015;8(1):e002324–e002324. Available from: http://www.ncbi.nlm.nih.gov/pubmed/25567654

24. Ploutz M, Lu JC, Scheel J, Webb C, Ensing GJ, Aliku T, et al. Handheld echocardiographic screening for rheumatic heart disease by non-experts. *Heart*

For peer review only

[Internet] 2016;102(1):35–9. Available from: http://10.0.4.112/heartjnl-2015-308236

25. Zühlke LJ, Engel ME, Nkepu S, Mayosi BM. Evaluation of a focussed protocol for hand-held echocardiography and computer-assisted auscultation in detecting latent rheumatic heart disease in scholars. *Cardiol Young* [Internet] 2016;26(06):1097–106. Available from: https://www.scopus.com/inward/record.uri?eid=2-s2.0-84943741614&partnerID=40&md5=816a5cc70c6958d0909d9c3f9594c25a

26. Rutjes AWS, Reitsma JB, Vandenbroucke JP, Glas AS, Bossuyt PMM. Case-control and two-gate designs in diagnostic accuracy studies. *Clin Chem* 2005;51(8):1335–41.

27. Mirabel M, Celermajer DS, Ferreira B, Tafflet M, Perier M-C, Karam N, et al. Screening for rheumatic heart disease: evaluation of a simplified echocardiography-based approach. *Eur Heart J Cardiovasc Imaging* [Internet] 2012;13(12):1024–9. Available from: https://academic.oup.com/ehjcimaging/article-lookup/doi/10.1093/ehjci/jes077

28. Colquhoun SM, Carapetis JR, Kado JH, Reeves BM, Remenyi B, May W, et al. Pilot study of nurse-led rheumatic heart disease echocardiography screening in Fiji--a novel approach in a resource-poor setting. *Cardiol Young* [Internet] 2013;23(4):546–52. Available from: http://www.ncbi.nlm.nih.gov/pubmed/23040535

29. Godown J, Lu JC, Beaton A, Sable C, Mirembe G, Sanya R, et al. Handheld Echocardiography Improves Detection of Rheumatic Heart Disease in Ugandan Schoolchildren Compared to Auscultation. *Circulation* [Internet] 2014;130(2). Available from: http://circ.ahajournals.org/content/130/Suppl_2/A11256.short

30. Lu JC, Sable C, Ensing GJ, Webb CC, Scheel J, Aliku T, et al. Abstract 11138: Simplified

For peer review only

Rheumatic Heart Disease Screening Criteria for Handheld Echocardiography. *Circulation* [Internet] 2014;130(Suppl 2):A11138 LP-A11138. Available from: http://circ.ahajournals.org/content/130/Suppl_2/A11138.abstract

31. Engelman D, Kado JH, Reményi B, Colquhoun SM, Carapetis JR, Donath S, et al. Focused cardiac ultrasound screening for rheumatic heart disease by briefly trained health workers: a study of diagnostic accuracy. *Lancet Glob Heal* [Internet] 2016;4(6):e386–94. Available from: http://linkinghub.elsevier.com/retrieve/pii/S2214109X16300651

32. Hardie J, Goodman N, Khan N, Barasa A, Ngunga M, Jutley R. Abstract: P1373 Feasibility of focused cardiac ultrasound by non-expert operators as a strategy for a rheumatic heart disease screening programme. *Eur Heart J* [Internet] 2016;37:241. Available from: http://doi.wiley.com/10.1111/ctr.12644

33. Lopes EL, Beaton AZ, Nascimento BR, Tompsett A, Dos Santos JP, Perlman L, et al. Telehealth solutions to enable global collaboration in rheumatic heart disease screening. *J Telemed Telecare* [Internet] 2016; Available from: http://search.ebscohost.com/login.aspx?direct=true&db=cmedm&AN=27815494&site=ehost-live

34. Diamantino A, Beaton A, Aliku T, Oliveira K, Oliveira C, Xavier L, et al. A focussed single-view hand-held echocardiography protocol for the detection of rheumatic heart disease. *Cardiol Young* [Internet] 2017;1–10. Available from: https://www.cambridge.org/core/product/identifier/S1047951117001676/type/journal_article

For peer review only

35. Bossuyt PM, Reitsma JB, Bruns DE, Gatsonis CA, Glasziou PP, Irwig L, et al. STARD 2015: an updated list of essential items for reporting diagnostic accuracy studies. *BMJ* [Internet] 2015;277(3):h5527. Available from: http://www.bmj.com/lookup/doi/10.1136/bmj.h5527

36. Roberts K, Cannon J, Atkinson D, Brown A, Maguire G, Remenyi B, et al. Echocardiographic Screening for Rheumatic Heart Disease in Indigenous Australian Children: A Cost–Utility Analysis. *J Am Heart Assoc* 2017;6(3):e004515.

37. Zachariah JP, Samnaliev M. Echo-based screening of rheumatic heart disease in children: a cost-effectiveness Markov model. *J Med Econ* 2015;18(6):410–9.

38. Musuku J, Engel ME, Musonda P, Lungu JC, Machila E, Schwaninger S, et al. Prevalence of rheumatic heart disease in Zambian school children. *BMC Cardiovasc Disord* [Internet] 2018;18(1):135. Available from: http://www.ncbi.nlm.nih.gov/pubmed/8406708

For peer review only

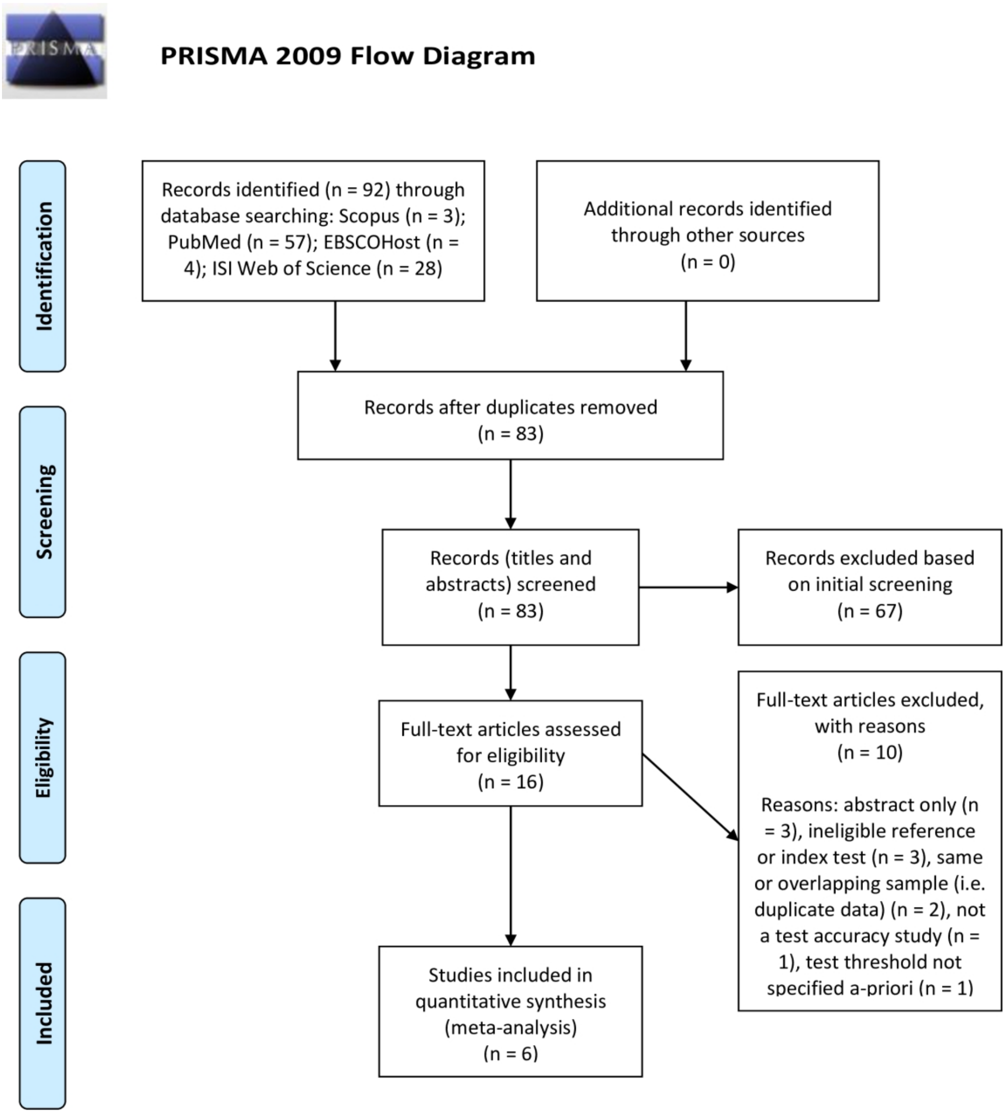

Figure 1. PRISMA flow diagram illustrating study identification, selection, eligibility and inclusion. From Moher et al.[17]

181x200mm (300 x 300 DPI)

Downloaded from http://bmjopen.bmj.com/ on February 13, 2018 - Published by group.bmj.com

Open Access Protocol

BMJ Open

Standard echocardiography versus handheld echocardiography for the detection of subclinical rheumatic heart disease: protocol for a systematic review

Lisa H Telford,[1,2] Leila H Abdullahi,[2,3,4] Eleanor A Ochodo,[5] Liesl J Zühlke,[1,2,6] Mark E Engel[1,4]

To cite: Telford LH, Abdullahi LH, Ochodo EA, *et al*. Standard echocardiography versus handheld echocardiography for the detection of subclinical rheumatic heart disease: protocol for a systematic review. *BMJ Open* 2018;**8**:e020140. doi:10.1136/bmjopen-2017-020140

► Prepublication history and additional material for this paper are available online. To view these files, please visit the journal online (http://dx.doi.org/10.1136/bmjopen-2017-020140).

Received 16 October 2017
Revised 4 December 2017
Accepted 21 December 2017

Check for updates

[1]Department of Medicine, Faculty of Health Sciences, University of Cape Town, Cape Town, South Africa
[2]Department of Paediatrics, Red Cross War Memorial Children's Hospital, University of Cape Town, Cape Town, South Africa
[3]Save the Children International (SCI), Somalia/Somaliland Country Office, Nairobi, Kenya
[4]Faculty of Health Sciences, University of Cape Town, Cape Town, South Africa
[5]Centre for Evidence-Based Health Care, Faculty of Medicine and Health Sciences, Stellenbosch University, Cape Town, South Africa
[6]Division of Cardiology, Groote Schuur Hospital, Cape Town, South Africa

Correspondence to
Professor Liesl J Zühlke;
liesl.zuhlke@uct.ac.za

ABSTRACT

Introduction Rheumatic heart disease (RHD) is a preventable and treatable chronic condition which persists in many developing countries largely affecting impoverished populations. Handheld echocardiography presents an opportunity to address the need for more cost-effective methods of diagnosing RHD in developing countries, where the disease continues to carry high rates of morbidity and mortality. Preliminary studies have demonstrated moderate sensitivity as well as high specificity and diagnostic odds for detecting RHD in asymptomatic patients. We describe a protocol for a systematic review on the diagnostic performance of handheld echocardiography compared to standard echocardiography using the 2012 World Heart Federation criteria for diagnosing subclinical RHD.

Methods and analysis Electronic databases including PubMed, Scopus, Web of Science and EBSCOhost as well as reference lists and citations of relevant articles will be searched from 2012 to date using a predefined strategy incorporating a combination of Medical Subject Heading terms and keywords. The methodological validity and quality of studies deemed eligible for inclusion will be assessed against review specific Quality Assessment of Diagnostic Accuracy Studies 2 criteria and information on metrics of diagnostic accuracy and demographics extracted. Forest plots of sensitivity and specificity as well as scatter plots in receiver operating characteristic (ROC) space will be used to investigate heterogeneity. If possible, a meta-analysis will be conducted to produce summary results of sensitivity and specificity using the Hierarchical Summary ROC method. In addition, a sensitivity analysis will be conducted to investigate the effect of studies with a high risk of bias.

Ethics and dissemination Ethics approval is not required for this systematic review of previously published literature. The planned review will provide a summary of the diagnostic accuracy of handheld echocardiography. Results may feed into evidence-based guidelines and should the findings of this review warrant a change in clinical practice, a summary report will be disseminated among leading clinicians and healthcare professionals in the field.

PROSPERO registration number CRD42016051261.

Strengths and limitations of this study

- ► We will evaluate the accuracy of handheld echocardiography for detecting subclinical rheumatic heart disease in endemic areas, making the proposed review relevant to current global agendas.
- ► We will not impose a search filter or any limits in terms of language during the literature search so as to minimise the chance of missing studies.
- ► Data extraction will be performed by two independent reviewers, thereby reducing the risk of bias.
- ► Accuracy measures (sensitivity and specificity) may be influenced by underestimated burden of disease estimates (incidence and prevalence) due to the scarcity of good quality epidemiological data.
- ► Variation in diagnostic criteria for handheld echocardiography may affect data synthesis.

INTRODUCTION

Background

Rheumatic heart disease (RHD) is a permanent heart valve condition resulting from an abnormal immune reaction to group A streptococcal infection typically occurring in childhood.[1] If left untreated, disease progression can result in irreversible heart valve damage, cardiac failure, stroke and premature death.[2 3] Significantly, RHD is a preventable and treatable chronic condition which mostly affects disadvantaged populations across the world.[2] Even though the disease has mostly been eradicated in North America and Europe, barring a few indigent pockets, it remains prolific in areas of the Middle East, the South Pacific, Africa as well as Central and South Asia.[2]

The continued persistence of RHD contributes to considerable amounts of preventable morbidity and mortality, particularly among adolescents and young adults.[4] This adds additional strain to what are often already overburdened health systems with endemic

For peer review only

For peer review only - http://bmjopen.bmj.com/site/about/guidelines.xhtml

Downloaded from http://bmjopen.bmj.com/ on February 13, 2018 - Published by group.bmj.com

Table 1 Search strategy

Database	Search terms	Limits
PubMed	((((((((((((((Hand-held) OR handheld) OR hand held) OR hand-carried) OR hand carried) OR HAND) OR HCU) OR HHCU) OR pocket size) OR pocket sized) OR portable) OR miniaturisation) OR miniaturised) OR focused) OR focus)) AND ((((‘Echocardiography’[Mesh]) OR echocardiography) OR echocardiographic) OR cardiac ultrasound)) AND (((‘Rheumatic Heart Disease’[Mesh]) OR rheumatic heart disease) OR RHD)<br>MeSH terms will be exploded during the search	Limited to 2012–2017
Scopus	1. Hand-held OR handheld OR hand held OR hand-carried OR hand carried OR HAND OR HCU OR HHCU OR pocket size* OR portable OR miniatur* OR focus*<br>2. Echocardiograph* OR cardiac ultrasound<br>3. Rheumatic Heart Disease OR RHD<br>#1 AND #2 AND #3	Limited to 2012–2017
ISI Web of Science	1. Hand-held OR handheld OR hand held OR hand-carried OR hand carried OR HAND OR HCU OR HHCU OR pocket size OR pocket sized OR portable OR Miniaturisation OR Miniaturised OR focused OR focus<br>2. Echocardiography OR Echocardiographic OR cardiac ultrasound<br>3. Rheumatic Heart Disease OR RHD<br>Combine #1 AND #2 AND #3	Limited to 2012–2017 and filtering out Medline
EBSCOHost	▶ S1. Hand-held OR handheld OR hand held OR hand-carried OR hand carried OR HAND OR HCU OR HHCU OR pocket size OR pocket sized OR portable OR Miniaturisation OR Miniaturised OR focused OR focus<br>▶ S2. Echocardiography OR Echocardiographic OR cardiac ultrasound<br>▶ S3. Rheumatic Heart Disease OR RHD<br>S1 AND S2 AND S3	Limited to 2012–2017

EBSCO, Elton B Stephens Company; HAND, handheld cardiac ultrasound; HCU, handheld cardiac ultrasound; HHCU, hand-held cardiac ultrasound; ISI, Institute for Scientific Information; MeSH, Medical Subject Heading terms; RHD, rheumatic heart disease.

regions, which are typically poorly resourced, bearing the brunt of the disease.[1 5] Furthermore, the accurate detection of subclinical RHD in children and adolescents remains hampered by the cost of diagnostic machinery and scarcity of trained personnel.[6] Alternative RHD screening tests, which are both accurate and affordable, are therefore needed in many endemic areas. The value of such a screening test is that significantly more cases of subclinical RHD might be detected, thereby reducing the time to commencement of secondary prophylaxis and thus, in turn, improving long-term outcomes.[7]

Recently, handheld echocardiography has become widely available with a variety of clinical uses.[8] Similarly, diagnostic accuracy has already been demonstrated in a number of studies assessing its value as a screening tool, despite some limitations such as lack of Doppler capabilities. Due to the non-invasive, safe, portable and relatively inexpensive nature of handheld echocardiography, the device has been presented in recent publications as a promising alternative to standard echocardiography in resource-limited and remote settings.[4 8] To test this assertion, the diagnostic accuracy of handheld echocardiography needs to be evaluated using a systematic approach. This review, therefore, proposes to evaluate the accuracy of handheld echocardiography for the detection of RHD in children and adolescents within a screening setting. We seek to generate new quantitative evidence for clinicians and guideline developers to establish evidence-based guidelines for diagnosing RHD with handheld echocardiography. Ultimately, this will improve the management of patients with RHD, as effective treatment of subclinical RHD requires accurate and timely diagnosis.

Primary objective

To determine the diagnostic accuracy of handheld echocardiography compared with standard echocardiography (two-dimensional (2D), continuous-wave and colour-Doppler echocardiography) performed by an experienced imager in conjunction with the 2012 World Heart Federation (WHF) criteria for the detection of any RHD in children and adolescents.

Secondary objective

To investigate potential sources of variation in relation to age, gender, geographical location, echocardiographic criteria and echocardiographer expertise in detecting subclinical RHD with handheld echocardiography.

METHODS AND ANALYSIS

The protocol was prepared according to the Preferred Reporting Items for Systematic Review and Meta-Analysis (PRISMA) guidelines. A PRISMA Protocol checklist is completed and included in online supplementary appendix 1.[9]

Inclusion and exclusion criteria

We will include all primary observational studies which compare the diagnostic accuracy of handheld

For peer review only - http://bmjopen.bmj.com/site/about/guidelines.xhtml

Downloaded from http://bmjopen.bmj.com/ on February 13, 2018 - Published by group.bmj.com

Table 2 Design-specific criteria to assess methodological quality

	Domains			
**Categories**	**1. Patient selection**	**2. IT**	**3. RS**	**4. Flow and timing**
Description	Briefly describe the methods of patient selection:	Describe the IT (HAND), how it was conducted and interpreted:	Describe the RS (STAND) how it was conducted and interpreted:	Describe patients that did not receive HAND, and/or STAND or who were excluded from the 2×2 table.<br>Describe the time interval and any interventions between the HAND and STAND.
Indicator questions (yes, no, unclear)	Was a consecutive or random sample of patients enrolled?	Were the HAND results interpreted without knowledge of the results of STAND?	Was STAND likely to correctly classify the target condition?	Was there an appropriate time interval between HAND and STAND?
	Was a case–control design avoided?	Was a prespecified threshold used?	Were the STAND results interpreted without knowledge of the HAND results?	Did all patients receive STAND and was it the same RS?
	Did the study avoid inappropriate exclusions?			Were all patients included in the analysis?
*Risk of bias (low, high, unclear)	Based on the indicator questions, could the selection of patients have introduced bias?	Based on the indicator questions, could the conduct or interpretation of HAND have introduced bias?	Based on the indicator questions, could STAND, its conduct or its interpretation have introduced bias?	Based on the indicator questions, could the patient flow and timing have introduced bias?
Concerns Regarding Applicability (low, high, unclear)	Describe included patients (prior testing, presentation, intended use of HAND and setting):<br>Based on the description of included patients, are there concerns that the included patients do not match the review question?	Are there concerns that HAND, its conduct, or interpretation differ from the review question?	Are there concerns that the target condition as defined by STAND does not match the review question?	

*Criteria for grading risk of bias: If all indicator questions for a single domain are answered ‘ yes’, then the risk of bias will be judged as being ‘ low’; if any indicator question is answered ‘ no’, then the potential for bias will be flagged and the review authors will be required to judge the risk of bias with the assistance of the senior author (MEE); if all or most indicator questions were answered ‘ no’, then the risk of bias will be judged as being ‘ high’ and indicator questions are can only be answered as ‘unclear’ when the data are insufficient to allow for the formulation of a judgement.
Adapted from Whiting *et al*.[11]
IT, index test; RS, reference standard.

echocardiography to the reference standard; standard echocardiography performed by an experienced imager and in conjunction with the 2012 WHF criteria. Eligible studies can be of a cross-sectional, cohort or diagnostic case–control design, provided both cases and controls have been sampled from the same population. Studies which report on, or contain the data necessary to extract information on the proportions of true positives (TP), false positives (FP), true negatives (TN) and false negatives (FN) will be included. Studies which enrolled only those with a confirmed RHD diagnosis will be excluded on account of the potential for overestimation of sensitivity. Descriptive studies such as case studies/series will also be excluded from this review. Studies in which we are unable to generate two-by-two tables, as well as different studies which report on duplicate data will not be considered for inclusion in this review.

We will consider all studies in which samples of study participants are either, a randomly, or consecutively selected series of individuals from populations in which RHD is prevalent worldwide for inclusion. For the purposes of this review, children and adolescents will be defined as being between the ages of 5 and 17 years (age range: ≥5 years to <18 years). More specifically, participants will be considered children if they are between 5 and 9 years of age and adolescents if they are between 10 and 17 years of age.

We will include studies evaluating the accuracy of handheld echocardiography for RHD detection. There will be no restrictions regarding the type of handheld device used or the aptitude of person performing the cardiac ultrasound; however, these data will be recorded and analysed accordingly. Studies will be deemed eligible for inclusion if the reference standard constituted the

For peer review only - http://bmjopen.bmj.com/site/about/guidelines.xhtml

Downloaded from http://bmjopen.bmj.com/ on February 13, 2018 - Published by group.bmj.com

interpretation of echocardiographic findings using the 2012 WHF criteria when echocardiographic assessment by 2D, continuous-wave and colour-Doppler echocardiography was performed by a cardiologist or cardiac sonographer. We will exclude all studies published before 2012 to omit any study which does not use standard echocardiography in conjunction with the 2012 WHF criteria as the reference standard. We will consider all studies which evaluate any RHD (definite and borderline) as the condition of interest for inclusion in this review. All case definitions will be consistent with the 2012 WHF criteria.[10]

Search strategy

A comprehensive electronic literature search of PubMed, Scopus, Web of Science and EBSCOhost will be conducted to identify relevant literature. No restrictions in terms of language will be applied during the search. Searches will however be limited to only include articles published from 2012 up until the present. All sources will be systematically searched using a combination, where relevant, of both free text words and Medical Subject Heading terms. Search strategies will be tailored to meet the requirements of each electronic database as in table 1 below. Search terms will include synonyms for 'rheumatic heart disease', 'echocardiography' and 'handheld'. A list of all articles identified through the literature search will be compiled and references managed using Mendeley software. In addition, a manual search of all eligible articles' reference lists, articles citing eligible articles as well as relevant review articles will be carried out to identify any additional literature not identified by the comprehensive electronic literature search. Abstracts from any relevant conference proceedings will also be searched for among appropriate websites and followed up on if eligibility requirements are sufficiently met. Finally, experts in the field will be contacted for additional information where necessary.

Selection of studies for inclusion

The titles and/or abstracts of all articles identified by the literature search will be screened independently by two reviewers. Based on the predefined inclusion and exclusion criteria any clearly ineligible studies will be excluded. Following this, the full-text versions of all potentially eligible studies will then be reviewed by two independent reviewers to assess their eligibility. Any discrepancies regarding eligibility will be resolved through discussion and consensus with a third reviewer.

Data extraction and management

Using a predefined data extraction form, two reviewers will independently extract the following information from all studies meeting the criteria for inclusion:

- Study identifiers: author(s), year of publication, journal;
- Study characteristics: study design, study country/setting/context, study population/participants, sample size, participant recruitment procedures, participant demographics and RHD prevalence (pretest probability);
- Reference standard and index test details:
  - General: test positive or negative;
  - Specific: individual findings on cardiac ultrasound;
  - Expertise of person(s) performing and/or interpreting tests: expert versus non-expert;
  - Diagnostic criteria: test threshold(s);
  - Number of missing or unavailable test results.
- Diagnostic test outcome measures: sensitivity, specificity, positive and negative predictive values, number of TP, FP, TN and FN.

If necessary, any disagreements will be resolved through discussion with a third reviewer until a consensus is reached. Any data missing from the reports of included studies will be requested from study authors. In cases where studies have used different diagnostic criteria for handheld echocardiography, attempts will be made to standardise them to mirror the 2012 WHF criteria as closely as possible. The information garnered through the data extraction process will be used to determine each study's quality as well as for synthesising evidence.

Risk of bias and quality assessment

The Quality Assessment of Diagnostic Accuracy Studies (QUADAS-2) tool (see table 2) will be used to assess the risk of bias and concerns regarding applicability of all included studies.[11] The tool encompasses four domains which have been tailored to meet the specific requirements of the review. Two reviewers will independently assess the risk of bias in all included studies according to the revised QUADAS-2 criteria. Any discrepancies will be resolved through discussion until consensus is reached and with the assistance of a third reviewer if necessary. Both text and graphics will be used to demonstrate the results.

Subgroup and sensitivity analyses

Subgroup analysis may be performed, considering specific characteristics of the studies, such as echocardiography protocol, training background of the examiner, age and geographical location.

We will conduct a sensitivity analysis to investigate the effect of variations in criteria on the overall accuracy of diagnosis. In addition, we will explore the effect of excluding studies with a high risk of bias on the accuracy of summary estimates, sensitivity and specificity. We will not investigate publication bias.

Statistical analysis and data synthesis

We will first analyse data descriptively by plotting the sensitivity and specificity (including 95% CIs) of all included studies in both forest plots and receiver operating characteristic (ROC) space. These plots will be generated using the Review Manager software package.[12] If there are sufficient data, we will conduct a meta-analysis to produce summary results of sensitivity and specificity. Because we anticipate that studies will have different

For peer review only - http://bmjopen.bmj.com/site/about/guidelines.xhtml

Downloaded from http://bmjopen.bmj.com/ on February 13, 2018 - Published by group.bmj.com

positivity thresholds due to the use of different sets of diagnostic criteria, we will pool the results using the hierarchical summary receiver operating characteristic (HSROC) method. Meta-analysis will be performed using SAS V.9.4/STATA V.14.2 software.[13] We will also explore, through metaregression, the relationship of test accuracy with categorical or continuous covariates such as test threshold.[14]

Investigations of heterogeneity will initially begin by visually examining the forest and ROC plots for heterogeneity in sensitivity and specificity. We will then analyse the possible sources of heterogeneity as covariates in the statistical models. Potential sources of heterogeneity to be investigated as categorical variables include; age (children vs adolescents), sex (male vs female), geographical location (high vs low/middle-income countries), diagnostic criteria (single vs multiple views and different thresholds) and echocardiographer expertise (expert vs non-expert).

Presenting and reporting of results

The study selection process will be summarised in the form of a flow diagram detailing the reasoning behind all exclusions. Results will be reported in accordance with the PRISMA guidelines.[15]

Dissemination

The planned review will provide a summary of the diagnostic accuracy of handheld echocardiography. Results may feed into evidence-based guidelines and will therefore be disseminated to members of the WHF criteria working group. Should the findings of this review warrant a change in clinical practice, a summary report will be circulated among leading clinicians and healthcare professionals in the field.

Contributors LJZ and MEE conceived the study idea and all the authors contributed to the conception and design of the protocol. LHT developed and wrote the first draft of the protocol. All authors have reviewed and accepted the final version of the protocol and have given their permission for publication. All authors contributed to editing subsequent versions of the draft. LHT and LHA will perform the literature searches as well as extract data and LHT and EAO will conduct the data analysis. All authors are in agreement to be accountable for all aspects of the work in ensuring that questions related to the accuracy or integrity of any part of the work are appropriately investigated and resolved.

Funding LJZ and LHT receive funding from the Medtronic Foundation through support to RHD Action. LJZ and LT receive funding from the National Research Foundation of South Africa (NRFSA)

Competing interests None declared.

Patient consent Not required.

Provenance and peer review Not commissioned; externally peer reviewed.

Open Access This is an Open Access article distributed in accordance with the Creative Commons Attribution Non Commercial (CC BY-NC 4.0) license, which permits others to distribute, remix, adapt, build upon this work non-commercially, and license their derivative works on different terms, provided the original work is properly cited and the use is non-commercial. See: http://creativecommons.org/licenses/by-nc/4.0/

© Article author(s) (or their employer(s) unless otherwise stated in the text of the article) 2018. All rights reserved. No commercial use is permitted unless otherwise expressly granted.

REFERENCES

1. Marijon E, Mirabel M, Celermajer DS, *et al*. Rheumatic heart disease. *Lancet* 2012;379:953–64.
2. World Heart Federation. *Rheumatic Heart Disease (RHD): Neglected NCD of Poverty*. Geneva: World Heart Federation, 2013.
3. Seckeler MD, Hoke TR. The worldwide epidemiology of acute rheumatic fever and rheumatic heart disease. *Clin Epidemiol* 2011;3:67–84.
4. Godown J, Lu JC, Beaton A, *et al*. Handheld echocardiography versus auscultation for detection of rheumatic heart disease. *Pediatrics* 2015;135:e939–e944.
5. Zühlke LJ, Steer AC. Estimates of the global burden of rheumatic heart disease. *Glob Heart* 2013;8:189–95.
6. Lu JC, Sable C, Ensing GJ, *et al*. Simplified rheumatic heart disease screening criteria for handheld echocardiography. *J Am Soc Echocardiogr* 2015;28:463–9.
7. Dougherty S, Khorsandi M, Herbst P. Rheumatic heart disease screening: Current concepts and challenges. *Ann Pediatr Cardiol* 2017;10:39.
8. Saxena A. Rheumatic heart disease screening by "point-of-care" echocardiography: an acceptable alternative in resource limited settings? *Transl Pediatr* 2015;4:210–3.
9. Moher D, Shamseer L, Clarke M, *et al*. Preferred reporting items for systematic review and meta-analysis protocols (PRISMA-P) 2015 statement. *Syst Rev* 2015;4:1):1.
10. Reményi B, Wilson N, Steer A, *et al*. World heart federation criteria for echocardiographic diagnosis of rheumatic heart disease—an evidence-based guideline. *Nat Rev Cardiol* 2012;9:297–309.
11. Whiting PF, Rutjes AW, Westwood ME, *et al*. QUADAS-2: a revised tool for the quality assessment of diagnostic accuracy studies. *Ann Intern Med* 2011;155:529–36.
12. Review Manager (RevMan). *Copenhagen: the nordic cochrane centre, the cochrane collaboration*, 2014.
13. SAS/STAT Software. *SAS Institute Inc*, 2011.
14. Macaskill P, Gatsonis C, Deeks JJ, *et al*. Analysing and presenting results. In: Deeks JJ, Bossuyt PM, Gatsonis C, eds. *Cochrane handbook for systematic reviews of diagnostic test accuracy version 10 the cochrane collaboration*, 2010.
15. Moher D, Liberati A, Tetzlaff J, *et al*. Academia and clinic annals of internal medicine preferred reporting items for systematic reviews and meta-analyses: The PRISMA Statement. *Annu Intern Med* 2009;151:264–9.

For peer review only - http://bmjopen.bmj.com/site/about/guidelines.xhtml

Downloaded from http://bmjopen.bmj.com/ on February 13, 2018 - Published by group.bmj.com

BMJ Open

Standard echocardiography versus handheld echocardiography for the detection of subclinical rheumatic heart disease: protocol for a systematic review

Lisa H Telford, Leila H Abdullahi, Eleanor A Ochodo, Liesl J Zühlke and Mark E Engel

*BMJ Open*2018 8:
doi: 10.1136/bmjopen-2017-020140

Updated information and services can be found at:
http://bmjopen.bmj.com/content/8/2/e020140

These include:

References This article cites 11 articles, 1 of which you can access for free at:
http://bmjopen.bmj.com/content/8/2/e020140#ref-list-1

Open Access This is an Open Access article distributed in accordance with the Creative Commons Attribution Non Commercial (CC BY-NC 4.0) license, which permits others to distribute, remix, adapt, build upon this work non-commercially, and license their derivative works on different terms, provided the original work is properly cited and the use is non-commercial. See: http://creativecommons.org/licenses/by-nc/4.0/

Email alerting service Receive free email alerts when new articles cite this article. Sign up in the box at the top right corner of the online article.

Topic Collections Articles on similar topics can be found in the following collections

Diagnostics (220)
Screening (epidemiology) (24)

Notes

To request permissions go to:
http://group.bmj.com/group/rights-licensing/permissions

To order reprints go to:
http://journals.bmj.com/cgi/reprintform

To subscribe to BMJ go to:
http://group.bmj.com/subscribe/

For peer review only

Data Extraction Form

Reviewer Name:		Date Form Completed: (dd/mm/yyyy)	

1. General Information

Publication Type: (e.g. full report, abstract)			
Journal:			
Article Title:			
First Author:		Publication Year:	
Author Contact Details:			
References of Potentially Eligible Studies: (from the bibliography hand search)			

**1a.** Is this a study that examines the accuracy of tests for rheumatic heart disease (RHD)?	☐ Yes	
	☐ No	**Exclude paper!**
	☐ Unclear	Comment:
**1b.** If 'No' at item 1a., please describe the type of article		

2. Target Condition

Page(s):

**2.** Rheumatic heart disease (RHD)	☐ Definite<br>☐ Borderline<br>☐ Mixed definite & borderline	
	☐ No RHD in study	**Exclude paper!**
	Comment:	

3. Study Population

Page(s):

**3a.** Country of study	Please state the country or countries in which the study took place:		
**3b.** What is the estimated prevalence of RHD in the study area prior to the study?	Prevalence / Pre-test probability:		☐ Not reported
**3c.** Age of participants	Mean/Median Age:	SD/Range:	☐ Not reported
**3d.** Total number of participants	No. of participants:		

For peer review only

included in study (sample size)	No. of samples:		☐ Not reported
**3e**. Proportion or number of participants by sex	Male:	Female:	☐ Sex not specified

FOR THE QUESTIONS 4 & 5, DRAWING A FLOW DIAGRAM OF THE STUDY MAY BE HELPFUL
(space is provided on page 6 of this data extraction form)

**4. Patient Selection**	**Page(s):**		
**4a.** Please cite the patient selection criteria	Inclusion Criteria:	Exclusion Criteria:	
	☐ Not reported	☐ Unclear<br>Comment:	
**4b.** What was the stage of disease at enrolment?	☐ Asymptomatic<br>☐ Symptomatic<br>☐ Both asymptomatic & symptomatic<br>☐ Unclear<br>Comment:		
**4c.** What was the study design?	☐ Cross-sectional study<br>☐ Cohort study<br>☐ Case-control (cases & controls from the same population/nested)<br>☐ Case-control (with healthy controls) EXCLUDE!<br>☐ Case-control (with alternative diagnosis controls) EXCLUDE!<br>☐ Case-control (with controls from non-endemic areas) EXCLUDE!<br>☐ Randomized trial<br>☐ Not stated<br>☐ Unclear<br>Comment:		
**4d.** Was a case-control design avoided?	☐ Yes	☐ No	☐ Unclear
**4e.** Was either a consecutive or random sample of patients enrolled? (please specify which)	☐ Yes	☐ No	☐ Unclear
	Comment:		
**4f.** Did the study avoid inappropriate exclusions?	☐ Yes	☐ No	☐ Unclear

**4g.** Could the selection of patients have introduced bias? (Please indicate the level of risk)	☐ Low risk of bias (if questions 4d and 4e were both answered 'yes')<br>☐ High risk of bias (if both question 4d and 4e were answered 'no')<br>☐ Unclear (any other combination of answers)
**4h.** Is there concern that the included patients do not match the review question? (Please indicate the level of concern)	☐ Low concern (participants from endemic areas)<br>☐ High concern (tourists, non endemic areas)<br>☐ Unclear concern<br>Comment:
**5. Patient Flow and Timing**	**Page(s):**
**5a.** Was there an appropriate interval between the index test and reference standard?	☐ Yes ☐ No ☐ Unclear
**5b.** Did all patients receive a reference standard test? (Focus on 2*2 table)	☐ Yes ☐ No ☐ Unclear
**5c.** Did all patients receive the same reference standard test?	☐ Yes ☐ No ☐ Unclear
**5d.** Were all patients included in the analysis?	☐ Yes ☐ No ☐ Unclear
**5e.** Could the patient flow have introduced bias? (Please indicate the level of risk of bias)	☐ Low (if questions 5a-d were all answered 'yes', or at least three were answered 'yes' and the other 'unclear')<br>☐ High (if two or more of questions 5a-d were answered 'no')<br>☐ Unclear (any other combination of answers)

**6. Index Test**	**Page(s):**		
**6a.** Please state index test's name (i.e. brand name/manufacturer) and the person(s) performing and interpreting the test as well as the time at which interpretation occurred (i.e. on-site/at time of screening or later/offline)	Test name:		☐ Not stated
	Performed by:		
	Interpreted by:		
	Time of interpretation:		
**6b.** Was a pre-specified set of diagnostic criteria or protocol used?	☐ Yes ☐ No ☐ Unclear ☐ Not stated		Comment:
**6c.** Was the HAND interpreter trained to use the protocol? If the HAND interpreter has prior experience/skill/expertise and training was not necessary please also select 'Yes'.	☐ Yes ☐ No ☐ Unclear ☐ Not stated		Comment:
**6d.** What was the initial level of expertise of the HAND interpreter?		☐ Not stated	Comment:

w only

**6e.** Was quality control done? (e.g. 10% of samples cross checked by another person)	☐ Yes ☐ No ☐ Unclear ☐ Not stated		Comment:
**6f.** Were the index test results interpreted without knowledge of the results of the reference standard?	☐ Yes ☐ No ☐ Unclear ☐ Not stated		Comment:
**6g.** If a diagnostic threshold was used was it pre-specified?	☐ Yes ☐ No ☐ Unclear ☐ Not stated		Comment:
**6h.** Could the conduct or interpretation of the index test have introduced bias?	☐ Low risk of bias (if questions 6b-g were all answered 'yes')<br>☐ High risk of bias (if two or more of questions 6b-g were answered 'no')<br>☐ Unclear (any other combination of answers)		

**7. Reference Test**	**Page(s):**		
**7a.** Please state reference test's name (i.e. brand name / manufacturer) and the person(s) performing and interpreting the test as well as the time at which interpretation occurred (i.e. on-site / at time of screening or later / offline)	☐ 2D, continuous-wave, & colour-Doppler echocardiography	☐ Other	**Exclude paper!**
	Test Name:		
	Performed by:		
	Interpreted by:		
	Time of interpretation:		
**7b.** Number of reference test images examined per patient		☐ Unclear	☐ Not stated
**7c.** Was quality control done? (e.g. were 10% of samples cross checked by another person)	☐ Yes ☐ No ☐ Unclear ☐ Not stated		Comment:
**7d.** Is the reference standard likely to classify the target condition?	☐ Yes ☐ No ☐ Unclear		Comment:
**7e.** Were the reference standard results interpreted without knowledge of the results of the index test?	☐ Yes ☐ No ☐ Unclear ☐ Not stated		Comment:
**7f.** Could the reference standard, its conduct, or its interpretation have introduced bias?	☐ Low risk of bias (if questions 7c-e were all answered 'yes')<br>☐ High risk of bias (if one or more of questions 7c-e were answered 'no')<br>☐ Unclear (any other combination of answers)		

**8. Test Results**	**Page(s):**
**8a. Prevalence of disease based on STAND results:** Please state mean and range/confidence intervals *(for all who were*	Mean:
	Range:
	CI:

screened with STAND irrespective of HAND)	☐ Not stated	Comment:
**8b. Severity of disease:** Please state the disease severity according to assessment by STAND *(only for those who received both HAND & STAND)*		☐ Not stated
**8c. Missing Data:** Were there missing, uninterpretable or unavailable results of the reference standard?	☐ Yes ☐ Not that I noticed<br>Please explain:	
Were there missing, uninterpretable or unavailable results of the index test?	☐ Yes ☐ Not that I noticed<br>Please explain:	

8d. Accuracy Results

- Please calculate and list results of the *index test* below: TP,TN, FP, FN, Sens, Spec.
- Please also indicate the *main test threshold* used to calculate the results.
- If *multiple thresholds* have been used please indicate so in the comments box.

Table A

Test	Test Threshold	TP	FP	FN	TN	Sens	Spec

Comments:

Flow Diagram

For questions **4** and **5**, drawing a flow diagram of the study may be helpful. Flow diagrams of patient selection, flow and timing illustrate the number of patients who were eligible for the study, how

many were actually recruited, the proportion of which received the index test as well as the proportion of which received the reference standard etc. In addition, the number of true and false positives and true and false negatives are also displayed. If necessary please draw a flow diagram for the primary study to aid your understanding**.**

For peer review only

Background Document to the Data Extraction Form

***NB:** At the beginning of each section please write the page number(s) on which the majority of the information pertaining to that section was found. A space is provided next to the section heading for this.

(1) GENERAL INFORMATION

Publication type: Describe the type of publication, i.e.: full report, abstract, conference proceeding etc.

Journal: Name of the journal in which the article is published (if published)

Article title: Enter the title of the article

First author: Enter the full name of first author

Publication year: Enter the year in which the article was published

Author contact details: Record the contact details of the corresponding author for future reference in instances where additional data needs to be obtained or questions clarified

The following questions have activated checkboxes next to the responses. Please make your selection by clicking the appropriate checkbox.

1a. Is this a primary study that examines the accuracy of tests for rheumatic heart disease (RHD)?
Tick the appropriate box. If answered 'no', EXCLUDE the paper. This item is meant to enable the quick distinction between potentially relevant articles and articles that clearly have a different scope. If the article does not clearly evaluate the accuracy of tests as a primary or secondary objective you can tick 'no' and stop the data extraction exercise. If you are unsure please tick 'unclear', comment on why you are unsure and then proceed with the data extraction exercise.

1b. If the article does not evaluate accuracy tests for RHD, please describe what kind of article it is?
Please describe what type of publication it is (e.g. narrative overview article, a purely prevalence study etc.)

(2) TARGET CONDITION BEING EVALUATED

2. Please indicate the target condition being evaluated in the study.
[All studies should evaluate both borderline and definite in accordance with the 2012 WHF criteria]

For peer review only

Please tick the appropriate box to indicate if the study evaluates either definite or borderline RHD or both as the condition of interest. If it doesn't evaluate definite or borderline RHD but rather another condition; EXCLUDE the paper and add an explanation in the comment in the box provided.

(3) STUDY POPULATION

3a. Country of study

Some studies may be carried out in one country or in multiple countries. Please state the country or countries in which the study was conducted.

3b. What is the estimated prevalence of RHD in the study area prior to the study?

Prior to the study commencing, the authors may state the estimated prevalence of RHD in the area or country of study. This estimated prevalence may be based on previous reports or other studies. If it is not reported please tick the box 'Not reported'.

3c. Age of participants

Please indicate the mean or median and SD or range of the age of the study participants included in the study. Please state the measures that are reported in the study. If no measures were reported in the study, please tick the box 'Not reported'. Some studies may describe a subset of the larger cohort as receiving both the index test and reference standard and the entire cohort separately – in this instance please only report the age of participants who received both tests.

3d. Number of participants

Please write in the box provided the number of participants and/or the number of samples included in the study. Some studies may describe a subset of the larger cohort who received both the index test and reference standard and the entire cohort separately – in this instance please report only the number of participants who received both tests (i.e. the subset of participants who received both HAND and STAND). You may also state that the study consisted of a large cohort with a subset within that cohort in the space provided for 'number of samples'.

3e. Proportion or number of participants by gender

Please write in the box provided the proportion/percentage or number of participants per sex of those who received both tests (i.e. those that received both HAND and STAND). For example; 50% of participants were female or 590 females were in the study. This information can usually be found in the results section but if the sex of participants was not specified please tick the box 'Sex not specified'.

For peer review only

Flow diagram

For questions **4** and **5**, drawing a flow diagram of the study may be helpful. Flow diagrams of participant selection, flow and timing illustrate the number of participants who were eligible for the study, how many were actually recruited, the proportion of which received the index test as well as the proportion of which received the reference standard etc. In addition, the number of true and false positives and true and false negatives are also displayed. If necessary please draw a flow diagram for the primary study in the space provided on page 6 of the data extraction form.

An example of a flow diagram is as shown below.

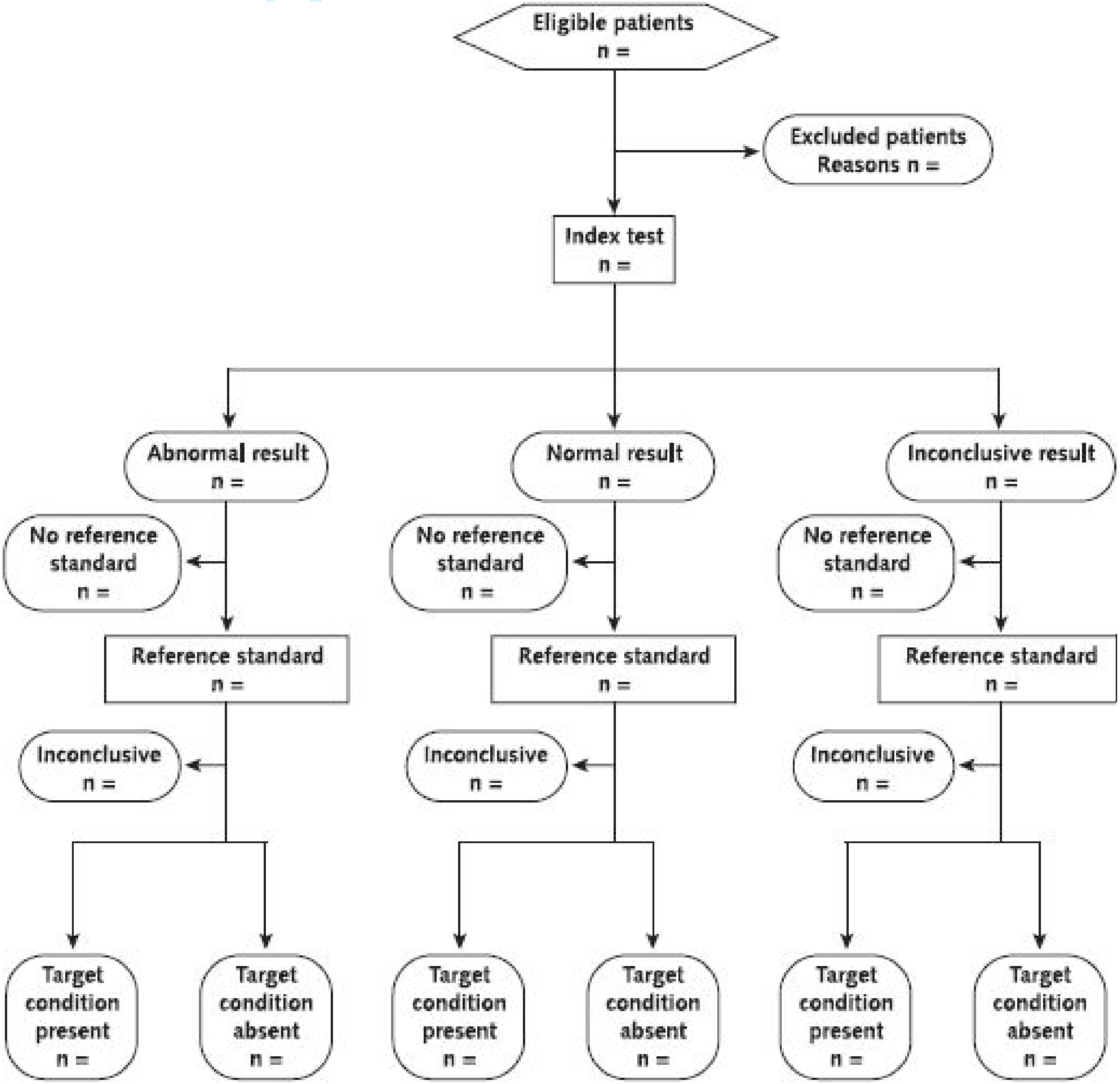

(4) PARTICIPANT SELECTION

These questions have been designed to help assess the risks of bias in the study.

4a. Please cite here the selection criteria

Please list the inclusion and exclusion criteria which were applied when recruiting study participants in the spaces provided. Inclusion criteria might also include the characteristics of included participants. For example "all children attending primary schools in the area were eligible for inclusion". If no criteria were reported, please tick 'Not reported' and if the criteria were unclear please tick "Unclear" and explain your answer.

4b. Stage of disease

Participants recruited into the study may be with or without symptoms. Please indicate the disease stage of participants at the time of enrolment by ticking the appropriate box. If the study does not clearly report the clinical status of participants, please tick the box marked 'unclear' and comment in the space provided.

4c. What was the study design?

Please indicate the design of the study by ticking one of the choices provided.

We will not include case-control studies which include healthy controls, alternative diagnosis controls or controls from non-endemic areas. Research has shown that these types of studies have a tendency to overestimate accuracy measures. Healthy controls are those who have been confirmed as being disease-free, alternative diagnosis controls are controls that have similar symptoms to those of the disease under study but do not have the condition of interest and controls from non-endemic areas are those from areas in which the condition of interest is not highly prevalent. If the study design is not reported or is unclear please tick the appropriate box and if necessary add a comment.

4d. Was a case-control design avoided?

- **Yes:** If the authors report using any study design apart from a case-control one.
- **No:** If the authors report using a case-control study design.
- **Unclear:** If the authors do not explicitly report the study design used.

4e. Was a consecutive or random sample of participants enrolled?

- **Yes:** If the authors report random sampling or consecutive enrolment of participants.
- **No:** If participants were selected, for example based on previous (reference or index) test results.

For peer review only

- **Unclear:** There appears to be no problem, but the authors do not explicitly state that participants were enrolled randomly or consecutively.

4f. Did the study avoid inappropriate exclusions?

- **Yes:** If no participants were excluded after inclusion/enrolment.
- **No:** If, for example, participants with mild disease severity were excluded, because they are more difficult to detect.
- **Unclear:** If not reported or insufficient information given to make a decision.

4g. Could the selection of participants have introduced bias?

- **Low:** If both questions 4d and 4e were answered 'yes' or if at most one was answered 'unclear'.
- **High:** If one or more of questions 4d and 4e were answered 'no'.
- **Unclear:** Any other combination of answers, for example if both questions 4d and 4e were answered 'unclear'.

4h. Is there a concern that the included participants do not match the review question?

- **Low concern:** If study participants reside within RHD endemic areas as they will include those at risk of infection, those who are infected but asymptomatic as well as those who are infected and have symptoms.
- **High concern:** If study participants don't reside in endemic areas. For example; tourists, healthy controls or controls with alternative diagnoses.
- **Unclear:** If there is insufficient information to make a decision.

(5) PARTICIPANT FLOW AND TIMING

5a. Was there an appropriate interval between index test and reference standard?

- **Yes:** If the participants were examined using both the reference standard and index test at the same time or within a two week time period.
- **No:** If the time period between index and reference standard was more than two weeks.
- **Unclear:** If there is no or insufficient information on time period.

5b. Did all participants receive a reference standard?

(Focus on participants included in the 2*2 table)

- **Yes:** If the whole study sample or a random selection of the sample or a selection of the sample with consecutive series receive verification using the reference standard.

For peer review only

- **No:** If a part of the study sample that is non-randomly or non-consecutively selected receives verification using the reference standard.
- **Unclear:** If there is no or insufficient information to ascertain whether the whole sample or a random selection of the sample received verification using the reference standard.

5c. Did participants receive the same reference standard?

- **Yes:** If study participants are tested with the same reference standard (2D, continuous-wave, and colour Doppler echocardiography) regardless of index test result.
- **No:** If 2D, continuous-wave, and colour Doppler echocardiography is used with different techniques depending on the results of the index test.
- **Unclear:** If there is no or insufficient information on the reference standard used.

5d. Were all participants included in the analysis?

- **Yes:** If all the participants that were included in the study, were also included in the analysis.
- **No:** If some participants / results are missing in the analysis.
- **Unclear:** If there is no or insufficient information to make a decision.

5e. Could the conduct or interpretation of participant flow & timing have introduced bias?

- **Low:** If all questions were answered 'yes' or at least three were answered 'yes' and the other 'unclear'.
- **High:** If two or more of questions 5a - 5d were answered 'no'.
- **Unclear:** Any other combination of answers. For example if all questions were answered 'unclear' or if three were 'unclear' and one was 'yes'.

(6) INDEX TEST

6a. Please state the name and specifications of the index test under evaluation in the study.
Please state the name/label of the index test as well as any additional specifications. The label is the brand name of the test or the manufacturer who made the device. Additional specifications might include the type of transducer/probe attached to the device. If the brand name or label is not reported please tick the box 'Not reported'. Please also describe the person(s) performing and interpreting the test as well as the time at which interpretation occurred. In some instances the person(s) performing the index test might be different from the person(s) interpreting the test results – please describe both. Please also describe the time at which the index test results were interpreted, for example on-site at the time of screening/evaluation or later/offline.

For peer review only

6b. Was a pre-specified set of diagnostic criteria or protocol used?

- **Yes:** If a pre-specified modified diagnostic protocol or set of diagnostic criteria for HAND was used.
- **No:** If no pre-specified set of diagnostic criteria or diagnostic protocol was used or if criteria were not modified to suit the capabilities of HAND.
- **Unclear:** If there is insufficient information to make a judgment.
- **Not reported:** If there is no information reported on this item.

6c. Was the HAND interpreter trained to use the diagnostic protocol?

- **Yes:** If the person who conducted the echocardiographic screening using the handheld device was trained on how to interpret results using the pre-specified diagnostic protocol or if the person interpreting the HAND results is described as an expert/experienced reviewer/imager/reader. For example this might include any of the following; paediatric cardiologists, paediatric cardiology fellows, echocardiography imagers/technicians & sonographers.
- **No:** If the person who conducted echocardiographic screening using the handheld device does not have any prior experience or expertise in interpreting echocardiographic images and was not given any training with regards to interpreting results using a pre-specified diagnostic protocol.
- **Unclear:** If there is insufficient information to make a judgment.
- **Not reported:** If there is no information reported on this item.

6d. What was the initial level of expertise of the HAND interpreter?

Please state the HAND interpreter's initial level of clinical expertise. For example were they a clinician, nurse or community health worker etc. If not reported please tick the appropriate box and if necessary comment in the space provided.

6e. Was quality control done?

To ensure reliability or good quality of results a sub-set of the sample population may be cross-checked by a second person or an expert in echocardiographic imaging. Please indicate if this was done in the study. If the information is insufficient or if the information given is unclear please tick the box 'unclear'. If not reported, tick the box 'Not reported' and if necessary, comment in the box provided.

For peer review only

6f. Were the index test results interpreted without knowledge of the results of the reference standard?

- **Yes:** If results of the index test are interpreted without knowledge of reference test results or if the index test is performed prior to administering the reference standard.
- **No:** If results of the index test are interpreted with knowledge of the reference standard test results.
- **Unclear:** If there is insufficient information on when the index and reference tests were interpreted.
- **Not reported:** If no information was reported with regards to the timing and interpretation of tests.

6g. If a threshold was used, was it pre-specified?

- **Yes:** If the authors report the use of a primary, pre-specified, cut-off value or threshold. A pre-specified threshold also includes statements such as "the test was scored according to manufacturer's instructions".
- **No:** If multiple cut-off values were tested and the best one chosen afterwards.
- **Unclear:** If only one cut-off value was used, but this was not explicitly reported in the methods section.
- **Not reported:** If no information was reported on this item.

6h. Could the conduct or interpretation of the index test have introduced bias?

- **Low:** If questions 6b – 6g were all answered 'yes'.
- **High:** If one or more of questions 6b – 6g were answered 'no'.
- **Unclear:** Any other combination of answers. For example if one or more questions were answered 'unclear'.

(7) REFERENCE TEST

The reference test for RHD that this review will evaluate is 2D continuous-wave, and colour Doppler echocardiography with results interpreted in accordance with the 2012 WHF criteria.

7a. Please indicate the reference test applied in the study

For RHD, if the reference test used was not 2D, continuous-wave, and colour Doppler echocardiography tick 'other' and EXCLUDE the paper.

Please state the name/label of the reference standard machine as well as any additional specifications. The label is the brand name of the test or the manufacturer who made the device.

For peer review only

Additional specifications might include the type of transducer/probe attached to the machine. If the brand name or label is not reported please tick the box 'Not reported'. Please also describe the person(s) performing and interpreting the test as well as the time at which interpretation occurred. In some instances the person(s) performing the index test might be different from the person(s) interpreting the test results – please describe both. Please also describe the time at which the index test results were interpreted, for example on-site at the time of screening/evaluation or later/offline.

7b. Number of reference test images examined per participant

Please state the number of images recorded and examined per participant using 2D, continuous-wave, and colour Doppler echocardiography. If a different number of images were examined per participant or if the information is insufficient please tick the box marked 'unclear'. If no information is reported please tick the box 'Not reported'.

The following questions (7c-7f) are part of the QUADAS-2 tool and will be used to assess the risk of bias in how the reference test was conducted.

7c. Was quality control done?

To ensure reliability the test results for a subset of the sample population may be cross checked by a second person or an expert in echocardiographic imaging. Please indicate if this was done in the study. This might include all suspected cases (definite or borderline) on STAND being independently reviewed by a second reader with conflicts adjudicated by a third reader if necessary. If information is insufficient to make a judgment please tick the box 'unclear'. If no information is reported please tick the box 'Not reported' and if necessary, comment in the box provided.

7d. Is the reference standard likely to correctly classify the target condition?

- **Yes:** If measures to increase sensitivity are used e.g. multiple angles/images examined per patient and machine settings optimised.
- **No:** If for example only ill children are sampled for testing using the reference standard.
- **Unclear:** If there is insufficient information on the reference standard used or echo imaging technique used to make a judgment.

7e. Were the reference standard results interpreted without knowledge of the results of the index test?

- **Yes:** If results of the reference standard are interpreted without knowledge of index test results in cases when the reference test was used before the index test.

- **No:** If results of the reference test are interpreted with knowledge of the index test results in cases when the index test is performed before the reference test.
- **Unclear:** If there is insufficient information on when the index and reference tests were interpreted.
- **Not reported:** If no information was reported on this item.

7f. Could the conduct or interpretation of the reference standard have introduced bias?

- **Low:** If questions 7c – 7e were all answered 'yes'.
- **High:** If one or more of questions 7c – 7e were answered 'no'.
- **Unclear:** Any other combination of answers.

(8) TEST RESULTS

8a. Prevalence of disease based on STAND results [For those who received the reference test regardless of whether or not they also received the index test]
Please state the reported prevalence estimates for rheumatic heart disease based on the reference standard test results. Report these measures in terms of the mean, 95% confidence interval and range. Please report the results for all participants in the study who were screened for RHD using standard echocardiography. In some instances this indicates the larger cohort of participants who were all screened for RHD using STAND but of which some did not receive the index test as well. This is fine as long as all participants screened received the same reference standard test. If this information is not given or cannot be extracted from the results provided please tick the box 'Not reported' and add a comment in the space provided if necessary.

8b. Severity of disease [Only for those who received both the index and reference test]
Please indicate the range of disease severity for the participants included in the study who received both the index test (HAND) and the reference test (STAND) according to assessment by standard echocardiography. For example state the number or proportion of participants who were diagnosed as having definite RHD, borderline RHD or normal echocardiographic findings according to assessment by standard echocardiography. If no information is given please tick the box 'Not reported'.

8c. Missing data

Please indicate if there were missing, uninterpretable or unavailable results for both the reference standard and index tests by ticking the appropriate boxes. These may or may not be reported by the

For peer review only

authors. Please explain your answers in the comment boxes provided citing the number of missing / uninterpretable / unavailable test results.

To understand the following section better please refer to the paper by van Stralen K et al (2009). 'Diagnostic Methods I: sensitivity, specificity, and other measures of accuracy'. Kidney International, 75; 1257-1263.

8d. Accuracy results

Please ONLY report accuracy results for handheld echocardiography (HAND). Do not report results for auscultation or any other tests.

- Please extract the required data into the 2 by 2 table by listing the results in the spaces provided using the following format: True Positives (TP), True Negatives (TN), False Positives (FP), False Negatives (FN), Sensitivity (Sens), Specificity (Spec), Positive Predictive Value (PPV) and Negative Predictive Value (NPV). Please also extract 95% confidence intervals for measures of sensitivity, specificity, PPV and NPV if and when reported by study authors.

- In the first column (**Test**) please specify whether the results refer to any RHD (definite or borderline) or to definite RHD only. Please also further specify, where applicable, whether the results are pooled/combined (i.e. for all experts/non-experts/nurses etc.) or if the results are for different individuals (i.e. Nurse A or Nurse B). Please report all permutations thereof, for instance report the results for nurse A, then for Nurse B and lastly both of their results combined.

- Please specify the threshold or cut-off value in terms of mitral regurgitation (MR) &/or aortic regurgitation (AR) in the second column (**Threshold**). If multiple different thresholds were used, please report the results for each threshold separately (i.e. each one on a different row).

General comments

At the end of the data extraction form a comment box has been provided for general comments about the paper you have evaluated. Please use this box when and if necessary.

For peer review only

Statistical Formulae and Calculations

Supposing the means are given by M1, M2 and M3 and the SD's are S1, S2 and S3.

Let N1, N2 AND N3 represent the respective numbers of observations for each group.

Formula for and calculation of the pooled mean:

Pooled mean = (N1*M1+N2*M2+N3*M3) / (N1+N2+N3)

= (1217*9.6+397*13.9+956*11.1+1317*10.8+1420*10.8) / (1217+397+956+1317+1420)

= 10.8 years

Formula for and calculation of the pooled SD:

Pooled SD = {(N1-1)*S1+(N2-1)*S2+(N3-1)S3} / (N1+N2+N3-3)

= {(1217-1)*0.5+(397-1)*2.6+(956-1)*2.5+(1317-1)*2.6+(1420-1)*2.6} /

(1217+397+956+1317+1420)

= 2.1

For peer review only

PRISMA-DTA 2018 Checklist

Section and Topic	Item No.	Description	Page No.
**Title**			
Title	1	Identify the report as a systematic review (meta-analysis) of DTA studies.	1
**Abstract**			
Abstract	2	Provide a structured summary including, as applicable: background; objectives; data sources; study eligibility criteria, participants, and interventions; study appraisal and synthesis methods; results; limitations; conclusions and implications of key findings; systematic review registration number.	3 - 4
**Introduction**			
Rationale	3	Describe the rationale for the review in the context of what is already known.	5 - 6
Clinical role of index test	D1	State the scientific and clinical background, including the intended use and clinical role of the index test, and if applicable, the rationale for minimally acceptable test accuracy (or minimum difference in accuracy for a comparative design).	5
Objectives	4	Provide an explicit statement of questions being addressed in terms of participants, index test, and target conditions.	See protocol
**Methods**			
Protocol and registration	5	Indicate where the review protocol can be accessed (eg, web address) and provide trial registration number if available.	6
Eligibility criteria	6	Specify study characteristics (participants, setting, index test, reference standards, target conditions, and study design) and report characteristics (e.g., years considered, language, publication status) used as criteria for eligibility and providing rationale.	6 - 7
Information sources	7	Describe all information sources (eg, databases with dates of coverage, contact with study authors to identify additional studies) in the search and the date last searched.	6, 9 & 10
Search	8	Present full search strategies for all electronic databases and other sources searched, including any limits used so that they can be repeated.	See protocol
Study selection	9	State the process for selecting studies (e.g., screening, eligibility, whether included in systematic review, and, if applicable, included in the meta-analysis).	6 - 8
Data collection process	10	Describe methods of data extraction from reports (e.g., piloted forms, independently, in duplicate) and any processes for obtaining and confirming data from investigators.	7
Definitions for data extraction	11	Provide definitions used in data extraction and classifications of target conditions, index tests, reference standards, and other characteristics (eg, study design, clinical setting).	See supplementary data
Risk of bias and applicability	12	Describe methods used for assessing risk of bias in individual studies and concerns regarding the applicability to the review question.	7- 8
Diagnostic accuracy measures	13	State the principal diagnostic accuracy measures reported (eg, sensitivity, specificity) and state the unit of assessment (eg, per patient vs per lesion).	7

For peer review only

Section and Topic	Item No.	Description	Page No.
Synthesis of results	14	Describe the methods of handling the data, combining the results of the studies and describing the variability between studies. This could include, but is not limited to (1) handling of multiple definitions of the target condition, (2) handling of multiple thresholds of test positivity, (3) handling multiple index test readers, (4) handling of indeterminate test results, (5) grouping and comparing tests, and (6) handling of different reference standards.	8 - 9
Meta-analysis	D2	Report statistical methods used for meta-analyses if performed.	8 - 9
Additional analyses	16	Describe the methods of the additional analyses (eg, sensitivity or subgroup analyses, meta-regression), if done, indicating which were prespecified.	8 - 9
**Results**			
Study selection	17	Provide the numbers of studies screened, assessed for eligibility, included in the review, and included in the meta-analysis if applicable, with reasons for exclusions at each stage, ideally with a flow diagram.	9 - 10
Study characteristics	18	For each included study, provide citations and present key characteristics including (1) participant characteristics (presentation, prior testing), (2) clinical setting, (3) study design, (4) target condition definition, (5) index test, (6) reference standard, (7) sample size, and (8) funding sources.	10 - 11
Risk of bias and applicability	19	Present evaluation of risk of bias and concerns regarding applicability for each study.	12
Results of individual studies	20	For each analysis in each study (eg, unique combination of index test, reference standard, and positivity threshold), report 2 × 2 data (TP, FP, FN, TN) with estimates of diagnostic accuracy and confidence intervals, ideally with a forest plot or a receiver operating characteristic curve.	See https://zivahub.uct.ac.za/projects/STAND_vs_HAND_for_RHD_detection_A_DTA_SR/76395
Synthesis of results	21	Describe test accuracy, including variability; if meta-analysis was done, include results and confidence intervals.	12, 13 & 17
Additional analysis	23	Give results of additional analyses, if done (eg, sensitivity or subgroup analyses, meta-regression, analysis of index test, failure rates, proportion of inconclusive results, and adverse events).	14 - 15
**Discussion**			
Summary	24	Summarize the main findings including the strength of evidence.	15 - 18
Limitations	25	Discuss limitations from included studies (eg, risk of bias and concerns regarding applicability) and from the review process (eg, incomplete retrieval of identified research).	18 - 19
Conclusions	26	Provide a general interpretation of the results in the context of other evidence. Discuss implications for future research and clinical practice (eg, the intended use and clinical role of the index test).	19 - 21
**Funding**			
Funding	27	For the systematic review, describe the sources of funding and other support and the role of the funders.	See funding statement

Abbreviations: FN, false negative; FP, false positive; TN, true negative; TP, true positive

From: McInnes MDF, Moher D, Thombs BD, McGrath TA, Bossuyt PM; and the PRISMA-DTA Group (2018). Preferred Reporting Items for a Systematic Review and Meta-analysis of Diagnostic Test Accuracy Studies: The PRISMA-DTA Statement. JAMA 319(4): 388 - 396. doi: 10.1001/jama.2017.19163.

For peer review only - http://bmjopen.bmj.com/site/about/guidelines.xhtml

BMJ Open

Standard echocardiography versus handheld echocardiography for the detection of subclinical rheumatic heart disease: A systematic review and meta-analysis of diagnostic accuracy

Journal:	*BMJ Open*
Manuscript ID	bmjopen-2020-038449.R1
Article Type:	Original research
Date Submitted by the Author:	29-Jul-2020
Complete List of Authors:	Telford, Lisa; University of Cape Town Faculty of Health Sciences, Department of Medicine, Groote Schuur Hospital; University of Cape Town Faculty of Health Sciences, Department of Paediatrics and Child Health, Red Cross War Memorial Children's Hospital<br>Abdullahi, Leila; University of Cape Town Faculty of Health Sciences, Department of Paediatrics and Child Health, Red Cross War Memorial Children's Hospital; African Institute for Development Policy (AFIDEP)<br>Ochodo, Eleanor; University of Stellenbosch Centre for Evidence-Based Health Care, Department of Global Health, Faculty of Medicine and Health Sciences<br>ZUHLKE, LIESL; University of Cape Town Faculty of Health Sciences, Division of Cardiology, Department of Paediatrics and Child Health, Red Cross War Memorial Children's Hospital; University of Cape Town Faculty of Health Sciences, Division of Cardiology, Department of Medicine, Groote Schuur Hospital<br>Engel, Mark; University of Cape Town Faculty of Health Sciences, Department of Medicine, Groote Schuur Hospital
**Primary Subject Heading**:	Cardiovascular medicine
Secondary Subject Heading:	Evidence based practice, Diagnostics, Public health
Keywords:	Echocardiography < CARDIOLOGY, Paediatric cardiology < CARDIOLOGY, Valvular heart disease < CARDIOLOGY, PUBLIC HEALTH, Cardiovascular imaging < RADIOLOGY & IMAGING

SCHOLARONE™
Manuscripts

I, the Submitting Author has the right to grant and does grant on behalf of all authors of the Work (as defined in the below author licence), an exclusive licence and/or a non-exclusive licence for contributions from authors who are: i) UK Crown employees; ii) where BMJ has agreed a CC-BY licence shall apply, and/or iii) in accordance with the terms applicable for US Federal Government officers or employees acting as part of their official duties; on a worldwide, perpetual, irrevocable, royalty-free basis to BMJ Publishing Group Ltd ("BMJ") its licensees and where the relevant Journal is co-owned by BMJ to the co-owners of the Journal, to publish the Work in this journal and any other BMJ products and to exploit all rights, as set out in our licence.

The Submitting Author accepts and understands that any supply made under these terms is made by BMJ to the Submitting Author unless you are acting as an employee on behalf of your employer or a postgraduate student of an affiliated institution which is paying any applicable article publishing charge ("APC") for Open Access articles. Where the Submitting Author wishes to make the Work available on an Open Access basis (and intends to pay the relevant APC), the terms of reuse of such Open Access shall be governed by a Creative Commons licence – details of these licences and which Creative Commons licence will apply to this Work are set out in our licence referred to above.

Other than as permitted in any relevant BMJ Author's Self Archiving Policies, I confirm this Work has not been accepted for publication elsewhere, is not being considered for publication elsewhere and does not duplicate material already published. I confirm all authors consent to publication of this Work and authorise the granting of this licence.

For peer review only

Standard echocardiography versus handheld echocardiography for the detection of subclinical rheumatic heart disease: A systematic review and meta-analysis of diagnostic accuracy

For peer review only

Lisa H Telford[1,2], Leila H Abdullahi[2,3], Eleanor A Ochodo[4], Liesl J Zühlke[5,6], Mark E Engel[1]

Lisa H Telford

[1]Department of Medicine, Groote Schuur Hospital and University of Cape Town, Cape Town, South Africa.

[2]Department of Paediatrics and Child Health, Red Cross War Memorial Children's Hospital and University of Cape Town, Cape Town, South Africa.

Email: lisa.telford@uct.ac.za

ORCID ID: https://orcid.org/0000-0002-8554-057X

Leila H Abdullahi

[2]Department of Paediatrics and Child Health, Red Cross War Memorial Children's Hospital and University of Cape Town, Cape Town, South Africa.

[3]African Institute for Development Policy (AFIDEP), Nairobi, Kenya.

Email: leylaz@live.co.za

ORCID ID: None

Eleanor A Ochodo

[4]Centre for Evidence-based Health Care, Department of Global Health, Faculty of Medicine and Health Sciences, Stellenbosch University, Cape Town, South Africa.

Email: eochodo@sun.ac.za

ORCID ID: https://orcid.org/0000-0002-7951-3030

Liesl J Zühlke

[5]Division of Cardiology, Department of Medicine, Groote Schuur Hospital and University of Cape Town, Cape Town, South Africa.

[6]Division of Cardiology, Department of Paediatrics and Child Health, Red Cross War Memorial Children's Hospital and University of Cape Town, Cape Town, South Africa.

Email: liesl.zuhlke@uct.ac.za

ORCID ID: https://orcid.org/0000-0003-3961-2760

Mark E Engel

[1]Department of Medicine, Groote Schuur Hospital and University of Cape Town, Cape Town, South Africa.

Email: mark.engel@uct.ac.za

ORCID ID: https://orcid.org/0000-0002-1334-882

Correspondence to: Ms Lisa H Telford

Room F3, F-floor, Red Cross War Memorial Children's Hospital, Main Hospital Building, Klipfontein Road Rondebosch 7700, South Africa

+27 21 650 2438

lisa.telford@uct.ac.za

Keywords: rheumatic heart disease, echocardiography, diagnostic accuracy, systematic review, meta-analysis

Word count: 3361

For peer review only

ABSTRACT

Objective: To summarise the accuracy of handheld echocardiography (HAND) which, if shown to be sufficiently similar to that of standard echocardiography (STAND), could usher in a new age of rheumatic heart disease (RHD) screening in endemic areas.

Design: Systematic review and meta-analysis.

Data sources: PubMed, Scopus, EBSCOHost and ISI Web of Science were searched for studies published from 2012 onwards.

Eligibility criteria: Studies assessing the accuracy of HAND compared to STAND when performed by an experienced cardiologist in conjunction with the 2012 World Heart Federation criteria among populations of children and adolescents living in endemic areas were included.

Data extraction and synthesis: Two reviewers independently extracted data and assessed the methodological quality of included studies against review specific QUADAS-2 criteria. A meta-analysis using the Hierarchical Summary Receiver Operating Characteristic model was conducted to produce summary results of sensitivity and specificity. Forest plots and scatter plots in Receiver Operating Characteristic space in combination with subgroup analyses were used to investigate heterogeneity. Publication bias was not investigated.

Results: Six studies (N=4208) were included in the analysis. For any RHD detection, the pooled results from six studies were; sensitivity: 81.6% (95% CI: 76.5%-86.6%) and specificity: 89.8% (84.5%-95.0%). Meta-analytical results from five of the six included studies

For peer review only

were; sensitivity: 91.1% (80.5%-100%) and specificity: 92.0% (85.6%-98.4%) for the detection of definite RHD only and sensitivity: 62.0% (31.8%-92.2%) and specificity: 82.3% (65.2%-99.5%) for the detection of borderline RHD only.

Conclusions: HAND displayed good accuracy for detecting definite RHD only and modest accuracy for detecting any RHD but demonstrated poor accuracy for the detection of borderline RHD alone. Findings from this review provide some evidence for the potential of HAND to increase access to echocardiographic screening for RHD in resource-limited and remote settings, however, further research into feasibility and cost-effectiveness of wide-scale screening is still needed.

PROSPERO registration number: PROSPERO CRD42016051261.

Strengths and limitations of this study:

- Language restrictions were not imposed during the literature search to minimise the chance of missing studies.
- Data extraction was performed by two independent reviewers thereby reducing the risk of bias.
- Insufficient reporting limited our ability to adequately assess risk of bias and investigate potential sources of heterogeneity.
- The small number of included studies prevented us from performing meta-regression.

For peer review only

INTRODUCTION

Rheumatic heart disease (RHD) is an acquired permanent heart valve condition which results from an atypical immune reaction to group A streptococcal (GAS) infection typically occurring in childhood.[1,2] Disease progression leading to chronic RHD can result in irreversible heart valve damage, cardiac failure, and premature death.[3,4] RHD is, however, a preventable and treatable chronic condition which most often effects disadvantaged populations.[3,5]

Significantly, RHD can remain asymptomatic for many years, particularly during the initial stages, thereby hindering the timely implementation of penicillin prophylaxis.[6] Echocardiographic screening to identify those with subclinical disease has been advocated as a means to support secondary prevention and potentially slow disease progression to overt clinical RHD.[7,8] Yet the feasibility of wide scale echocardiographic screening remains hindered by high costs and the scarcity of trained personnel.[9] Alternative RHD screening tests which are both accurate and affordable are therefore needed in many endemic areas.

Handheld echocardiography (HAND) is a non-invasive, highly portable and comparatively less expensive device which has been presented in recent publications to be a promising alternative to standard echocardiography (STAND), despite some limitations such as a lack of spectral Doppler capabilities.[10,11] For HAND to be considered a suitable replacement for STAND, the device's accuracy needs to be similar to that of STAND.

We conducted a systematic review and meta-analysis of studies assessing the diagnostic accuracy of HAND for the detection of RHD in children and adolescents. The findings of this

For peer review only - http://bmjopen.bmj.com/site/about/guidelines.xhtml

review may offer direction to guideline developers as well as assist with the identification of gaps in diagnostic testing for RHD in endemic areas.

METHODS

This systematic review was prepared according to the Preferred Reporting Items for a Systematic Review and Meta-Analysis of Diagnostic Test Accuracy Studies (PRISMA-DTA) guidelines.[12] The protocol for this review is registered with the International Prospective Register of Systematic Reviews (PROSPERO) under the registration number CRD42016051261 and has been published in BMJ Open.[13]

Patient and public involvement

No patient involved.

Data sources and study eligibility

Studies were considered eligible for inclusion if the following criteria were met: 1) the accuracy of HAND compared to STAND when performed by an experienced cardiologist and in conjunction with the 2012 World Heart Federation (WHF) criteria was evaluated, and 2) the sample consisted of populations of children and adolescents living in endemic areas. Only primary observational studies of either a cross-sectional, cohort or diagnostic case-control design were considered. Descriptive studies such case studies and case series were excluded as were studies reporting on the same data. Studies using non-handheld devices as the index test or criteria other than the 2012 WHF criteria in combination with STAND as the reference test were also excluded.

For peer review only

We conducted systematic electronic literature searches of four sources (PubMed, Scopus, EBSCOHost and ISI Web of Science) using predefined tailor-made strategies (see supplementary file 1). No restrictions in terms of language were applied, however, searches were limited to articles published from 2012 onwards. Both published and unpublished literature was considered eligible for inclusion. A manual search of the reference lists of all included studies as well as relevant review articles was also conducted.

The titles and/or abstracts of all identified articles were screened independently by two reviewers. During this process, and on the basis of predefined eligibility criteria, all clearly ineligible studies were excluded. Discrepancies regarding eligibility were resolved through discussion and consensus. Some authors were contacted for additional information on published data.

Data extraction and management

Using a predefined data extraction form, two reviewers independently extracted information on metrics of diagnostic accuracy: numbers of true positives (TP), false positives (FP), true negatives (TN) and false negatives (FN) as well as other covariates relating to study characteristics, population, reference and index test details, test outcome and number of missing or unavailable test results from all included studies.

Accuracy measures: sensitivity and specificity were calculated using the numbers of TP, FP, TN and FN in accordance with standard convention. Data extraction conflicts were resolved through discussion and with the assistance of a third reviewer where necessary. Information garnered through the data extraction process was used to determine each study’s quality as well as for synthesising evidence.

For peer review only - http://bmjopen.bmj.com/site/about/guidelines.xhtml

Assessment of methodological quality

A review-specific Quality Assessment of Diagnostic Accuracy Studies (QUADAS-2) tool was used to assess the risk of bias and concerns regarding applicability of all included studies.[14] The tool, encompassing four domains, was tailored to meet the specific requirements of this review. Two reviewers independently assessed the risk of bias in all included studies according to review-specific QUADAS-2 criteria. Discrepancies were resolved through discussion until consensus was reached and the assistance of a third reviewer was enlisted when necessary.

Statistical analysis and data synthesis

A meta-analysis using the Hierarchical Summary Receiver Operating Characteristic (HSROC) model was conducted to produce summary results of sensitivity and specificity. The HSROC model was used for meta-analysis as it accounts for variations in test thresholds.

Statistical measures of variability or heterogeneity to determine whether or not to conduct a meta-analysis were not used. While the Cochrane Q test and I^2 are routinely used to examine and quantify the amount of variability in meta-analyses of intervention studies where there is a single measure of effect or univariate outcome such as a risk or odds ratio,[15][16] meta-analyses of diagnostic test accuracy generally do not employ such measures.[17] Instead, forest plots and scatter plots in Receiver Operating Characteristic (ROC) space in combination with subgroup analyses were used to investigate heterogeneity.

Data were analysed according to three categorisations of RHD; any RHD (definite or borderline), definite RHD only and borderline RHD only. The any RHD category was selected

For peer review only

as the main meta-analysis as it had the most complete data. We were unable to extract metrics of diagnostic accuracy for the definite and borderline RHD only categories from Beaton, 2016 and therefore excluded this study from these meta-analyses. We chose to use Nurse A's results for Mirabel, 2015 since Nurse A and Nurse B both interpreted the same HAND images which prevented the pooling of data.

Data from Zühlke, 2016 were included in the analysis and synthesis of data even though the age range of participants fell outside the predefined range for eligibility. It was determined that this study should be included, regardless, since the data overall were quite few and the variation in age was not significant enough to warrant exclusion. However, data from Zühlke, 2016 were excluded from all summary estimates of disease prevalence since this study used a nested case-control design which predetermines disease prevalence by design.

Heterogeneity was examined for the main meta-analysis only. We were only able to investigate the relationship between test accuracy and echocardiographer expertise through subgroup analysis. A sensitivity analysis was performed instead of subgroup analysis for the categorical covariates; HAND protocol and geographic location due to the skewed distribution of studies within each subgroup. We were unable to perform meta-regression for the covariates; age and sex due to insufficient and inadequately reported data.[17] We were also unable to conduct a sensitivity analysis on risk of bias since no studies were found to have a high risk of bias. All plots were generated using the Review Manager (RevMan) software package, version 5.3.[18] Meta-analysis was performed using SAS® software, version 9.4.[19]

We did not investigate publication bias as methods of assessing publication bias for studies of diagnostic accuracy are still being developed. While the Deeks test has been suggested

For peer review only

for use in diagnostic accuracy studies, the test has low power for detecting asymmetry in funnel plots, particularly when a large amount of heterogeneity is present.[17]

RESULTS

Results of the search

Results of the literature search are reported in accordance with the PRISMA Statement and the study selection process is illustrated in figure 1.[20] All electronic searches were performed by two independent reviewers on September 27th, 2017. Combined, the search yielded a total of ninety-two records, of which nine were duplicates. A total of sixty-seven were excluded based on title or abstract leaving sixteen for full-text review. Nine studies were excluded on the basis of a full-text review whilst an additional study was also excluded after consultation with study authors. Six studies which met the predefined eligibility criteria were included in this review.

Figure 1. PRISMA flow diagram illustrating study identification, selection, eligibility and inclusion. *From* Moher et al.[20]

The same search was re-run on March 3rd, 2020 to check for any additional eligible studies. Only one potentially eligible study [21,22] was found but has been excluded on the basis of being an abstract only publication with no full-text available for review.

Included studies

A summary of notable characteristics of all included studies [23–28] is shown in table 1. One study did not avoid a case-control design, however, cases and controls were sampled from the same population. Research has shown that case-control studies which use alternative

For peer review only - http://bmjopen.bmj.com/site/about/guidelines.xhtml

diagnosis controls, controls from non-endemic areas or confirmed disease-free (healthy) controls tend to overestimate specificity.[29]

Significantly, all but two studies were conducted in Africa. Screening was performed in RHD endemic areas among children and adolescents with most studies being school based. Combined, all six studies included a total of 4208 participants of which 54% were female. The pooled mean age of participant's was 10.8 years (SD: ±1.9).

All included studies used the same make of handheld device; the Vscan machine (General Electric, Medical Systems, Milwaukee, Wisconsin, USA) paired with a 1.7 to 3.4 MHz transducer. These machines provide both two-dimensional (2D) and colour imaging on an integrated 8.9cm display.[24,26] Frame rates range from 25 to 30 Hz for greyscale imaging and 12 to 16 Hz for colour Doppler.[23,27] Vscan machines are, however, limited by a lack of spectral Doppler capabilities.[24]

For peer review only

Table 1. Summary of characteristics of included studies: ordered alphabetically by study author.

**Study**	**Study Design**	**City, Country** (Classification¤)	**Recruitment Site/Setting**	**Participant Selection Method**	**Sample Size** (N)	**% Female**	**Mean Age** (years)	**SD**
**Beaton, 2014**[23]	Spiked cohort	Kampala, Uganda (Low-income)	A School and the Mulago Hospital Complex	Unclear	125	55.2	10.8¥	-
**Beaton, 2015**[24]	Cross-sectional	Gulu, Uganda (Low-income)	5 public schools	A random 10% subset of the entire sample plus any child with mitral or aortic regurgitation were preselected to receive HAND	1420	53	10.8	±2.6
**Beaton, 2016**[25]	Cross-sectional	Belo Horizonte, Brazil (Upper-middle income)	2 primary and 3 secondary public schools	A subset of the sample containing all STAND abnormals plus a random 25% of all STAND normals were preselected for HAND	397	49.1	13.9	±2.6
**Mirabel, 2015**[26]	Cross-sectional	Nouméa, New Caledonia (High-income)	Primary schools	Consecutive	1217	50.5	9.6	±0.5
**Ploutz, 2016**[27]	Cross-sectional	Gulu, Uganda (Low-income)	2 primary schools	Consecutive	956	60.7	11.1	±2.5
**Zühlke, 2016**[28]	Nested case-control	Cape Town, South Africa (Upper-middle income)	Schools	Unclear	93	68.8	17¥†	-
**Total**					4208	54	10.8	±1.9

¤According to the World Bank's economic classification system; ¥excluded from pooled mean & SD calculations (incomplete or incomparable data); †median age (mean age not available)

Excluded studies

Ten studies [9,10,30–37] were excluded during full-text screening. Reasons for exclusion included abstract only publications [32,33,35], the use of ineligible reference [30,31] or index [34] tests, the use of duplicate data [10,37], not specifying the test threshold a-priori [9], and not being a study of diagnostic accuracy [36].

Methodological quality of included studies

A summary of the assessment of methodological quality of all included studies is illustrated in figure 2. Overall, only two [25,26] of the six [23–28] included studies were assessed as having a low risk of bias while the risk in the remaining four [23,24,27,28] was unclear. Two studies [23,28] had participant selection bias concerns. Of these both [23,28] failed to adequately describe participant enrolment methods whilst one [28] also did not avoid a case-control design. The risk of bias in terms of flow and timing was unclear in two studies [24,27]. Of these, one [27] did not include all participants in the analysis due to technical difficulties while the time interval between the index and reference test was unclear in the other [24]. Overall, time intervals between index and reference tests were poorly described. Likewise, reporting of quality control of the index test was uniformly poor across all included studies. Concerns regarding applicability were low in all six [23–28] studies.

Figure 2. Risk of bias and applicability concerns graph: review authors' judgments about each domain presented as percentages across included studies.

Findings

For peer review only

For any RHD

A total of six evaluations of HAND for any RHD were performed with data from six studies and a total of 4208 participants. Pooled prevalence of any RHD from five included studies was 12% (95% confidence interval (CI): 6%-19%). The forest plot revealed little variation in estimates of sensitivity and specificity and the HSROC plot (see supplementary file 2 for all plots) revealed moderate accuracy of the test. Meta-analytical sensitivity and specificity (95% CI) of data at mixed thresholds were 82% (77%-87%) and 90% (85%-95%) respectively.

For definite RHD

A total of five evaluations of HAND for definite RHD were performed with data from five studies and a total of 3588 participants. Pooled prevalence of definite RHD from four included studies was 6% (95% CI: 2%-12%). The forest plot revealed some variation in estimates of specificity while estimates of sensitivity were largely homogenous with the exception of a single outlier. The HSROC plot indicated good accuracy of the test. Meta-analytical sensitivity and specificity (95% CI) of data at mixed thresholds were 91% (81%-100%) and 92% (86%-98%) respectively.

For borderline RHD

A total of five evaluations of HAND for borderline RHD were performed with data from five studies and a total of 3685 participants. Pooled prevalence of borderline RHD from four included studies was 20% (95% CI: 6%-39%). The forest plot revealed some variation in estimates of specificity while estimates of sensitivity were largely homogenous with the exception of a single outlier. The HSROC plot indicated poor accuracy of the test. Meta-

For peer review only

analytical sensitivity and specificity (95% CI) of data at mixed thresholds were 62% (32%-92%) and 82% (65%-100%) respectively.

Investigations of heterogeneity

Heterogeneity or variation between studies was investigated both visually as well as through subgroup analysis for the main meta-analysis only. We were only able to perform this analysis for the any RHD category as the data were too few to enable model convergence for the definite and borderline RHD only categories.

Co-variates in the models

We were only able to use one of the five pre-specified covariates to investigate heterogeneity due to insufficient and inadequately reported data. HAND echocardiographer expertise (expert vs non-expert) was investigated as a possible source of heterogeneity through subgroup analysis. Half of all included studies evaluated the accuracy of HAND when performed and interpreted by trained non-experts while the other half assessed its accuracy in the hands of experts.

Subgroup and sensitivity analyses

A subgroup analysis was performed to investigate variations in echocardiographer expertise as a potential source of heterogeneity. Since no studies were found to have a high risk of bias we did not explore the effect of excluding such studies on the accuracy of summary estimates**.** Sensitivity analyses were, however, conducted to investigate the effect of removing a single study on summary estimates of sensitivity and specificity for the covariates; geographic location and HAND protocol.

For peer review only

A subgroup analysis for the covariate; echocardiographer expertise, as shown in table 2 and illustrated in supplementary file 3, revealed that both sensitivity (82.54% vs 80.76%) and specificity (94.57% vs 85.71%) were higher for any RHD detection using HAND when tests were performed and interpreted by experts compared to non-experts.

Sensitivity analyses (see table 2) were performed to investigate the effect of excluding a) the single high-income country study and b) the study which employed a single view protocol on the accuracy of summary estimates. We found that both sensitivity (81.17% vs 80.4%) and specificity (90.07% vs 87.15%) increased compared to the overall analysis when only low- and middle-income country studies were considered whereas both sensitivity (80.98% vs 85.35%) and specificity (87.46% vs 88.8%) decreased in comparison with the overall analysis when only studies which employed multiple view protocols were considered.

Table 2. Sources of heterogeneity for handheld echocardiography for any RHD.

**Group**	**Covariate**	**Subgroup**	**n (N=6)**	**Median Pooled Sensitivity** (95% Crl)	**Median Pooled Specificity** (95% Crl)
**Overall**			6	**81.56% (76.52-86.61)**	**89.75% (84.48-95.01)**
**Subgroup Analysis**	HAND Interpreter Expertise	Expert	3	82.54% (74.71-90.37)	94.57% (87.52-100)
		Non-expert	3	80.76% (73.18-88.33)	85.71% (79.91-91.51)
**Sensitivity Analysis**	Geographic Location	Overall		80.40% (64.96-95.84)	87.15% (76.35-97.96)
		Low- and middle-income countries	5	81.17% (75.63-86.71)	90.07% (82.98-97.15)
	HAND Protocol	Overall		85.35% (80.60-90.09)	88.80% (84.55-93.06)
		Multiple views	5	80.98% (75.38-86.59)	87.46% (83.24-91.67)

Abbreviations: HAND, handheld echocardiography; Crl, credible interval; n, number of studies; N, total number of included studies

Table 3. Summary of findings.

What is the diagnostic accuracy of handheld echocardiography in detecting any RHD (definite or borderline)?	
**Patients/Population**	People residing in areas endemic for RHD (6 out of 6 studies)
**Prior testing with echo**	Yes (2 studies), No (4 studies)
**Settings**	5 out of 6 screening studies were field setting (communities and schools) based while 1 study was half hospital registry follow-up, half school based.<br>4 of the 6 studies were conducted in Africa with 3 of those from Uganda.
**Index test(s)**	General Electric (GE) Vscan handheld machine (6 out of 6 studies)
**Reference standard**	Standard echocardiography (2D, continuous-wave, and colour-Doppler echocardiography) performed by an experienced imager and in conjunction with the 2012 WHF criteria (6 out of 6 studies).<br>Test brands included; GE Vivid-I ultrasound machine (2 studies)<br>; GE Vivid-Q ultrasound machine (2 studies)<br>; Philips CX-50 ultrasound machine (1 study)

DISCUSSION

Summary of main findings

We evaluated the accuracy of HAND for three distinct disease categories and found that, overall, the test was both sensitive and specific for detecting definite RHD only and moderately accurate for detecting any RHD but demonstrated insufficient accuracy for detecting borderline RHD alone.

Findings from this review provide some evidence for the potential of HAND to increase access to echocardiographic screening for RHD in resource-limited and remote settings. A summary of the accuracy estimates produced by meta-analysis using the HSROC method is included in table 3.

review only

	; Either a GE Vivid-I or Q or Philips CX-50 ultrasound machine (1 study)	
**Importance**	HAND is being used as first line replacement for STAND in disease screening programmes for RHD, as it is comparably inexpensive, quick, user friendly, easy to interpret, and may have similar sensitivity to STAND.	
**Studies**	Cross-sectional (n=4), spiked cohort (n=1) and nested case-control (n=1) studies. More than half (n=4) of all included studies did not explicitly state the study design used and were thus assigned a study design based on other reported characteristics and participant enrolment methods used.	
**Quality concerns**	Poor reporting of study design, participant characteristics and pre-test probability were common concerns. For the majority of studies the risk of bias was unclear in terms of 'patient selection' and 'flow and timing'. Concerns regarding applicability were low in all included studies.	
**Test types**	**Number of participants*** (N)	**Summary estimates** (95% credible CI)
HAND for **any RHD**	4208 (6)	Sensitivity; 81.56% (76.52-86.61)<br>Specificity; 89.75% (84.48-95.01)
HAND for **definite RHD**	3588 (5)	Sensitivity; 91.06% (80.46-100)<br>Specificity; 91.96% (85.57-98.36)
HAND for **borderline RHD**	3685 (5)	Sensitivity; 62.01% (31.8-92.22)<br>Specificity; 82.33% (65.15-99.52)
Abbreviations: CI, confidence interval; HAND, handheld echocardiography; N, number of studies; RHD, rheumatic heart disease; STAND, standard echocardiography *Excluding participants with other diagnoses on STAND		

Strengths and limitations of this review

Strengths

We have evaluated and summarised the accuracy of HAND for the detection of RHD in endemic areas, making the review relevant to current global agendas. This review also serves to highlight the existing gaps in evidence for which further research could be beneficial. We did not impose any limits in terms of language during the literature search so

as to minimise the chance of missing studies. Data extraction was performed by two independent reviewers thereby reducing the risk of bias.

Limitations

There were a number of shortcomings of this review, which included;

Eligibility: We were unable to include studies which used STAND in conjunction with criteria other than the 2012 WHF criteria as the reference standard which limited the number of studies eligible for inclusion.

Quality of included studies: Insufficient reporting of participant characteristics and study methods including study design, participant selection and test timing restricted our ability to adequately assess risk of bias and investigate potential sources of heterogeneity.

Paucity of data: Insufficient and inadequately reported data as well as the presentation of aggregate data limited the scope of our investigations of heterogeneity while the small number of included studies prevented us from performing meta-regression. Overall, the findings from this review may lack power due to the small sample size.

Applicability of findings to the review question

Concerns regarding the applicability of included studies to the review question were considered low according to review-specific QUADAS-2 criteria. Since all but one were conducted in low- or middle-income countries, and all studies with one exception were conducted in field settings, the results of this review are applicable for use in endemic areas for which screening programmes are frequently targeted. However, our limited assessment

For peer review only

of risk of bias and investigations into sources of heterogeneity such as age and gender due to insufficient reporting may lessen the applicability of findings to the review question.

In the context of disease control programmes, being able to demonstrate variation in test accuracy associated with factors such as age and gender would be beneficial for policy makers. Fully understanding included studies' risk of bias would also assist in objectively assessing the strength of evidence. For these reasons, prospective authors of diagnostic test accuracy studies are urged to make use of the Standards for Reporting of Diagnostic Accuracy Studies (STARD) guidelines [38] when reporting methods of study design and conduct.

CONCLUSION

This review provides a summary of the accuracy of HAND for the detection of RHD. In populations of children and adolescents living in RHD endemic areas, HAND is both sensitive and specific for detecting definite RHD. The device is less accurate in detecting any RHD and demonstrates substandard accuracy for the detection of borderline RHD only. Nonetheless, this test may hold value as a replacement for first line screening due to its high sensitivity for definite RHD detection and adequate accuracy for any RHD detection.

Implications for practice

We have summarised the accuracy of HAND when used as a screening tool, however, the device's potential value in terms of diagnostics has yet to be established. We therefore posit that HAND could be recommended as an acceptable replacement test for first line screening

For peer review only

in endemic areas provided a standardised set of device-specific diagnostic criteria are developed.

Another key consideration is the applicability of these findings for recommendations to integrate screening into routine clinical practice. A recent publication has reviewed the cost-effectiveness of screening in high-risk populations [39] and determined that screening all indigenous Australian 5 to 12 year-olds in half of their communities in alternate years was found to be cost-effective, if RHD can be detected at least 2 years earlier. However, this result was sensitive to a number of assumptions, including local costs and context. Other cost-effectiveness models have also suggested modestly improved outcomes at lower cost.[40] Neither of these studies included the significant cost-reduction using HAND instead of STAND, hence, we highly recommend adding a cost-effectiveness analysis into proposed new screening studies.

Finally, our findings demonstrate comparable results by non-experts, this has also been demonstrated in several other reports [31,41], but again there are no detailed cost-effectiveness analyses using non-experts and HAND.

Implications for research

The findings of this review highlight the need for a new set of evidence-based guidelines tailored to the capabilities of HAND in order to maximise the device's diagnostic potential. Further studies assessing the diagnostic accuracy of HAND when using a standardised protocol are needed as is further research into the feasibility, cost-effectiveness and consequences of implementing wide scale screening programs. Furthermore, the development of standardised training programs for non-experts is recommended as

For peer review only

screening for RHD in endemic areas inevitably rests on the success of task shifting.[27] We conclude that while HAND has been shown to be sufficiently accurate for the detection of RHD there is still a need for further research before its' wide scale use can be endorsed.

Author affiliations

[1]Department of Medicine, Groote Schuur Hospital and University of Cape Town, Cape Town, South Africa.

[2]Department of Paediatrics and Child Health, Red Cross War Memorial Children's Hospital and University of Cape Town, Cape Town, South Africa.

[3]African Institute for Development Policy (AFIDEP), Nairobi, Kenya.

[4]Centre for Evidence-based Health Care, Department of Global Health, Faculty of Medicine and Health Sciences, Stellenbosch University, Cape Town, South Africa.

[5]Division of Cardiology, Department of Medicine, Groote Schuur Hospital and University of Cape Town, Cape Town, South Africa.

[6]Division of Cardiology, Department of Paediatrics and Child Health, Red Cross War Memorial Children's Hospital and University of Cape Town, Cape Town, South Africa.

For peer review only

Author contributions

LJZ and MEE jointly conceived of the study and provided content advice. LHT and LHA performed the systematic review of the literature, independently extracted the data and are responsible for the quality control of study selection. EAO provided methodological advice and was responsible for conducting the meta-analysis. LHT significantly contributed to the interpretation of the data and wrote the first draft of the manuscript. All authors contributed to editing subsequent versions of the manuscript.

Funding

LJZ, LHT and LHA all receive funding from the Medtronic Foundation through support to RHD Action. LHT receives support from the National Research Foundation of South Africa (NRFSA) through its internship programme. LJZ is supported by both the NRFSA and Medical Research Council (MRC).

Competing interests

None declared.

Patient consent for publication

Not required.

Provenance and peer review

Not commissioned; externally peer reviewed.

Data availability statement

Dataset, https://doi.org/10.25375/uct.11902077.v1, available from the ZivaHub repository. All other relevant data are included in the article or uploaded as supplementary information.

ORCID iDs

Lisa H Telford https://orcid.org/0000-0002-8554-057X

Eleanor A Ochodo https://orcid.org/0000-0002-7951-3030

For peer review only

Liesl J Zühlke https://orcid.org/0000-0003-3961-2760

Mark E Engel https://orcid.org/0000-0002-1334-882

References

1. Marijon E, Mirabel M, Celermajer DS, Jouven X. Rheumatic heart disease. *Lancet* [Internet] 2012;379(9819):953–64. Available from: https://doi.org/10.1016/S0140-6736(11)61171-9

2. Zühlke LJ, Beaton A, Engel ME, Hugo-Hamman CT, Karthikeyan G, Katzenellenbogen JM, et al. Group A Streptococcus, Acute Rheumatic Fever and Rheumatic Heart Disease: Epidemiology and Clinical Considerations. *Curr Treat Options Cardiovasc Med* [Internet] 2017;19(2):15. Available from: http://link.springer.com/10.1007/s11936-017-0513-y

3. World Heart Federation. Rheumatic Heart Disease: Neglected NCD of Poverty (Factsheet). Geneva: World Heart Federation; 2013.

4. Seckeler MD, Hoke T. The worldwide epidemiology of acute rheumatic fever and rheumatic heart disease. *Clin Epidemiol* [Internet] 2011;3(1):67–84. Available from: http://www.dovepress.com/the-worldwide-epidemiology-of-acute-rheumatic-fever-and-rheumatic-hear-peer-reviewed-article-CLEP

5. Dougherty S, Khorsandi M, Herbst P. Rheumatic heart disease screening: Current concepts and challenges. *Ann Pediatr Cardiol* [Internet] 2017;10(1):39. Available from: http://www.annalspc.com/text.asp?2017/10/1/39/197051

6. Moloi AH, Mall S, Engel ME, Stafford R, Zhu ZW, Zühlke LJ, et al. The Health Systems

For peer review only

Barriers and Facilitators for RHD Prevalence: An Epidemiological Meta-Analysis From Uganda and Tanzania. *Glob Heart* [Internet] 2017;12(1):5–15. Available from: https://globalheartjournal.com/article/10.1016/j.gheart.2016.12.002/

7. Zühlke LJ, Watkins DA, Perkins S, Wyber R, Mwangi J, Markbreiter J, et al. A Comprehensive Needs Assessment Tool for Planning RHD Control Programs in Limited Resource Settings. *Glob Heart* [Internet] 2017;12(1):25–31. Available from: http://dx.doi.org/10.1016/j.gheart.2016.10.028

8. Palafox B, Mocumbi AO, Kumar RK, Ali SKM, Kennedy E, Haileamlak A, et al. The WHF Roadmap for Reducing CV Morbidity and Mortality Through Prevention and Control of RHD. *Glob Heart* [Internet] 2017;12(1):47–62. Available from: http://dx.doi.org/10.1016/j.gheart.2016.12.001

9. Lu JC, Sable C, Ensing GJ, Webb C, Scheel J, Aliku T, et al. Simplified rheumatic heart disease screening criteria for handheld echocardiography. *J Am Soc Echocardiogr* [Internet] 2015;28(4):463–9. Available from: http://dx.doi.org/10.1016/j.echo.2015.01.001

10. Godown J, Lu JC, Beaton A, Sable C, Mirembe G, Sanya R, et al. Handheld Echocardiography Versus Auscultation for Detection of Rheumatic Heart Disease. *Pediatrics* [Internet] 2015;135(4):e939–44. Available from: http://search.ebscohost.com/login.aspx?direct=true&db=cmedm&AN=25780068&site=ehost-live

11. Saxena A. Rheumatic heart disease screening by “point-of-care” echocardiography: an acceptable alternative in resource limited settings? *Transl Pediatr* [Internet]

For peer review only

2015;4(3):210–3. Available from: http://www.ncbi.nlm.nih.gov/pubmed/26835377

12. McInnes MDF, Moher D, Thombs BD, McGrath TA, Bossuyt PM, and the PRISMA-DTA Group, et al. Preferred Reporting Items for a Systematic Review and Meta-analysis of Diagnostic Test Accuracy Studies: The PRISMA-DTA Statement. *JAMA* [Internet] 2018;319(4):388–96. Available from: http://jama.jamanetwork.com/article.aspx?doi=10.1001/jama.2017.19163

13. Telford LH, Abdullahi LH, Ochodo EA, Zühlke LJ, Engel ME. Standard echocardiography versus handheld echocardiography for the detection of subclinical rheumatic heart disease: protocol for a systematic review. *BMJ Open* [Internet] 2018;8(2):e020140. Available from: http://bmjopen.bmj.com/lookup/doi/10.1136/bmjopen-2017-020140

14. Whiting PF, Rutjes AWS, Westwood ME, Mallet S, Deeks JJ, Reitsma JB, et al. Research and Reporting Methods Accuracy Studies. *Ann Intern Med* 2011;155(4):529–36.

15. Higgins J, Thomas J, Chandler J, Cumpston M, Li T, Page M, et al., editors. Cochrane Handbook for Systematic Reviews of Interventions version 6.0 (updated July 2019) [Internet]. Cochrane; 2019. Available from: https://training.cochrane.org/handbook

16. Naaktgeboren CA, Ochodo EA, Van Enst WA, de Groot JAH, Hooft L, Leeflang MMG, et al. Assessing variability in results in systematic reviews of diagnostic studies. *BMC Med Res Methodol* [Internet] 2016;16(1):6. Available from: http://dx.doi.org/10.1186/s12874-016-0108-4

17. Macaskill P, Gatsonis C, Deeks JJ, Harbord RM, Takwoingi Y. Chapter 10: Analysing and Presenting Results. In: Deeks JJ, Bossuyt PM, Gatsonis C, editors. Cochrane Handbook for Systematic Reviews of Diagnostic Test Accuracy Version 1 [Internet] The Cochrane

For peer review only

Collaboration; 2010. Available from: https://methods.cochrane.org/sites/methods.cochrane.org.sdt/files/public/uploads/Chapter 10 - Version 1.0.pdf

18. Review Manager (RevMan). Copenhagen: The Nordic Cochrane Centre, The Cochrane Collaboration; 2014.

19. SAS/STAT Software. SAS Institute Inc.; 2011.

20. Moher D, Liberati A, Tetzlaff J, Altman DG, Altman DG, Antes G, et al. Preferred Reporting Items for Systematic Reviews and Meta-Analyses: The PRISMA Statement. *PLoS Med* [Internet] 2009;6(7):e1000097. Available from: http://www.ncbi.nlm.nih.gov/pubmed/19621072

21. Francis J, Fairhurst H, Kaethner A, Whalley G, Ryan C, Brown S, et al. Handheld PLAX View Echocardiography by Healthworkers for Detection of RHD. *Hear Lung Circ* [Internet] 2019;28:S51–2. Available from: https://linkinghub.elsevier.com/retrieve/pii/S1443950619305177

22. Francis J, Fairhurst H, Kaethner A, Whalley G, Ryan C, Dos Santos J, et al. P3133: Single parasternal long axis echocardiography by briefly trained health workers using handheld devices for detection of rheumatic heart disease: a prospective study of diagnostic accuracy. *Eur Heart J* [Internet] 2019;40(Supplement_1):3133. Available from: https://academic.oup.com/eurheartj/article/doi/10.1093/eurheartj/ehz745.0208/5597410

23. Beaton A, Aliku T, Okello E, Lubega S, McCarter R, Lwabi P, et al. The utility of

For peer review only

handheld echocardiography for early diagnosis of rheumatic heart disease. *J Am Soc Echocardiogr* [Internet] 2014;27(1):42–9. Available from: http://dx.doi.org/10.1016/j.echo.2013.09.013

24. Beaton A, Lu JC, Aliku T, Dean P, Gaur L, Weinberg J, et al. The utility of handheld echocardiography for early rheumatic heart disease diagnosis: a field study. *Eur Hear J - Cardiovasc Imaging* [Internet] 2015;16(5):475–82. Available from: https://academic.oup.com/ehjcimaging/article-lookup/doi/10.1093/ehjci/jeu296

25. Beaton A, Nascimento BR, Diamantino AC, Pereira GTR, Lopes ELV, Miri CO, et al. Efficacy of a Standardized Computer-Based Training Curriculum to Teach Echocardiographic Identification of Rheumatic Heart Disease to Nonexpert Users. *Am J Cardiol* [Internet] 2016;117(11):1783–9. Available from: http://dx.doi.org/10.1016/j.amjcard.2016.03.006

26. Mirabel M, Bacquelin R, Tafflet M, Robillard C, Huon B, Corsenac P, et al. Screening for Rheumatic Heart Disease: Evaluation of a Focused Cardiac Ultrasound Approach. *Circ Cardiovasc Imaging* [Internet] 2015;8(1):e002324–e002324. Available from: http://www.ncbi.nlm.nih.gov/pubmed/25567654

27. Ploutz M, Lu JC, Scheel J, Webb C, Ensing GJ, Aliku T, et al. Handheld echocardiographic screening for rheumatic heart disease by non-experts. *Heart* [Internet] 2016;102(1):35–9. Available from: http://10.0.4.112/heartjnl-2015-308236

28. Zühlke LJ, Engel ME, Nkepu S, Mayosi BM. Evaluation of a focussed protocol for hand-held echocardiography and computer-assisted auscultation in detecting latent rheumatic heart disease in scholars. *Cardiol Young* [Internet] 2016;26(06):1097–106.

For peer review only

Available from: https://www.scopus.com/inward/record.uri?eid=2-s2.0-84943741614&partnerID=40&md5=816a5cc70c6958d0909d9c3f9594c25a

29. Rutjes AWS, Reitsma JB, Vandenbroucke JP, Glas AS, Bossuyt PMM. Case–Control and Two-Gate Designs in Diagnostic Accuracy Studies. *Clin Chem* [Internet] 2005;51(8):1335–41. Available from: https://academic.oup.com/clinchem/article/51/8/1335/5629913

30. Mirabel M, Celermajer DS, Ferreira B, Tafflet M, Perier M-C, Karam N, et al. Screening for rheumatic heart disease: evaluation of a simplified echocardiography-based approach. *Eur Heart J Cardiovasc Imaging* [Internet] 2012;13(12):1024–9. Available from: https://academic.oup.com/ehjcimaging/article-lookup/doi/10.1093/ehjci/jes077

31. Colquhoun SM, Carapetis JR, Kado JH, Reeves BM, Remenyi B, May W, et al. Pilot study of nurse-led rheumatic heart disease echocardiography screening in Fiji--a novel approach in a resource-poor setting. *Cardiol Young* [Internet] 2013;23(4):546–52. Available from: http://www.ncbi.nlm.nih.gov/pubmed/23040535

32. Godown J, Lu JC, Beaton A, Sable C, Mirembe G, Sanya R, et al. Handheld Echocardiography Improves Detection of Rheumatic Heart Disease in Ugandan Schoolchildren Compared to Auscultation. *Circulation* [Internet] 2014;130(2). Available from: http://circ.ahajournals.org/content/130/Suppl_2/A11256.short

33. Lu JC, Sable C, Ensing GJ, Webb CC, Scheel J, Aliku T, et al. Abstract 11138: Simplified Rheumatic Heart Disease Screening Criteria for Handheld Echocardiography. *Circulation* [Internet] 2014;130(Suppl 2):A11138 LP-A11138. Available from:

For peer review only

http://circ.ahajournals.org/content/130/Suppl_2/A11138.abstract

34. Engelman D, Kado JH, Reményi B, Colquhoun SM, Carapetis JR, Donath S, et al. Focused cardiac ultrasound screening for rheumatic heart disease by briefly trained health workers: a study of diagnostic accuracy. *Lancet Glob Heal* [Internet] 2016;4(6):e386–94. Available from: http://linkinghub.elsevier.com/retrieve/pii/S2214109X16300651

35. Hardie J, Goodman N, Khan N, Barasa A, Ngunga M, Jutley R. Abstract: P1373 Feasibility of focused cardiac ultrasound by non-expert operators as a strategy for a rheumatic heart disease screening programme. *Eur Heart J* [Internet] 2016;37:241. Available from: http://doi.wiley.com/10.1111/ctr.12644

36. Lopes EL, Beaton AZ, Nascimento BR, Tompsett A, Dos Santos JP, Perlman L, et al. Telehealth solutions to enable global collaboration in rheumatic heart disease screening. *J Telemed Telecare* [Internet] 2016; Available from: http://search.ebscohost.com/login.aspx?direct=true&db=cmedm&AN=27815494&site=ehost-live

37. Diamantino A, Beaton A, Aliku T, Oliveira K, Oliveira C, Xavier L, et al. A focussed single-view hand-held echocardiography protocol for the detection of rheumatic heart disease. *Cardiol Young* [Internet] 2017;1–10. Available from: https://www.cambridge.org/core/product/identifier/S1047951117001676/type/journal_article

38. Bossuyt PM, Reitsma JB, Bruns DE, Gatsonis CA, Glasziou PP, Irwig L, et al. STARD 2015: an updated list of essential items for reporting diagnostic accuracy studies. *BMJ*

For peer review only

[Internet] 2015;277(3):h5527. Available from: http://www.bmj.com/lookup/doi/10.1136/bmj.h5527

39. Roberts K, Cannon J, Atkinson D, Brown A, Maguire G, Remenyi B, et al. Echocardiographic Screening for Rheumatic Heart Disease in Indigenous Australian Children: A Cost–Utility Analysis. *J Am Heart Assoc* [Internet] 2017;6(3):e004515. Available from: https://www.ahajournals.org/doi/10.1161/JAHA.116.004515

40. Zachariah JP, Samnaliev M. Echo-based screening of rheumatic heart disease in children: a cost-effectiveness Markov model. *J Med Econ* [Internet] 2015;18(6):410–9. Available from: http://www.tandfonline.com/doi/full/10.3111/13696998.2015.1006366

41. Musuku J, Engel ME, Musonda P, Lungu JC, Machila E, Schwaninger S, et al. Prevalence of rheumatic heart disease in Zambian school children. *BMC Cardiovasc Disord* [Internet] 2018;18(1):135. Available from: http://www.ncbi.nlm.nih.gov/pubmed/8406708

For peer review only

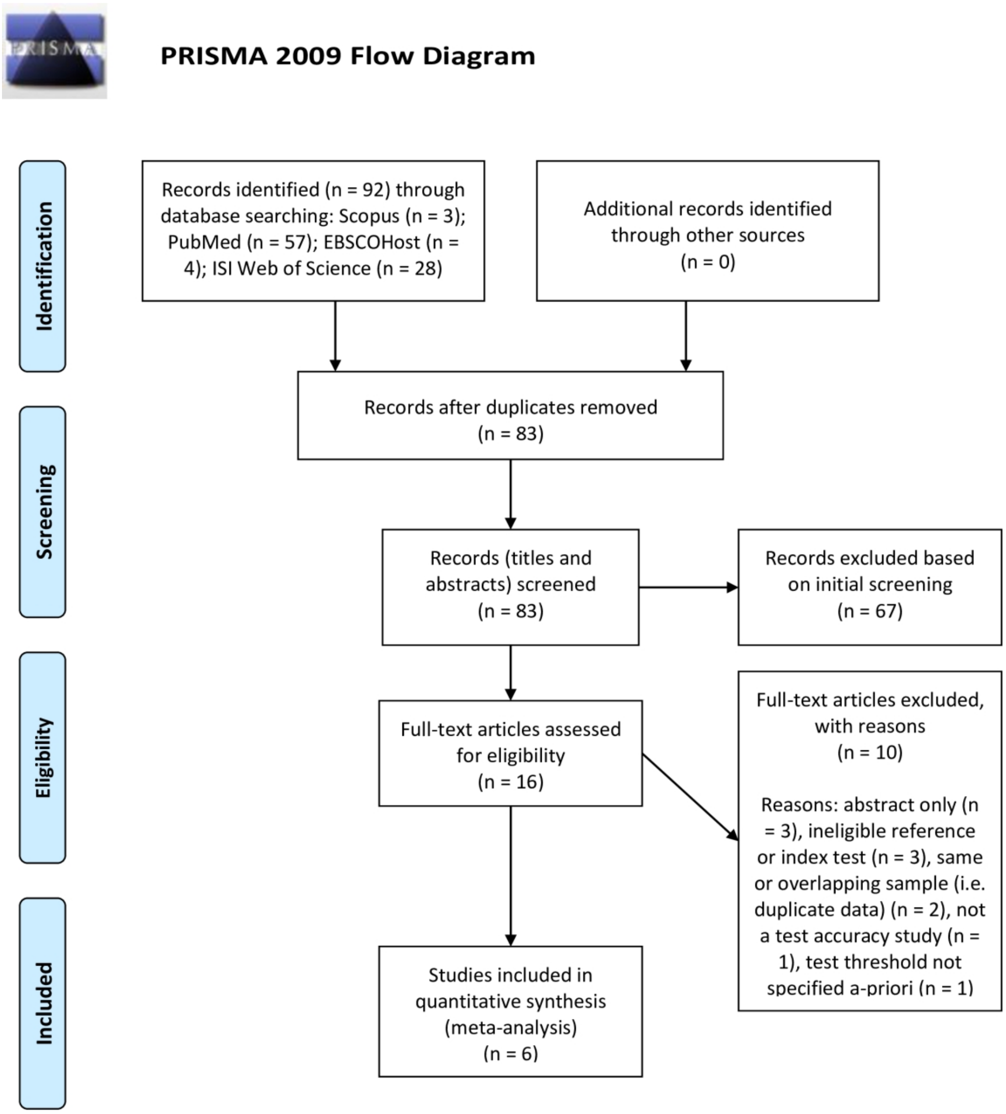

Figure 1. PRISMA flow diagram illustrating study identification, selection, eligibility and inclusion. From Moher et al.[20]

181x200mm (300 x 300 DPI)

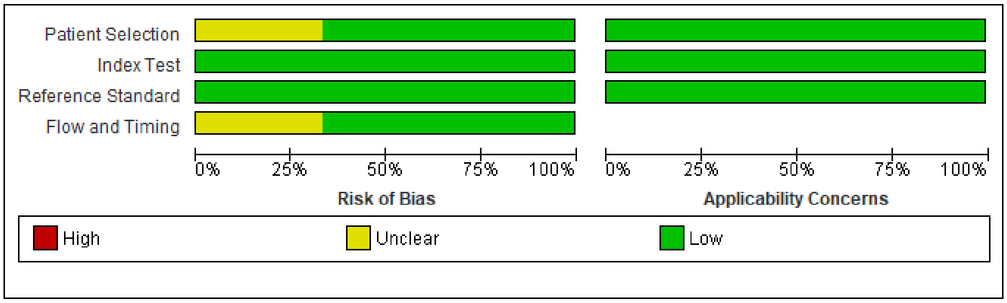

Figure 2. Risk of bias and applicability concerns graph: review authors' judgments about each domain presented as percentages across included studies.

Supplementary file 1. Search strategy.

Database	Search Terms	Limits
**PubMed**	((((((((((((((Hand-held) OR handheld) OR hand held) OR hand-carried) OR hand carried) OR HAND) OR HCU) OR HHCU) OR pocket size) OR pocket sized) OR portable) OR miniaturization) OR miniaturized) OR focused) OR focus)) AND (((("Echocardiography"[Mesh]) OR echocardiography) OR echocardiographic) OR cardiac ultrasound)) AND ((("Rheumatic Heart Disease"[Mesh]) OR rheumatic heart disease) OR RHD)<br>[MeSH terms were exploded during the search]	Limited to 2012-2020
**Scopus**	**1.** Hand-held OR handheld OR hand held OR hand-carried OR hand carried OR HAND OR HCU OR HHCU OR pocket size* OR portable OR miniatur* OR focus*<br>**2.** Echocardiograph* OR cardiac ultrasound<br>**3.** Rheumatic Heart Disease OR RHD<br>**#1 AND #2 AND #3**	Limited to 2012-2020
**ISI Web of Science**	**1.** Hand-held OR handheld OR hand held OR hand-carried OR hand carried OR HAND OR HCU OR HHCU OR pocket size OR pocket sized OR portable OR Miniaturization OR Miniaturized OR focused OR focus<br>**2.** Echocardiography OR Echocardiographic OR cardiac ultrasound<br>**3.** Rheumatic Heart Disease OR RHD<br>**Combine #1 AND #2 AND #3**	Limited to 2012–2020 filtering out MEDLINE
**EBSCO Host**	**S1.** Hand-held OR handheld OR hand held OR hand-carried OR hand carried OR HAND OR HCU OR HHCU OR pocket size OR pocket sized OR portable OR Miniaturization OR Miniaturized OR focused OR focus<br>**S2.** Echocardiography OR Echocardiographic OR cardiac ultrasound<br>**S3.** Rheumatic Heart Disease OR RHD<br>**S1 AND S2 AND S3**	Limited to 2012-2020

Supplementary file 2. Forest plots and Hierarchical Summary Receiver Operating Characteristic (HSROC) plots for three categorisations of RHD; any RHD (definite or borderline), definite RHD only and borderline RHD only.

*All plots were generated using the Review Manager (RevMan) software package, version 5.3

Any RHD:

Study	TP	FP	FN	TN	Expertise	Sensitivity (95% CI)	Specificity (95% CI)
Beaton (2014)	37	6	4	78	Expert	0.90 [0.77, 0.97]	0.93 [0.85, 0.97]
Beaton (2015)	141	158	39	1076	Expert	0.78 [0.72, 0.84]	0.87 [0.85, 0.89]
Beaton (2016)	44	50	9	286	Non_expert	0.83 [0.70, 0.92]	0.85 [0.81, 0.89]
Mirabel (2015)	41	106	8	1062	Non_expert	0.84 [0.70, 0.93]	0.91 [0.89, 0.93]
Ploutz (2016)	32	194	11	719	Non_expert	0.74 [0.59, 0.86]	0.79 [0.76, 0.81]
Zuhlke (2016)	21	0	5	62	Expert	0.81 [0.61, 0.93]	1.00 [0.94, 1.00]

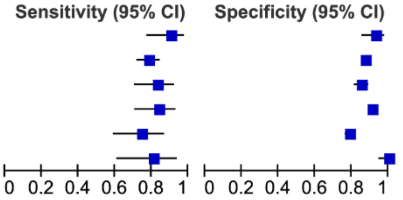

Fig 1. Forest plot of sensitivity and specificity of handheld echocardiography for any RHD

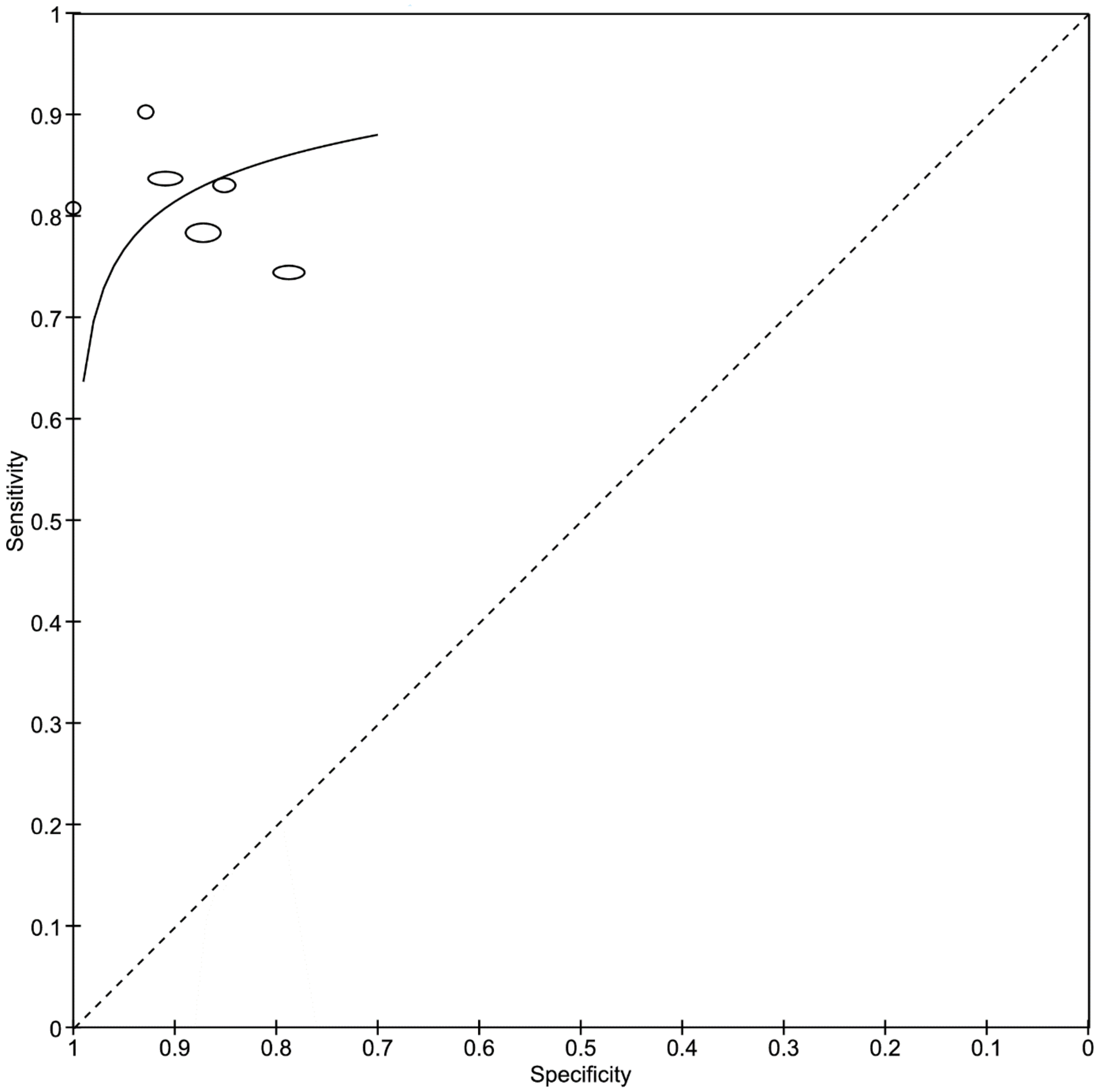

Fig 2. Summary ROC plot of sensitivity versus specificity of handheld echocardiography for any RHD

Definite RHD:

Study	TP	FP	FN	TN	Sensitivity (95% CI)	Specificity (95% CI)
Beaton (2014)	25	4	16	80	0.61 [0.45, 0.76]	0.95 [0.88, 0.99]
Beaton (2015)	46	158	1	1076	0.98 [0.89, 1.00]	0.87 [0.85, 0.89]
Mirabel (2015)	14	106	1	1062	0.93 [0.68, 1.00]	0.91 [0.89, 0.93]
Ploutz (2016)	10	194	1	719	0.91 [0.59, 1.00]	0.79 [0.76, 0.81]
Zuhlke (2016)	12	0	1	62	0.92 [0.64, 1.00]	1.00 [0.94, 1.00]

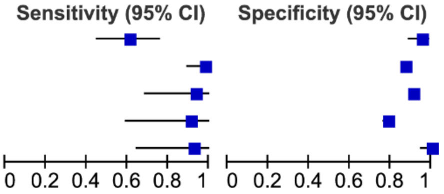

Fig 3. Forest plot of sensitivity and specificity of handheld echocardiography for definite RHD

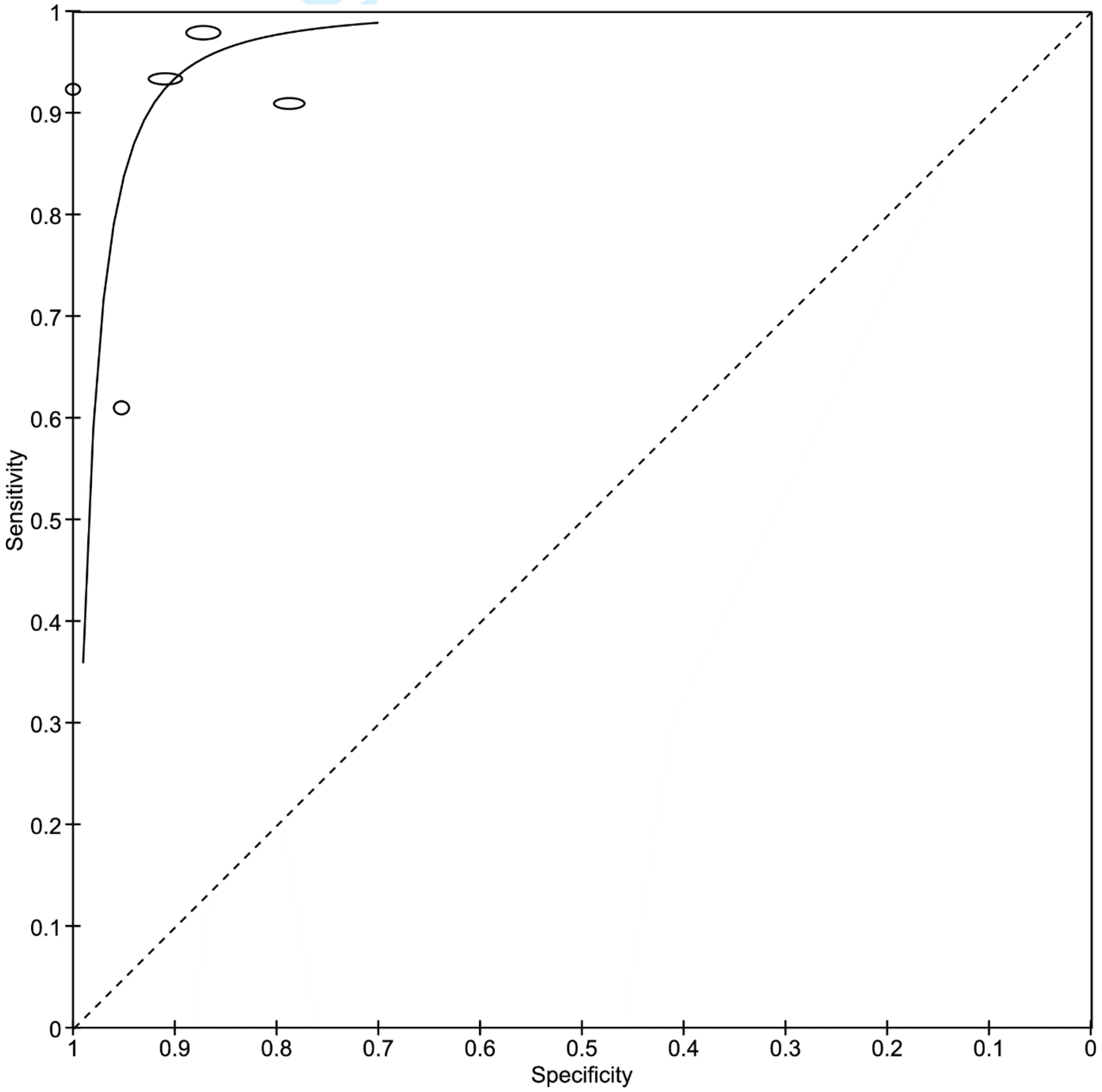

Fig 4. Summary ROC plot of sensitivity versus specificity of handheld echocardiography for definite RHD

Borderline RHD:

Study	TP	FP	FN	TN	Sensitivity (95% CI)	Specificity (95% CI)
Beaton (2014)	8	6	78	4	0.09 [0.04, 0.18]	0.40 [0.12, 0.74]
Beaton (2015)	95	158	38	1076	0.71 [0.63, 0.79]	0.87 [0.85, 0.89]
Mirabel (2015)	27	106	7	1062	0.79 [0.62, 0.91]	0.91 [0.89, 0.93]
Ploutz (2016)	22	194	10	719	0.69 [0.50, 0.84]	0.79 [0.76, 0.81]
Zuhlke (2016)	9	0	4	62	0.69 [0.39, 0.91]	1.00 [0.94, 1.00]

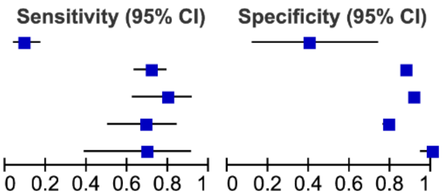

Fig 5. Forest plot of sensitivity and specificity of handheld echocardiography for borderline RHD

For

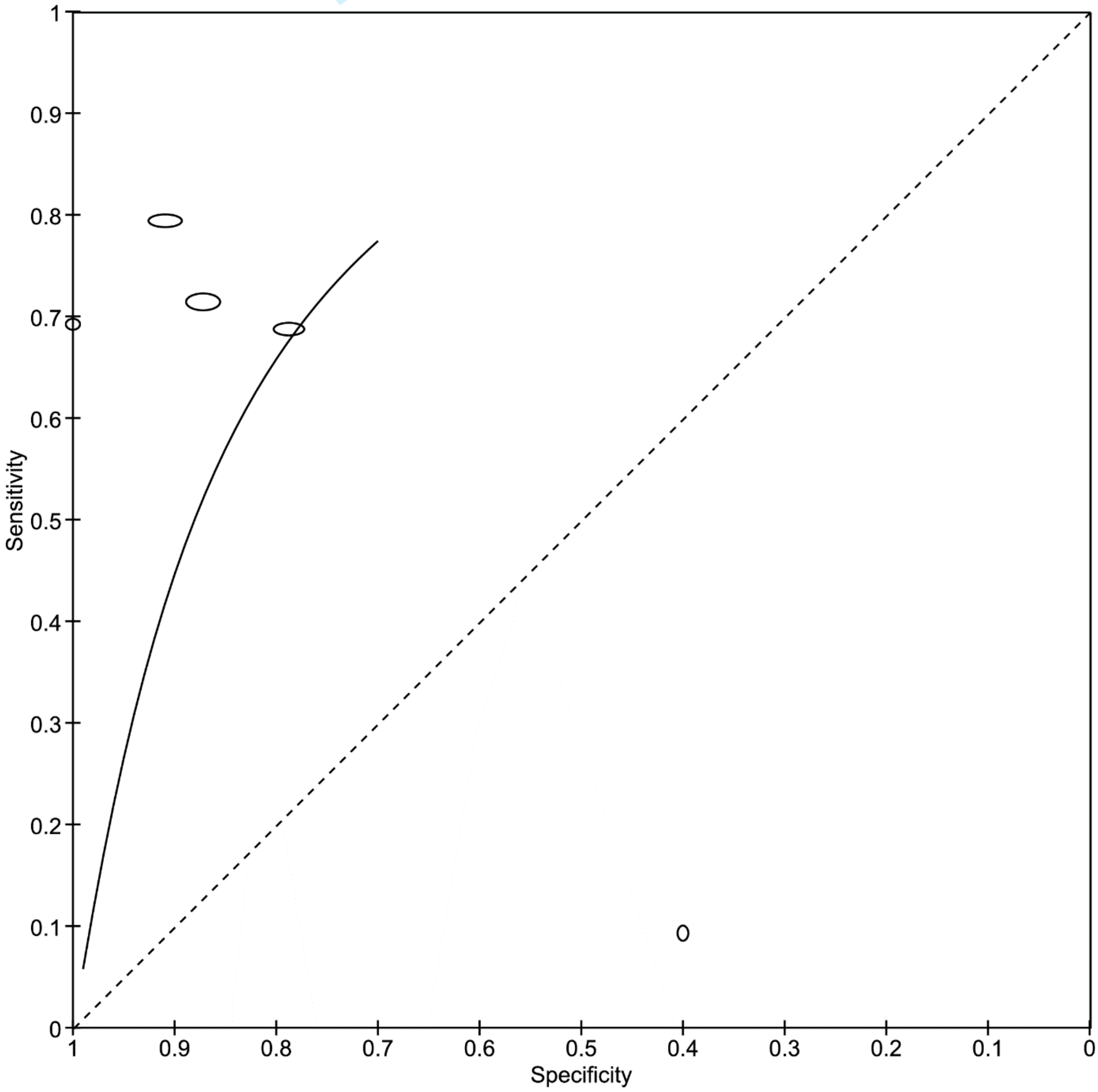

Fig 6. Summary ROC plot of sensitivity versus specificity of handheld echocardiography for borderline RHD

Supplementary file 3. HSROC plot for subgroup analysis of echocardiographer expertise.

Subgroup Analysis:

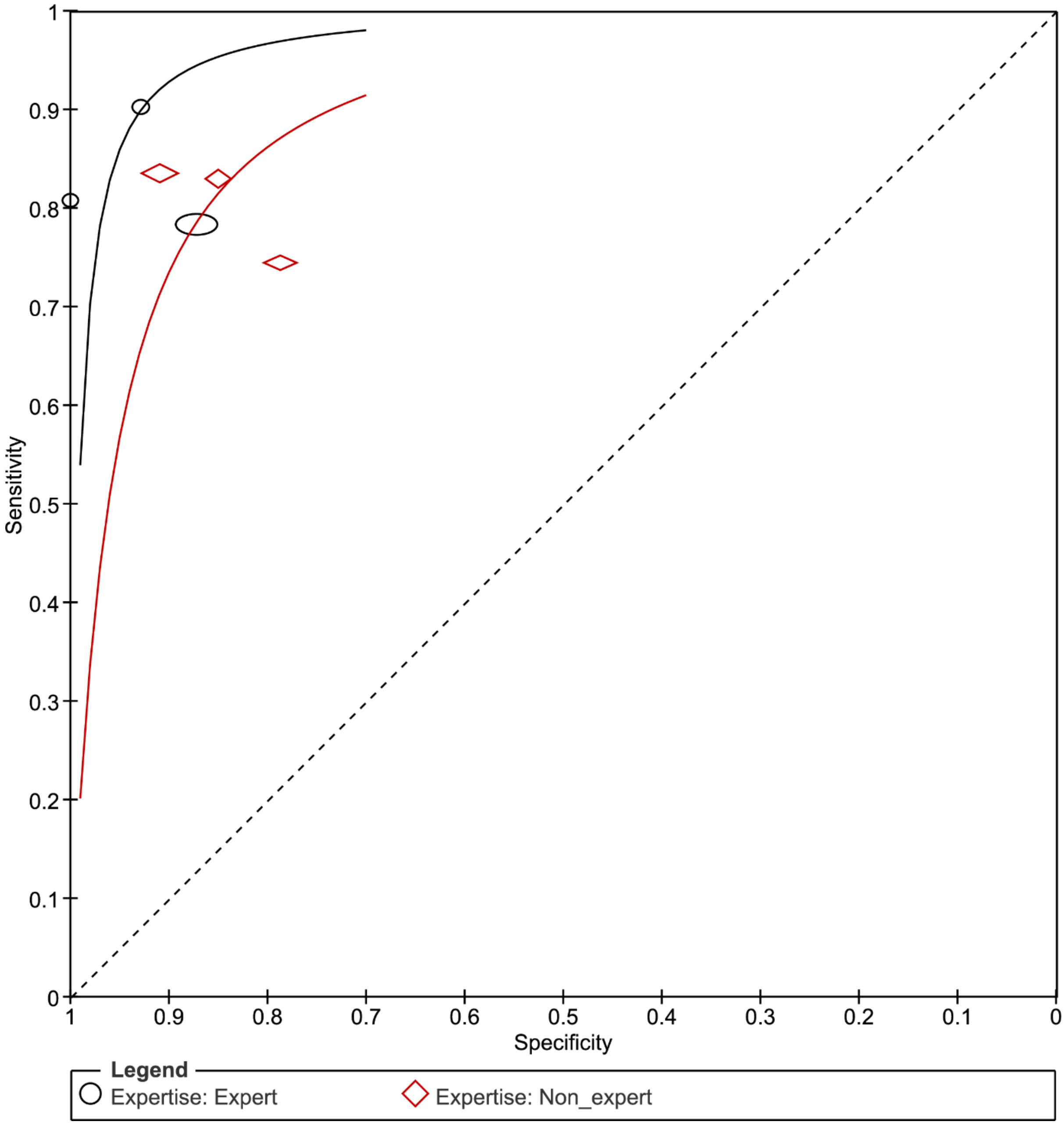

Fig 7. Summary ROC plot of sensitivity versus specificity of handheld echocardiography for any RHD according to echocardiographer expertise

PRISMA-DTA 2018 Checklist

SECTION AND TOPIC	ITEM NO.	DESCRIPTION	REPORTED ON PAGE #
**TITLE**			
Title	1	Identify the report as a systematic review (meta-analysis) of DTA studies.	1
**ABSTRACT**			
Abstract	2	Provide a structured summary including, as applicable: background; objectives; data sources; study eligibility criteria, participants, and interventions; study appraisal and synthesis methods; results; limitations; conclusions and implications of key findings; systematic review registration number.	3 - 4
**INTRODUCTION**			
Rationale	3	Describe the rationale for the review in the context of what is already known.	5 - 6
Clinical role of index test	D1	State the scientific and clinical background, including the intended use and clinical role of the index test, and if applicable, the rationale for minimally acceptable test accuracy (or minimum difference in accuracy for a comparative design).	5
Objectives	4	Provide an explicit statement of questions being addressed in terms of participants, index test, and target conditions.	5
**METHODS**			
Protocol and registration	5	Indicate where the review protocol can be accessed (e.g. web address) and provide trial registration number if available.	6
Eligibility criteria	6	Specify study characteristics (participants, setting, index test, reference standards, target conditions, and study design) and report characteristics (e.g., years considered, language, publication status) used as criteria for eligibility and providing rationale.	6 - 7
Information sources	7	Describe all information sources (e.g. databases with dates of coverage, contact with study authors to identify additional studies) in the search and the date last searched.	6, 9 & 10
Search	8	Present full search strategies for all electronic databases and other sources searched, including any limits used so that they can be repeated.	See supplementary files
Study selection	9	State the process for selecting studies (e.g., screening, eligibility, whether included in systematic review, and, if applicable, included in the meta-analysis).	6 - 8
Data collection process	10	Describe methods of data extraction from reports (e.g., piloted forms, independently, in duplicate) and any processes for obtaining and confirming data from investigators.	7
Definitions for data extraction	11	Provide definitions used in data extraction and classifications of target conditions, index tests, reference standards, and other characteristics (e.g. study design, clinical setting).	See supplementary files for editors
Risk of bias and applicability	12	Describe methods used for assessing risk of bias in individual studies and concerns regarding the applicability to the review question.	8
Diagnostic accuracy measures	13	State the principal diagnostic accuracy measures reported (e.g. sensitivity, specificity) and state the unit of assessment (e.g. per patient vs per lesion).	7

For peer review only

SECTION AND TOPIC	ITEM NO.	DESCRIPTION	REPORTED ON PAGE #
Synthesis of results	14	Describe the methods of handling the data, combining the results of the studies and describing the variability between studies. This could include, but is not limited to (1) handling of multiple definitions of the target condition, (2) handling of multiple thresholds of test positivity, (3) handling multiple index test readers, (4) handling of indeterminate test results, (5) grouping and comparing tests, and (6) handling of different reference standards.	8 - 9
Meta-analysis	D2	Report statistical methods used for meta-analyses if performed.	8 - 9
Additional analyses	16	Describe the methods of the additional analyses (e.g. sensitivity or subgroup analyses, meta-regression), if done, indicating which were prespecified.	8 - 9
**RESULTS**			
Study selection	17	Provide the numbers of studies screened, assessed for eligibility, included in the review, and included in the meta-analysis if applicable, with reasons for exclusions at each stage, ideally with a flow diagram.	10 - 11
Study characteristics	18	For each included study, provide citations and present key characteristics including (1) participant characteristics (presentation, prior testing), (2) clinical setting, (3) study design, (4) target condition definition, (5) index test, (6) reference standard, (7) sample size, and (8) funding sources.	10 - 12
Risk of bias and applicability	19	Present evaluation of risk of bias and concerns regarding applicability for each study.	13
Results of individual studies	20	For each analysis in each study (e.g. unique combination of index test, reference standard, and positivity threshold), report 2 × 2 data (TP, FP, FN, TN) with estimates of diagnostic accuracy and confidence intervals, ideally with a forest plot or a receiver operating characteristic curve.	See Supplementary files
Synthesis of results	21	Describe test accuracy, including variability; if meta-analysis was done, include results and confidence intervals.	14 - 15
Additional analysis	23	Give results of additional analyses, if done (e.g. sensitivity or subgroup analyses, meta-regression, analysis of index test, failure rates, proportion of inconclusive results, and adverse events).	15 - 16
**DISCUSSION**			
Summary	24	Summarize the main findings including the strength of evidence.	17 - 19
Limitations	25	Discuss limitations from included studies (e.g. risk of bias and concerns regarding applicability) and from the review process (e.g., incomplete retrieval of identified research).	19 - 20
Conclusions	26	Provide a general interpretation of the results in the context of other evidence. Discuss implications for future research and clinical practice (e.g. the intended use and clinical role of the index test).	20 - 22
**FUNDING**			
Funding	27	For the systematic review, describe the sources of funding and other support and the role of the funders.	See funding statement

Abbreviations: FN, false negative; FP, false positive; TN, true negative; TP, true positive
From: McInnes MDF, Moher D, Thombs BD, McGrath TA, Bossuyt PM; and the PRISMA-DTA Group (2018). Preferred Reporting Items for a Systematic Review and Meta-analysis of Diagnostic Test Accuracy Studies: The PRISMA-DTA Statement. JAMA 319(4): 388 - 396. doi: 10.1001/jama.2017.19163.

For peer review only

The PRISMA for Abstracts Checklist

TITLE	CHECKLIST ITEM	REPORTED ON PAGE #
1. Title:	Identify the report as a systematic review, meta-analysis, or both.	1 and 3
**BACKGROUND**		
2. Objectives:	The research question including components such as participants, interventions, comparators, and outcomes.	3
**METHODS**		
3. Eligibility criteria:	Study and report characteristics used as criteria for inclusion.	3
4. Information sources:	Key databases searched and search dates.	3
5. Risk of bias:	Methods of assessing risk of bias.	3
**RESULTS**		
6. Included studies:	Number and type of included studies and participants and relevant characteristics of studies.	3
7. Synthesis of results:	Results for main outcomes (benefits and harms), preferably indicating the number of studies and participants for each. If meta-analysis was done, include summary measures and confidence intervals.	3-4
8. Description of the effect:	Direction of the effect (i.e. which group is favoured) and size of the effect in terms meaningful to clinicians and patients.	N/A
**DISCUSSION**		
9. Strengths and Limitations of evidence:	Brief summary of strengths and limitations of evidence (e.g. inconsistency, imprecision, indirectness, or risk of bias, other supporting or conflicting evidence)	4
10. Interpretation:	General interpretation of the results and important implications	4
**OTHER**		
11. Funding:	Primary source of funding for the review.	N/A
12. Registration:	Registration number and registry name.	4

BMJ Open

Standard echocardiography versus handheld echocardiography for the detection of subclinical rheumatic heart disease: A systematic review and meta-analysis of diagnostic accuracy

Journal:	*BMJ Open*
Manuscript ID	bmjopen-2020-038449.R2
Article Type:	Original research
Date Submitted by the Author:	24-Aug-2020

Complete List of Authors:	Telford, Lisa; University of Cape Town Faculty of Health Sciences, Department of Medicine, Groote Schuur Hospital; University of Cape Town Faculty of Health Sciences, Department of Paediatrics and Child Health, Red Cross War Memorial Children's Hospital<br>Abdullahi, Leila; University of Cape Town Faculty of Health Sciences, Department of Paediatrics and Child Health, Red Cross War Memorial Children's Hospital; African Institute for Development Policy (AFIDEP)<br>Ochodo, Eleanor; University of Stellenbosch Centre for Evidence-Based Health Care, Department of Global Health, Faculty of Medicine and Health Sciences<br>ZUHLKE, LIESL; University of Cape Town Faculty of Health Sciences, Division of Cardiology, Department of Paediatrics and Child Health, Red Cross War Memorial Children's Hospital; University of Cape Town Faculty of Health Sciences, Division of Cardiology, Department of Medicine, Groote Schuur Hospital<br>Engel, Mark; University of Cape Town Faculty of Health Sciences, Department of Medicine, Groote Schuur Hospital

**Primary Subject Heading**:	Diagnostics
Secondary Subject Heading:	Evidence based practice, Public health, Cardiovascular medicine
Keywords:	Echocardiography < CARDIOLOGY, Paediatric cardiology < CARDIOLOGY, Valvular heart disease < CARDIOLOGY, PUBLIC HEALTH, Cardiovascular imaging < RADIOLOGY & IMAGING

I, the Submitting Author has the right to grant and does grant on behalf of all authors of the Work (as defined in the below author licence), an exclusive licence and/or a non-exclusive licence for contributions from authors who are: i) UK Crown employees; ii) where BMJ has agreed a CC-BY licence shall apply, and/or iii) in accordance with the terms applicable for US Federal Government officers or employees acting as part of their official duties; on a worldwide, perpetual, irrevocable, royalty-free basis to BMJ Publishing Group Ltd ("BMJ") its licensees and where the relevant Journal is co-owned by BMJ to the co-owners of the Journal, to publish the Work in this journal and any other BMJ products and to exploit all rights, as set out in our licence.

The Submitting Author accepts and understands that any supply made under these terms is made by BMJ to the Submitting Author unless you are acting as an employee on behalf of your employer or a postgraduate student of an affiliated institution which is paying any applicable article publishing charge ("APC") for Open Access articles. Where the Submitting Author wishes to make the Work available on an Open Access basis (and intends to pay the relevant APC), the terms of reuse of such Open Access shall be governed by a Creative Commons licence – details of these licences and which Creative Commons licence will apply to this Work are set out in our licence referred to above.

Other than as permitted in any relevant BMJ Author's Self Archiving Policies, I confirm this Work has not been accepted for publication elsewhere, is not being considered for publication elsewhere and does not duplicate material already published. I confirm all authors consent to publication of this Work and authorise the granting of this licence.

For peer review only

Standard echocardiography versus handheld echocardiography for the detection of subclinical rheumatic heart disease: A systematic review and meta-analysis of diagnostic accuracy

Lisa H Telford[1,2], Leila H Abdullahi[2,3], Eleanor A Ochodo[4], Liesl J Zühlke[5,6], Mark E Engel[1]

Lisa H Telford

[1]Department of Medicine, Groote Schuur Hospital and University of Cape Town, Cape Town, South Africa.

[2]Department of Paediatrics and Child Health, Red Cross War Memorial Children's Hospital and University of Cape Town, Cape Town, South Africa.

Email: lisa.telford@uct.ac.za

ORCID ID: https://orcid.org/0000-0002-8554-057X

Leila H Abdullahi

[2]Department of Paediatrics and Child Health, Red Cross War Memorial Children's Hospital and University of Cape Town, Cape Town, South Africa.

[3]African Institute for Development Policy (AFIDEP), Nairobi, Kenya.

Email: leylaz@live.co.za

ORCID ID: None

Eleanor A Ochodo

[4]Centre for Evidence-based Health Care, Department of Global Health, Faculty of Medicine and Health Sciences, Stellenbosch University, Cape Town, South Africa.

Email: eochodo@sun.ac.za

ORCID ID: https://orcid.org/0000-0002-7951-3030

For peer review only

Liesl J Zühlke

[5]Division of Cardiology, Department of Medicine, Groote Schuur Hospital and University of Cape Town, Cape Town, South Africa.

[6]Division of Cardiology, Department of Paediatrics and Child Health, Red Cross War Memorial Children's Hospital and University of Cape Town, Cape Town, South Africa.

Email: liesl.zuhlke@uct.ac.za

ORCID ID: https://orcid.org/0000-0003-3961-2760

Mark E Engel

[1]Department of Medicine, Groote Schuur Hospital and University of Cape Town, Cape Town, South Africa.

Email: mark.engel@uct.ac.za

ORCID ID: https://orcid.org/0000-0002-1334-882

Correspondence to: Ms Lisa H Telford

Room F3, F-floor, Red Cross War Memorial Children's Hospital, Main Hospital Building, Klipfontein Road Rondebosch 7700, South Africa

+27 21 650 2438

lisa.telford@uct.ac.za

Keywords: rheumatic heart disease, echocardiography, diagnostic accuracy, systematic review, meta-analysis

Word count: 3361

For peer review only

ABSTRACT

Objective: To summarise the accuracy of handheld echocardiography (HAND) which, if shown to be sufficiently similar to that of standard echocardiography (STAND), could usher in a new age of rheumatic heart disease (RHD) screening in endemic areas.

Design: Systematic review and meta-analysis.

Data sources: PubMed, Scopus, EBSCOHost and ISI Web of Science were searched for studies published from 2012 onwards.

Eligibility criteria: Studies assessing the accuracy of HAND compared to STAND when performed by an experienced cardiologist in conjunction with the 2012 World Heart Federation criteria among populations of children and adolescents living in endemic areas were included.

Data extraction and synthesis: Two reviewers independently extracted data and assessed the methodological quality of included studies against review specific QUADAS-2 criteria. A meta-analysis using the Hierarchical Summary Receiver Operating Characteristic model was conducted to produce summary results of sensitivity and specificity. Forest plots and scatter plots in Receiver Operating Characteristic space in combination with subgroup analyses were used to investigate heterogeneity. Publication bias was not investigated.

Results: Six studies (N=4208) were included in the analysis. For any RHD detection, the pooled results from six studies were; sensitivity: 81.6% (95% CI: 76.5%-86.6%) and specificity: 89.8% (84.5%-95.0%). Meta-analytical results from five of the six included studies

For peer review only

were; sensitivity: 91.1% (80.5%-100%) and specificity: 92.0% (85.6%-98.4%) for the detection of definite RHD only and sensitivity: 62.0% (31.8%-92.2%) and specificity: 82.3% (65.2%-99.5%) for the detection of borderline RHD only.

Conclusions: HAND displayed good accuracy for detecting definite RHD only and modest accuracy for detecting any RHD but demonstrated poor accuracy for the detection of borderline RHD alone. Findings from this review provide some evidence for the potential of HAND to increase access to echocardiographic screening for RHD in resource-limited and remote settings, however, further research into feasibility and cost-effectiveness of wide-scale screening is still needed.

PROSPERO registration number: PROSPERO CRD42016051261.

Strengths and limitations of this study:

- Language restrictions were not imposed during the literature search to minimise the chance of missing studies.
- Data extraction was performed by two independent reviewers thereby reducing the risk of bias.
- Insufficient reporting limited our ability to adequately assess risk of bias and investigate potential sources of heterogeneity.
- The small number of included studies prevented us from performing meta-regression.

For peer review only

INTRODUCTION

Rheumatic heart disease (RHD) is an acquired permanent heart valve condition which results from an atypical immune reaction to group A streptococcal (GAS) infection typically occurring in childhood.[1,2] Disease progression leading to chronic RHD can result in irreversible heart valve damage, cardiac failure, and premature death.[3,4] RHD is, however, a preventable and treatable chronic condition which most often effects disadvantaged populations.[3,5]

Significantly, RHD can remain asymptomatic for many years, particularly during the initial stages, thereby hindering the timely implementation of penicillin prophylaxis.[6] Echocardiographic screening to identify those with subclinical disease has been advocated as a means to support secondary prevention and potentially slow disease progression to overt clinical RHD.[7,8] Yet the feasibility of wide scale echocardiographic screening remains hindered by high costs and the scarcity of trained personnel.[9] Alternative RHD screening tests which are both accurate and affordable are therefore needed in many endemic areas.

Handheld echocardiography (HAND) is a non-invasive, highly portable and comparatively less expensive device which has been presented in recent publications to be a promising alternative to standard echocardiography (STAND), despite some limitations such as a lack of spectral Doppler capabilities.[10,11] For HAND to be considered a suitable replacement for STAND, the device's accuracy needs to be similar to that of STAND.

We conducted a systematic review and meta-analysis of studies assessing the diagnostic accuracy of HAND for the detection of RHD in children and adolescents. The findings of this

For peer review only

review may offer direction to guideline developers as well as assist with the identification of gaps in diagnostic testing for RHD in endemic areas.

METHODS

This systematic review was prepared according to the Preferred Reporting Items for a Systematic Review and Meta-Analysis of Diagnostic Test Accuracy Studies (PRISMA-DTA) guidelines.[12] The protocol for this review is registered with the International Prospective Register of Systematic Reviews (PROSPERO) under the registration number CRD42016051261 and has been published in BMJ Open.[13]

Patient and public involvement

No patient involved.

Data sources and study eligibility

Studies were considered eligible for inclusion if the following criteria were met: 1) the accuracy of HAND compared to STAND when performed by an experienced cardiologist and in conjunction with the 2012 World Heart Federation (WHF) criteria was evaluated, and 2) the sample consisted of populations of children and adolescents living in endemic areas. Only primary observational studies of either a cross-sectional, cohort or diagnostic case-control design were considered. Descriptive studies such case studies and case series were excluded as were studies reporting on the same data. Studies using non-handheld devices as the index test or criteria other than the 2012 WHF criteria in combination with STAND as the reference test were also excluded.

For peer review only

We conducted systematic electronic literature searches of four sources (PubMed, Scopus, EBSCOHost and ISI Web of Science) using predefined tailor-made strategies (see supplementary file 1). No restrictions in terms of language were applied, however, searches were limited to articles published from 2012 onwards. Both published and unpublished literature was considered eligible for inclusion. A manual search of the reference lists of all included studies as well as relevant review articles was also conducted.

The titles and/or abstracts of all identified articles were screened independently by two reviewers. During this process, and on the basis of predefined eligibility criteria, all clearly ineligible studies were excluded. Discrepancies regarding eligibility were resolved through discussion and consensus. Some authors were contacted for additional information on published data.

Data extraction and management

Using a predefined data extraction form, two reviewers independently extracted information on metrics of diagnostic accuracy: numbers of true positives (TP), false positives (FP), true negatives (TN) and false negatives (FN) as well as other covariates relating to study characteristics, population, reference and index test details, test outcome and number of missing or unavailable test results from all included studies.

Accuracy measures: sensitivity and specificity were calculated using the numbers of TP, FP, TN and FN in accordance with standard convention. Data extraction conflicts were resolved through discussion and with the assistance of a third reviewer where necessary. Information garnered through the data extraction process was used to determine each study’s quality as well as for synthesising evidence.

For peer review only - http://bmjopen.bmj.com/site/about/guidelines.xhtml

Assessment of methodological quality

A review-specific Quality Assessment of Diagnostic Accuracy Studies (QUADAS-2) tool was used to assess the risk of bias and concerns regarding applicability of all included studies.[14] The tool, encompassing four domains, was tailored to meet the specific requirements of this review. Two reviewers independently assessed the risk of bias in all included studies according to review-specific QUADAS-2 criteria. Discrepancies were resolved through discussion until consensus was reached and the assistance of a third reviewer was enlisted when necessary.

Statistical analysis and data synthesis

A meta-analysis using the Hierarchical Summary Receiver Operating Characteristic (HSROC) model was conducted to produce summary results of sensitivity and specificity. The HSROC model was used for meta-analysis as it accounts for variations in test thresholds.

Statistical measures of variability or heterogeneity to determine whether or not to conduct a meta-analysis were not used. While the Cochrane Q test and I^2 are routinely used to examine and quantify the amount of variability in meta-analyses of intervention studies where there is a single measure of effect or univariate outcome such as a risk or odds ratio,[15][16] meta-analyses of diagnostic test accuracy generally do not employ such measures.[17] Instead, forest plots and scatter plots in Receiver Operating Characteristic (ROC) space in combination with subgroup analyses were used to investigate heterogeneity.

Data were analysed according to three categorisations of RHD; any RHD (definite or borderline), definite RHD only and borderline RHD only. The any RHD category was selected

For peer review only

as the main meta-analysis as it had the most complete data. We were unable to extract metrics of diagnostic accuracy for the definite and borderline RHD only categories from Beaton, 2016 and therefore excluded this study from these meta-analyses. We chose to use Nurse A's results for Mirabel, 2015 since Nurse A and Nurse B both interpreted the same HAND images which prevented the pooling of data.

Data from Zühlke, 2016 were included in the analysis and synthesis of data even though the age range of participants fell outside the predefined range for eligibility. It was determined that this study should be included, regardless, since the data overall were quite few and the variation in age was not significant enough to warrant exclusion. However, data from Zühlke, 2016 were excluded from all summary estimates of disease prevalence since this study used a nested case-control design which predetermines disease prevalence by design.

Heterogeneity was examined for the main meta-analysis only. We were only able to investigate the relationship between test accuracy and echocardiographer expertise through subgroup analysis. A sensitivity analysis was performed instead of subgroup analysis for the categorical covariates; HAND protocol and geographic location due to the skewed distribution of studies within each subgroup. We were unable to perform meta-regression for the covariates; age and sex due to insufficient and inadequately reported data.[17] We were also unable to conduct a sensitivity analysis on risk of bias since no studies were found to have a high risk of bias. All plots were generated using the Review Manager (RevMan) software package, version 5.3.[18] Meta-analysis was performed using SAS® software, version 9.4.[19]

We did not investigate publication bias as methods of assessing publication bias for studies of diagnostic accuracy are still being developed. While the Deeks test has been suggested

For peer review only

for use in diagnostic accuracy studies, the test has low power for detecting asymmetry in funnel plots, particularly when a large amount of heterogeneity is present.[17]

RESULTS

Results of the search

Results of the literature search are reported in accordance with the PRISMA Statement and the study selection process is illustrated in figure 1.[20] All electronic searches were performed by two independent reviewers on September 27th, 2017. Combined, the search yielded a total of ninety-two records, of which nine were duplicates. A total of sixty-seven were excluded based on title or abstract leaving sixteen for full-text review. Nine studies were excluded on the basis of a full-text review whilst an additional study was also excluded after consultation with study authors. Six studies which met the predefined eligibility criteria were included in this review.

Figure 1. PRISMA flow diagram illustrating study identification, selection, eligibility and inclusion. *From* Moher et al.[20]

The same search was re-run on March 3rd, 2020 to check for any additional eligible studies. Only one potentially eligible study [21,22] was found but has been excluded on the basis of being an abstract only publication with no full-text available for review.

Included studies

A summary of notable characteristics of all included studies [23–28] is shown in table 1. One study did not avoid a case-control design, however, cases and controls were sampled from the same population. Research has shown that case-control studies which use alternative

For peer review only

diagnosis controls, controls from non-endemic areas or confirmed disease-free (healthy) controls tend to overestimate specificity.[29]

Significantly, all but two studies were conducted in Africa. Screening was performed in RHD endemic areas among children and adolescents with most studies being school based. Combined, all six studies included a total of 4208 participants of which 54% were female. The pooled mean age of participant's was 10.8 years (SD: ±1.9).

All included studies used the same make of handheld device; the Vscan machine (General Electric, Medical Systems, Milwaukee, Wisconsin, USA) paired with a 1.7 to 3.4 MHz transducer. These machines provide both two-dimensional (2D) and colour imaging on an integrated 8.9cm display.[24,26] Frame rates range from 25 to 30 Hz for greyscale imaging and 12 to 16 Hz for colour Doppler.[23,27] Vscan machines are, however, limited by a lack of spectral Doppler capabilities.[24]

For peer review only

Table 1. Summary of characteristics of included studies: ordered alphabetically by study author.

**Study**	**Study Design**	**City, Country** (Classification¤)	**Recruitment Site/Setting**	**Participant Selection Method**	**Sample Size** (N)	**% Female**	**Mean Age** (years)	**SD**
**Beaton, 2014**[23]	Spiked cohort	Kampala, Uganda (Low-income)	A School and the Mulago Hospital Complex	Unclear	125	55.2	10.8¥	-
**Beaton, 2015**[24]	Cross-sectional	Gulu, Uganda (Low-income)	5 public schools	A random 10% subset of the entire sample plus any child with mitral or aortic regurgitation were preselected to receive HAND	1420	53	10.8	±2.6
**Beaton, 2016**[25]	Cross-sectional	Belo Horizonte, Brazil (Upper-middle income)	2 primary and 3 secondary public schools	A subset of the sample containing all STAND abnormals plus a random 25% of all STAND normals were preselected for HAND	397	49.1	13.9	±2.6
**Mirabel, 2015**[26]	Cross-sectional	Nouméa, New Caledonia (High-income)	Primary schools	Consecutive	1217	50.5	9.6	±0.5
**Ploutz, 2016**[27]	Cross-sectional	Gulu, Uganda (Low-income)	2 primary schools	Consecutive	956	60.7	11.1	±2.5
**Zühlke, 2016**[28]	Nested case-control	Cape Town, South Africa (Upper-middle income)	Schools	Unclear	93	68.8	17¥†	-
**Total**					4208	54	10.8	±1.9

¤According to the World Bank's economic classification system; ¥excluded from pooled mean & SD calculations (incomplete or incomparable data); †median age (mean age not available)

Excluded studies

Ten studies [9,10,30–37] were excluded during full-text screening. Reasons for exclusion included abstract only publications [32,33,35], the use of ineligible reference [30,31] or index [34] tests, the use of duplicate data [10,37], not specifying the test threshold a-priori [9], and not being a study of diagnostic accuracy [36].

Methodological quality of included studies

A summary of the assessment of methodological quality of all included studies is illustrated in figure 2. Overall, only two [25,26] of the six [23–28] included studies were assessed as having a low risk of bias while the risk in the remaining four [23,24,27,28] was unclear. Two studies [23,28] had participant selection bias concerns. Of these both [23,28] failed to adequately describe participant enrolment methods whilst one [28] also did not avoid a case-control design. The risk of bias in terms of flow and timing was unclear in two studies [24,27]. Of these, one [27] did not include all participants in the analysis due to technical difficulties while the time interval between the index and reference test was unclear in the other [24]. Overall, time intervals between index and reference tests were poorly described. Likewise, reporting of quality control of the index test was uniformly poor across all included studies. Concerns regarding applicability were low in all six [23–28] studies.

Figure 2. Risk of bias and applicability concerns graph: review authors' judgments about each domain presented as percentages across included studies.

Findings

For peer review only

For any RHD

A total of six evaluations of HAND for any RHD were performed with data from six studies and a total of 4208 participants. Pooled prevalence of any RHD from five included studies was 12% (95% confidence interval (CI): 6%-19%). The forest plot revealed little variation in estimates of sensitivity and specificity and the HSROC plot (see supplementary file 2 for all plots) revealed moderate accuracy of the test. Meta-analytical sensitivity and specificity (95% CI) of data at mixed thresholds were 82% (77%-87%) and 90% (85%-95%) respectively.

For definite RHD

A total of five evaluations of HAND for definite RHD were performed with data from five studies and a total of 3588 participants. Pooled prevalence of definite RHD from four included studies was 6% (95% CI: 2%-12%). The forest plot revealed some variation in estimates of specificity while estimates of sensitivity were largely homogenous with the exception of a single outlier. The HSROC plot indicated good accuracy of the test. Meta-analytical sensitivity and specificity (95% CI) of data at mixed thresholds were 91% (81%-100%) and 92% (86%-98%) respectively.

For borderline RHD

A total of five evaluations of HAND for borderline RHD were performed with data from five studies and a total of 3685 participants. Pooled prevalence of borderline RHD from four included studies was 20% (95% CI: 6%-39%). The forest plot revealed some variation in estimates of specificity while estimates of sensitivity were largely homogenous with the exception of a single outlier. The HSROC plot indicated poor accuracy of the test. Meta-

For peer review only

analytical sensitivity and specificity (95% CI) of data at mixed thresholds were 62% (32%-92%) and 82% (65%-100%) respectively.

Investigations of heterogeneity

Heterogeneity or variation between studies was investigated both visually as well as through subgroup analysis for the main meta-analysis only. We were only able to perform this analysis for the any RHD category as the data were too few to enable model convergence for the definite and borderline RHD only categories.

Co-variates in the models

We were only able to use one of the five pre-specified covariates to investigate heterogeneity due to insufficient and inadequately reported data. HAND echocardiographer expertise (expert vs non-expert) was investigated as a possible source of heterogeneity through subgroup analysis. Half of all included studies evaluated the accuracy of HAND when performed and interpreted by trained non-experts while the other half assessed its accuracy in the hands of experts.

Subgroup and sensitivity analyses

A subgroup analysis was performed to investigate variations in echocardiographer expertise as a potential source of heterogeneity. Since no studies were found to have a high risk of bias we did not explore the effect of excluding such studies on the accuracy of summary estimates**.** Sensitivity analyses were, however, conducted to investigate the effect of removing a single study on summary estimates of sensitivity and specificity for the covariates; geographic location and HAND protocol.

For peer review only

A subgroup analysis for the covariate; echocardiographer expertise, as shown in table 2 and illustrated in supplementary file 3, revealed that both sensitivity (82.54% vs 80.76%) and specificity (94.57% vs 85.71%) were higher for any RHD detection using HAND when tests were performed and interpreted by experts compared to non-experts.

Sensitivity analyses (see table 2) were performed to investigate the effect of excluding a) the single high-income country study and b) the study which employed a single view protocol on the accuracy of summary estimates. We found that both sensitivity (81.17% vs 80.4%) and specificity (90.07% vs 87.15%) increased compared to the overall analysis when only low- and middle-income country studies were considered whereas both sensitivity (80.98% vs 85.35%) and specificity (87.46% vs 88.8%) decreased in comparison with the overall analysis when only studies which employed multiple view protocols were considered.

Table 2. Sources of heterogeneity for handheld echocardiography for any RHD.

Group	Covariate	Subgroup	n (N=6)	Median Pooled Sensitivity (95% Crl)	Median Pooled Specificity (95% Crl)
**Overall**			6	**81.56% (76.52-86.61)**	**89.75% (84.48-95.01)**
**Subgroup Analysis**	HAND Interpreter Expertise	Expert	3	82.54% (74.71-90.37)	94.57% (87.52-100)
		Non-expert	3	80.76% (73.18-88.33)	85.71% (79.91-91.51)
**Sensitivity Analysis**	Geographic Location	Overall		80.40% (64.96-95.84)	87.15% (76.35-97.96)
		Low- and middle-income countries	5	81.17% (75.63-86.71)	90.07% (82.98-97.15)
	HAND Protocol	Overall		85.35% (80.60-90.09)	88.80% (84.55-93.06)
		Multiple views	5	80.98% (75.38-86.59)	87.46% (83.24-91.67)

Abbreviations: HAND, handheld echocardiography; Crl, credible interval; n, number of studies; N, total number of included studies

DISCUSSION

Summary of main findings

We evaluated the accuracy of HAND for three distinct disease categories and found that, overall, the test was both sensitive and specific for detecting definite RHD only and moderately accurate for detecting any RHD but demonstrated insufficient accuracy for detecting borderline RHD alone.

Findings from this review provide some evidence for the potential of HAND to increase access to echocardiographic screening for RHD in resource-limited and remote settings. A summary of the accuracy estimates produced by meta-analysis using the HSROC method is included in table 3.

For peer review only

Table 3. Summary of findings.

What is the diagnostic accuracy of handheld echocardiography in detecting any RHD (definite or borderline)?		
**Patients/Population**	People residing in areas endemic for RHD (6 out of 6 studies)	
**Prior testing with echo**	Yes (2 studies), No (4 studies)	
**Settings**	5 out of 6 screening studies were field setting (communities and schools) based while 1 study was half hospital registry follow-up, half school based.<br>4 of the 6 studies were conducted in Africa with 3 of those from Uganda.	
**Index test(s)**	General Electric (GE) Vscan handheld machine (6 out of 6 studies)	
**Reference standard**	Standard echocardiography (2D, continuous-wave, and colour-Doppler echocardiography) performed by an experienced imager and in conjunction with the 2012 WHF criteria (6 out of 6 studies).<br>Test brands included; GE Vivid-I ultrasound machine (2 studies)<br>; GE Vivid-Q ultrasound machine (2 studies)<br>; Philips CX-50 ultrasound machine (1 study)<br>; Either a GE Vivid-I or Q or Philips CX-50 ultrasound machine (1 study)	
**Importance**	HAND is being used as first line replacement for STAND in disease screening programmes for RHD, as it is comparably inexpensive, quick, user friendly, easy to interpret, and may have similar sensitivity to STAND.	
**Studies**	Cross-sectional (n=4), spiked cohort (n=1) and nested case-control (n=1) studies. More than half (n=4) of all included studies did not explicitly state the study design used and were thus assigned a study design based on other reported characteristics and participant enrolment methods used.	
**Quality concerns**	Poor reporting of study design, participant characteristics and pre-test probability were common concerns. For the majority of studies the risk of bias was unclear in terms of 'patient selection' and 'flow and timing'. Concerns regarding applicability were low in all included studies.	
**Test types**	**Number of participants*** (N)	**Summary estimates** (95% credible CI)
HAND for **any RHD**	4208 (6)	Sensitivity; 81.56% (76.52-86.61)<br>Specificity; 89.75% (84.48-95.01)
HAND for **definite RHD**	3588 (5)	Sensitivity; 91.06% (80.46-100)<br>Specificity; 91.96% (85.57-98.36)

HAND for **borderline** **RHD**	3685 (5)	Sensitivity; 62.01% (31.8-92.22)<br>Specificity; 82.33% (65.15-99.52)
Abbreviations: CI, confidence interval; HAND, handheld echocardiography; N, number of studies; RHD, rheumatic heart disease; STAND, standard echocardiography *Excluding participants with other diagnoses on STAND		

For peer review only

Strengths and limitations of this review

Strengths

We have evaluated and summarised the accuracy of HAND for the detection of RHD in endemic areas, making the review relevant to current global agendas. This review also serves to highlight the existing gaps in evidence for which further research could be beneficial. We did not impose any limits in terms of language during the literature search so as to minimise the chance of missing studies. Data extraction was performed by two independent reviewers thereby reducing the risk of bias.

Limitations

There were a number of shortcomings of this review, which included;

Eligibility: We were unable to include studies which used STAND in conjunction with criteria other than the 2012 WHF criteria as the reference standard which limited the number of studies eligible for inclusion.

Quality of included studies: Insufficient reporting of participant characteristics and study methods including study design, participant selection and test timing restricted our ability to adequately assess risk of bias and investigate potential sources of heterogeneity.

Paucity of data: Insufficient and inadequately reported data as well as the presentation of aggregate data limited the scope of our investigations of heterogeneity while the small number of included studies prevented us from performing meta-regression. Overall, the findings from this review may lack power due to the small sample size.

For peer review only

Applicability of findings to the review question

Concerns regarding the applicability of included studies to the review question were considered low according to review-specific QUADAS-2 criteria. Since all but one were conducted in low- or middle-income countries, and all studies with one exception were conducted in field settings, the results of this review are applicable for use in endemic areas for which screening programmes are frequently targeted. However, our limited assessment of risk of bias and investigations into sources of heterogeneity such as age and gender due to insufficient reporting may lessen the applicability of findings to the review question.

In the context of disease control programmes, being able to demonstrate variation in test accuracy associated with factors such as age and gender would be beneficial for policy makers. Fully understanding included studies' risk of bias would also assist in objectively assessing the strength of evidence. For these reasons, prospective authors of diagnostic test accuracy studies are urged to make use of the Standards for Reporting of Diagnostic Accuracy Studies (STARD) guidelines [38] when reporting methods of study design and conduct.

CONCLUSION

This review provides a summary of the accuracy of HAND for the detection of RHD. In populations of children and adolescents living in RHD endemic areas, HAND is both sensitive and specific for detecting definite RHD. The device is less accurate in detecting any RHD and demonstrates substandard accuracy for the detection of borderline RHD only. Nonetheless, this test may hold value as a replacement for first line screening due to its high sensitivity for definite RHD detection and adequate accuracy for any RHD detection.

For peer review only

Implications for practice

We have summarised the accuracy of HAND when used as a screening tool, however, the device’s potential value in terms of diagnostics has yet to be established. We therefore posit that HAND could be recommended as an acceptable replacement test for first line screening in endemic areas provided a standardised set of device-specific diagnostic criteria are developed.

Another key consideration is the applicability of these findings for recommendations to integrate screening into routine clinical practice. A recent publication has reviewed the cost-effectiveness of screening in high-risk populations [39] and determined that screening all indigenous Australian 5 to 12 year-olds in half of their communities in alternate years was found to be cost-effective, if RHD can be detected at least 2 years earlier. However, this result was sensitive to a number of assumptions, including local costs and context. Other cost-effectiveness models have also suggested modestly improved outcomes at lower cost.[40] Neither of these studies included the significant cost-reduction using HAND instead of STAND, hence, we highly recommend adding a cost-effectiveness analysis into proposed new screening studies.

Finally, our findings demonstrate comparable results by non-experts, this has also been demonstrated in several other reports [31,41], but again there are no detailed cost-effectiveness analyses using non-experts and HAND.

Implications for research

The findings of this review highlight the need for a new set of evidence-based guidelines tailored to the capabilities of HAND in order to maximise the device’s diagnostic potential.

For peer review only

Further studies assessing the diagnostic accuracy of HAND when using a standardised protocol are needed as is further research into the feasibility, cost-effectiveness and consequences of implementing wide scale screening programs. Furthermore, the development of standardised training programs for non-experts is recommended as screening for RHD in endemic areas inevitably rests on the success of task shifting.[27] We conclude that while HAND has been shown to be sufficiently accurate for the detection of RHD there is still a need for further research before its' wide scale use can be endorsed.

Author affiliations

[1]Department of Medicine, Groote Schuur Hospital and University of Cape Town, Cape Town, South Africa.

[2]Department of Paediatrics and Child Health, Red Cross War Memorial Children's Hospital and University of Cape Town, Cape Town, South Africa.

[3]African Institute for Development Policy (AFIDEP), Nairobi, Kenya.

[4]Centre for Evidence-based Health Care, Department of Global Health, Faculty of Medicine and Health Sciences, Stellenbosch University, Cape Town, South Africa.

[5]Division of Cardiology, Department of Medicine, Groote Schuur Hospital and University of Cape Town, Cape Town, South Africa.

[6]Division of Cardiology, Department of Paediatrics and Child Health, Red Cross War Memorial Children's Hospital and University of Cape Town, Cape Town, South Africa.

Author contributions

LJZ and MEE jointly conceived of the study and provided content advice. LHT and LHA performed the systematic review of the literature, independently extracted the data and are

For peer review only

responsible for the quality control of study selection. EAO provided methodological advice and was responsible for conducting the meta-analysis. LHT significantly contributed to the interpretation of the data and wrote the first draft of the manuscript. All authors contributed to editing subsequent versions of the manuscript.

Funding

LJZ, LHT and LHA all receive funding from the Medtronic Foundation through support to RHD Action. LHT receives support from the National Research Foundation of South Africa (NRFSA) through its internship programme. LJZ is supported by both the NRFSA and Medical Research Council (MRC).

For peer review only

Competing interests

None declared.

Patient consent for publication

Not required.

Provenance and peer review

Not commissioned; externally peer reviewed.

Data availability statement

Dataset, https://doi.org/10.25375/uct.11902077.v1, available from the ZivaHub repository. All other relevant data are included in the article or uploaded as supplementary information.

ORCID iDs

Lisa H Telford https://orcid.org/0000-0002-8554-057X

Eleanor A Ochodo https://orcid.org/0000-0002-7951-3030

Liesl J Zühlke https://orcid.org/0000-0003-3961-2760

Mark E Engel https://orcid.org/0000-0002-1334-882

For peer review only

References

1. Marijon E, Mirabel M, Celermajer DS, Jouven X. Rheumatic heart disease. *Lancet* [Internet] 2012;379(9819):953–64. Available from: https://doi.org/10.1016/S0140-6736(11)61171-9

2. Zühlke LJ, Beaton A, Engel ME, Hugo-Hamman CT, Karthikeyan G, Katzenellenbogen JM, et al. Group A Streptococcus, Acute Rheumatic Fever and Rheumatic Heart Disease: Epidemiology and Clinical Considerations. *Curr Treat Options Cardiovasc Med* [Internet] 2017;19(2):15. Available from: http://link.springer.com/10.1007/s11936-017-0513-y

3. World Heart Federation. Rheumatic Heart Disease: Neglected NCD of Poverty (Factsheet). Geneva: World Heart Federation; 2013.

4. Seckeler MD, Hoke T. The worldwide epidemiology of acute rheumatic fever and rheumatic heart disease. *Clin Epidemiol* [Internet] 2011;3(1):67–84. Available from: http://www.dovepress.com/the-worldwide-epidemiology-of-acute-rheumatic-fever-and-rheumatic-hear-peer-reviewed-article-CLEP

For peer review only - http://bmjopen.bmj.com/site/about/guidelines.xhtml

5. Dougherty S, Khorsandi M, Herbst P. Rheumatic heart disease screening: Current concepts and challenges. *Ann Pediatr Cardiol* [Internet] 2017;10(1):39. Available from: http://www.annalspc.com/text.asp?2017/10/1/39/197051

6. Moloi AH, Mall S, Engel ME, Stafford R, Zhu ZW, Zühlke LJ, et al. The Health Systems Barriers and Facilitators for RHD Prevalence: An Epidemiological Meta-Analysis From Uganda and Tanzania. *Glob Heart* [Internet] 2017;12(1):5–15. Available from: https://globalheartjournal.com/article/10.1016/j.gheart.2016.12.002/

7. Zühlke LJ, Watkins DA, Perkins S, Wyber R, Mwangi J, Markbreiter J, et al. A Comprehensive Needs Assessment Tool for Planning RHD Control Programs in Limited Resource Settings. *Glob Heart* [Internet] 2017;12(1):25–31. Available from: http://dx.doi.org/10.1016/j.gheart.2016.10.028

8. Palafox B, Mocumbi AO, Kumar RK, Ali SKM, Kennedy E, Haileamlak A, et al. The WHF Roadmap for Reducing CV Morbidity and Mortality Through Prevention and Control of RHD. *Glob Heart* [Internet] 2017;12(1):47–62. Available from: http://dx.doi.org/10.1016/j.gheart.2016.12.001

9. Lu JC, Sable C, Ensing GJ, Webb C, Scheel J, Aliku T, et al. Simplified rheumatic heart disease screening criteria for handheld echocardiography. *J Am Soc Echocardiogr* [Internet] 2015;28(4):463–9. Available from: http://dx.doi.org/10.1016/j.echo.2015.01.001

10. Godown J, Lu JC, Beaton A, Sable C, Mirembe G, Sanya R, et al. Handheld Echocardiography Versus Auscultation for Detection of Rheumatic Heart Disease. *Pediatrics* [Internet] 2015;135(4):e939–44. Available from:

For peer review only

http://search.ebscohost.com/login.aspx?direct=true&db=cmedm&AN=25780068&site=ehost-live

11. Saxena A. Rheumatic heart disease screening by “point-of-care” echocardiography: an acceptable alternative in resource limited settings? *Transl Pediatr* [Internet] 2015;4(3):210–3. Available from: http://www.ncbi.nlm.nih.gov/pubmed/26835377

12. McInnes MDF, Moher D, Thombs BD, McGrath TA, Bossuyt PM, and the PRISMA-DTA Group, et al. Preferred Reporting Items for a Systematic Review and Meta-analysis of Diagnostic Test Accuracy Studies: The PRISMA-DTA Statement. *JAMA* [Internet] 2018;319(4):388–96. Available from: http://jama.jamanetwork.com/article.aspx?doi=10.1001/jama.2017.19163

13. Telford LH, Abdullahi LH, Ochodo EA, Zühlke LJ, Engel ME. Standard echocardiography versus handheld echocardiography for the detection of subclinical rheumatic heart disease: protocol for a systematic review. *BMJ Open* [Internet] 2018;8(2):e020140. Available from: http://bmjopen.bmj.com/lookup/doi/10.1136/bmjopen-2017-020140

14. Whiting PF, Rutjes AWS, Westwood ME, Mallet S, Deeks JJ, Reitsma JB, et al. Research and Reporting Methods Accuracy Studies. *Ann Intern Med* 2011;155(4):529–36.

15. Higgins J, Thomas J, Chandler J, Cumpston M, Li T, Page M, et al., editors. Cochrane Handbook for Systematic Reviews of Interventions version 6.0 (updated July 2019) [Internet]. Cochrane; 2019. Available from: https://training.cochrane.org/handbook

16. Naaktgeboren CA, Ochodo EA, Van Enst WA, de Groot JAH, Hooft L, Leeflang MMG, et al. Assessing variability in results in systematic reviews of diagnostic studies. *BMC Med Res Methodol* [Internet] 2016;16(1):6. Available from:

For peer review only

http://dx.doi.org/10.1186/s12874-016-0108-4

17. Macaskill P, Gatsonis C, Deeks JJ, Harbord RM, Takwoingi Y. Chapter 10: Analysing and Presenting Results. In: Deeks JJ, Bossuyt PM, Gatsonis C, editors. Cochrane Handbook for Systematic Reviews of Diagnostic Test Accuracy Version 1 [Internet] The Cochrane Collaboration; 2010. Available from: https://methods.cochrane.org/sites/methods.cochrane.org.sdt/files/public/uploads/Chapter 10 - Version 1.0.pdf

18. Review Manager (RevMan). Copenhagen: The Nordic Cochrane Centre, The Cochrane Collaboration; 2014.

19. SAS/STAT Software. SAS Institute Inc.; 2011.

20. Moher D, Liberati A, Tetzlaff J, Altman DG, Altman DG, Antes G, et al. Preferred Reporting Items for Systematic Reviews and Meta-Analyses: The PRISMA Statement. *PLoS Med* [Internet] 2009;6(7):e1000097. Available from: http://www.ncbi.nlm.nih.gov/pubmed/19621072

21. Francis J, Fairhurst H, Kaethner A, Whalley G, Ryan C, Brown S, et al. Handheld PLAX View Echocardiography by Healthworkers for Detection of RHD. *Hear Lung Circ* [Internet] 2019;28:S51–2. Available from: https://linkinghub.elsevier.com/retrieve/pii/S1443950619305177

22. Francis J, Fairhurst H, Kaethner A, Whalley G, Ryan C, Dos Santos J, et al. P3133: Single parasternal long axis echocardiography by briefly trained health workers using handheld devices for detection of rheumatic heart disease: a prospective study of diagnostic accuracy. *Eur Heart J* [Internet] 2019;40(Supplement_1):3133. Available

For peer review only

from:
https://academic.oup.com/eurheartj/article/doi/10.1093/eurheartj/ehz745.0208/5597410

23. Beaton A, Aliku T, Okello E, Lubega S, McCarter R, Lwabi P, et al. The utility of handheld echocardiography for early diagnosis of rheumatic heart disease. *J Am Soc Echocardiogr* [Internet] 2014;27(1):42–9. Available from: http://dx.doi.org/10.1016/j.echo.2013.09.013

24. Beaton A, Lu JC, Aliku T, Dean P, Gaur L, Weinberg J, et al. The utility of handheld echocardiography for early rheumatic heart disease diagnosis: a field study. *Eur Hear J - Cardiovasc Imaging* [Internet] 2015;16(5):475–82. Available from: https://academic.oup.com/ehjcimaging/article-lookup/doi/10.1093/ehjci/jeu296

25. Beaton A, Nascimento BR, Diamantino AC, Pereira GTR, Lopes ELV, Miri CO, et al. Efficacy of a Standardized Computer-Based Training Curriculum to Teach Echocardiographic Identification of Rheumatic Heart Disease to Nonexpert Users. *Am J Cardiol* [Internet] 2016;117(11):1783–9. Available from: http://dx.doi.org/10.1016/j.amjcard.2016.03.006

26. Mirabel M, Bacquelin R, Tafflet M, Robillard C, Huon B, Corsenac P, et al. Screening for Rheumatic Heart Disease: Evaluation of a Focused Cardiac Ultrasound Approach. *Circ Cardiovasc Imaging* [Internet] 2015;8(1):e002324–e002324. Available from: http://www.ncbi.nlm.nih.gov/pubmed/25567654

27. Ploutz M, Lu JC, Scheel J, Webb C, Ensing GJ, Aliku T, et al. Handheld echocardiographic screening for rheumatic heart disease by non-experts. *Heart*

For peer review only

[Internet] 2016;102(1):35–9. Available from: http://10.0.4.112/heartjnl-2015-308236

28. Zühlke LJ, Engel ME, Nkepu S, Mayosi BM. Evaluation of a focussed protocol for hand-held echocardiography and computer-assisted auscultation in detecting latent rheumatic heart disease in scholars. *Cardiol Young* [Internet] 2016;26(06):1097–106. Available from: https://www.scopus.com/inward/record.uri?eid=2-s2.0-84943741614&partnerID=40&md5=816a5cc70c6958d0909d9c3f9594c25a

29. Rutjes AWS, Reitsma JB, Vandenbroucke JP, Glas AS, Bossuyt PMM. Case–Control and Two-Gate Designs in Diagnostic Accuracy Studies. *Clin Chem* [Internet] 2005;51(8):1335–41. Available from: https://academic.oup.com/clinchem/article/51/8/1335/5629913

30. Mirabel M, Celermajer DS, Ferreira B, Tafflet M, Perier M-C, Karam N, et al. Screening for rheumatic heart disease: evaluation of a simplified echocardiography-based approach. *Eur Heart J Cardiovasc Imaging* [Internet] 2012;13(12):1024–9. Available from: https://academic.oup.com/ehjcimaging/article-lookup/doi/10.1093/ehjci/jes077

31. Colquhoun SM, Carapetis JR, Kado JH, Reeves BM, Remenyi B, May W, et al. Pilot study of nurse-led rheumatic heart disease echocardiography screening in Fiji--a novel approach in a resource-poor setting. *Cardiol Young* [Internet] 2013;23(4):546–52. Available from: http://www.ncbi.nlm.nih.gov/pubmed/23040535

32. Godown J, Lu JC, Beaton A, Sable C, Mirembe G, Sanya R, et al. Handheld Echocardiography Improves Detection of Rheumatic Heart Disease in Ugandan Schoolchildren Compared to Auscultation. *Circulation* [Internet] 2014;130(2).

For peer review only

Available from: http://circ.ahajournals.org/content/130/Suppl_2/A11256.short

33. Lu JC, Sable C, Ensing GJ, Webb CC, Scheel J, Aliku T, et al. Abstract 11138: Simplified Rheumatic Heart Disease Screening Criteria for Handheld Echocardiography. *Circulation* [Internet] 2014;130(Suppl 2):A11138 LP-A11138. Available from: http://circ.ahajournals.org/content/130/Suppl_2/A11138.abstract

34. Engelman D, Kado JH, Reményi B, Colquhoun SM, Carapetis JR, Donath S, et al. Focused cardiac ultrasound screening for rheumatic heart disease by briefly trained health workers: a study of diagnostic accuracy. *Lancet Glob Heal* [Internet] 2016;4(6):e386–94. Available from: http://linkinghub.elsevier.com/retrieve/pii/S2214109X16300651

35. Hardie J, Goodman N, Khan N, Barasa A, Ngunga M, Jutley R. Abstract: P1373 Feasibility of focused cardiac ultrasound by non-expert operators as a strategy for a rheumatic heart disease screening programme. *Eur Heart J* [Internet] 2016;37:241. Available from: http://doi.wiley.com/10.1111/ctr.12644

36. Lopes EL, Beaton AZ, Nascimento BR, Tompsett A, Dos Santos JP, Perlman L, et al. Telehealth solutions to enable global collaboration in rheumatic heart disease screening. *J Telemed Telecare* [Internet] 2016; Available from: http://search.ebscohost.com/login.aspx?direct=true&db=cmedm&AN=27815494&site=ehost-live

37. Diamantino A, Beaton A, Aliku T, Oliveira K, Oliveira C, Xavier L, et al. A focussed single-view hand-held echocardiography protocol for the detection of rheumatic heart disease. *Cardiol Young* [Internet] 2017;1–10. Available from:

For peer review only

https://www.cambridge.org/core/product/identifier/S1047951117001676/type/journal_article

38. Bossuyt PM, Reitsma JB, Bruns DE, Gatsonis CA, Glasziou PP, Irwig L, et al. STARD 2015: an updated list of essential items for reporting diagnostic accuracy studies. *BMJ* [Internet] 2015;277(3):h5527. Available from: http://www.bmj.com/lookup/doi/10.1136/bmj.h5527

39. Roberts K, Cannon J, Atkinson D, Brown A, Maguire G, Remenyi B, et al. Echocardiographic Screening for Rheumatic Heart Disease in Indigenous Australian Children: A Cost–Utility Analysis. *J Am Heart Assoc* [Internet] 2017;6(3):e004515. Available from: https://www.ahajournals.org/doi/10.1161/JAHA.116.004515

40. Zachariah JP, Samnaliev M. Echo-based screening of rheumatic heart disease in children: a cost-effectiveness Markov model. *J Med Econ* [Internet] 2015;18(6):410–9. Available from: http://www.tandfonline.com/doi/full/10.3111/13696998.2015.1006366

41. Musuku J, Engel ME, Musonda P, Lungu JC, Machila E, Schwaninger S, et al. Prevalence of rheumatic heart disease in Zambian school children. *BMC Cardiovasc Disord* [Internet] 2018;18(1):135. Available from: http://www.ncbi.nlm.nih.gov/pubmed/8406708

For peer review only

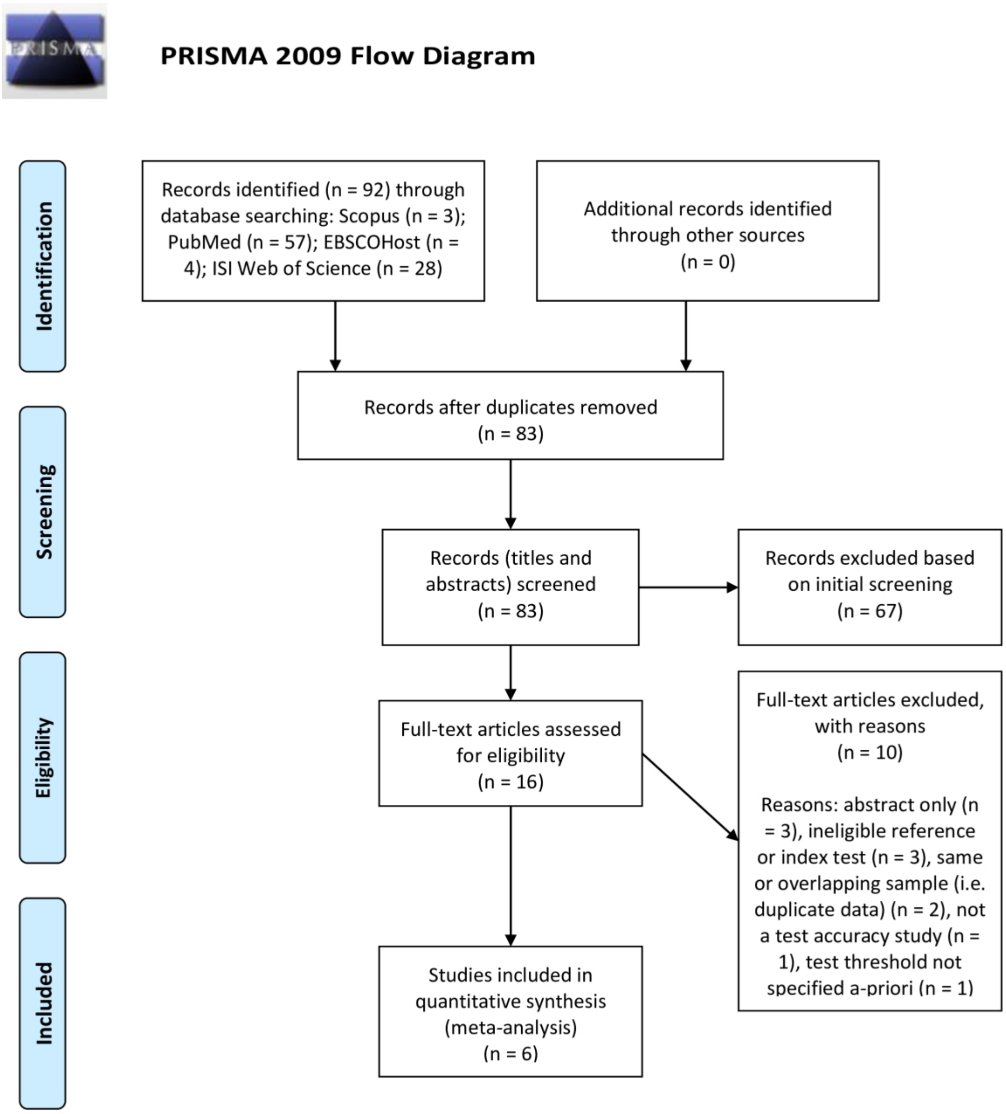

Figure 1. PRISMA flow diagram illustrating study identification, selection, eligibility and inclusion. From Moher et al.[20]

181x200mm (300 x 300 DPI)

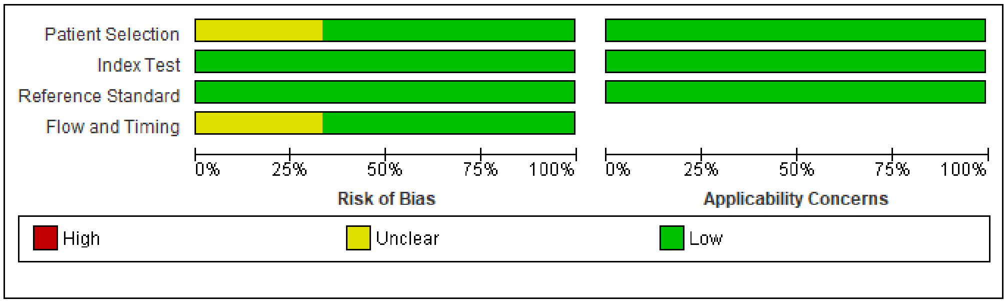

Figure 2. Risk of bias and applicability concerns graph: review authors' judgments about each domain presented as percentages across included studies.

Supplementary file 1. Search strategy.

Database	Search Terms	Limits
**PubMed**	((((((((((((((Hand-held) OR handheld) OR hand held) OR hand-carried) OR hand carried) OR HAND) OR HCU) OR HHCU) OR pocket size) OR pocket sized) OR portable) OR miniaturization) OR miniaturized) OR focused) OR focus)) AND (((("Echocardiography"[Mesh]) OR echocardiography) OR echocardiographic) OR cardiac ultrasound)) AND ((("Rheumatic Heart Disease"[Mesh]) OR rheumatic heart disease) OR RHD)<br>[MeSH terms were exploded during the search]	Limited to 2012-2020
**Scopus**	**1.** Hand-held OR handheld OR hand held OR hand-carried OR hand carried OR HAND OR HCU OR HHCU OR pocket size* OR portable OR miniatur* OR focus*<br>**2.** Echocardiograph* OR cardiac ultrasound<br>**3.** Rheumatic Heart Disease OR RHD<br>**#1 AND #2 AND #3**	Limited to 2012-2020
**ISI Web of Science**	**1.** Hand-held OR handheld OR hand held OR hand-carried OR hand carried OR HAND OR HCU OR HHCU OR pocket size OR pocket sized OR portable OR Miniaturization OR Miniaturized OR focused OR focus<br>**2.** Echocardiography OR Echocardiographic OR cardiac ultrasound<br>**3.** Rheumatic Heart Disease OR RHD<br>**Combine #1 AND #2 AND #3**	Limited to 2012–2020 filtering out MEDLINE
**EBSCO Host**	**S1.** Hand-held OR handheld OR hand held OR hand-carried OR hand carried OR HAND OR HCU OR HHCU OR pocket size OR pocket sized OR portable OR Miniaturization OR Miniaturized OR focused OR focus<br>**S2.** Echocardiography OR Echocardiographic OR cardiac ultrasound<br>**S3.** Rheumatic Heart Disease OR RHD<br>**S1 AND S2 AND S3**	Limited to 2012-2020

For peer review only - http://bmjopen.bmj.com/site/about/guidelines.xhtml

Supplementary file 2. Forest plots and Hierarchical Summary Receiver Operating Characteristic (HSROC) plots for three categorisations of RHD; any RHD (definite or borderline), definite RHD only and borderline RHD only**.**

*All plots were generated using the Review Manager (RevMan) software package, version 5.3

<u>Any RHD:</u>

Study	TP	FP	FN	TN	Expertise	Sensitivity (95% CI)	Specificity (95% CI)
Beaton (2014)	37	6	4	78	Expert	0.90 [0.77, 0.97]	0.93 [0.85, 0.97]
Beaton (2015)	141	158	39	1076	Expert	0.78 [0.72, 0.84]	0.87 [0.85, 0.89]
Beaton (2016)	44	50	9	286	Non_expert	0.83 [0.70, 0.92]	0.85 [0.81, 0.89]
Mirabel (2015)	41	106	8	1062	Non_expert	0.84 [0.70, 0.93]	0.91 [0.89, 0.93]
Ploutz (2016)	32	194	11	719	Non_expert	0.74 [0.59, 0.86]	0.79 [0.76, 0.81]
Zuhlke (2016)	21	0	5	62	Expert	0.81 [0.61, 0.93]	1.00 [0.94, 1.00]

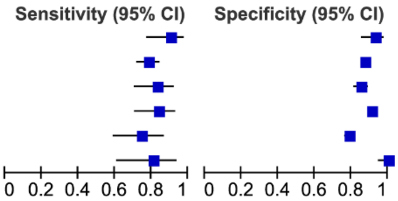

Fig 1. Forest plot of sensitivity and specificity of handheld echocardiography for any RHD

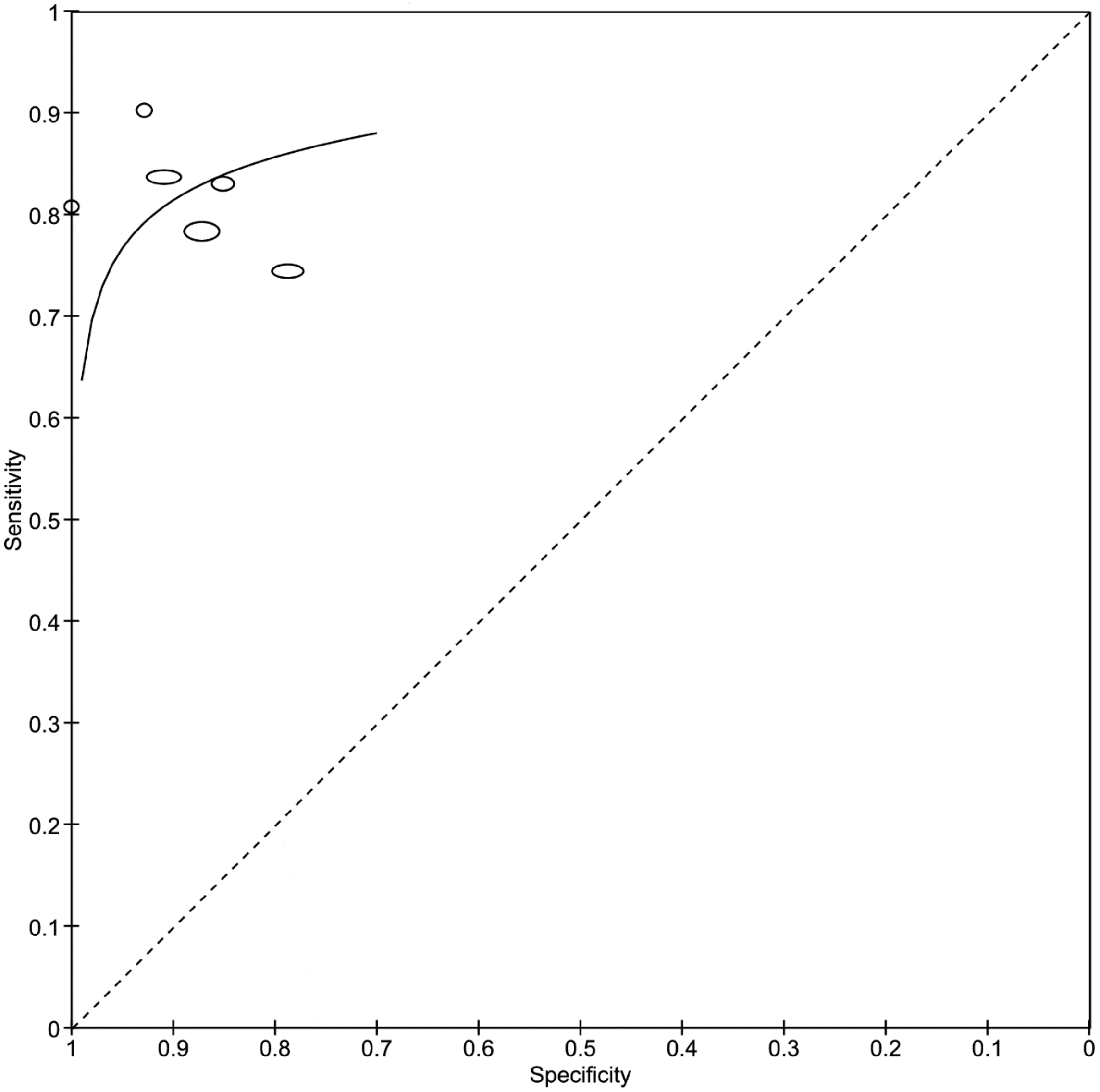

Fig 2. Summary ROC plot of sensitivity versus specificity of handheld echocardiography for any RHD

Definite RHD:

Study	TP	FP	FN	TN	Sensitivity (95% CI)	Specificity (95% CI)
Beaton (2014)	25	4	16	80	0.61 [0.45, 0.76]	0.95 [0.88, 0.99]
Beaton (2015)	46	158	1	1076	0.98 [0.89, 1.00]	0.87 [0.85, 0.89]
Mirabel (2015)	14	106	1	1062	0.93 [0.68, 1.00]	0.91 [0.89, 0.93]
Ploutz (2016)	10	194	1	719	0.91 [0.59, 1.00]	0.79 [0.76, 0.81]
Zuhlke (2016)	12	0	1	62	0.92 [0.64, 1.00]	1.00 [0.94, 1.00]

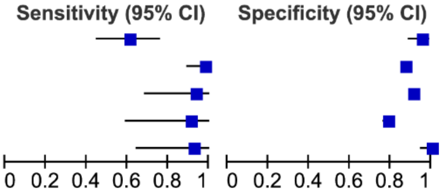

Fig 3. Forest plot of sensitivity and specificity of handheld echocardiography for definite RHD

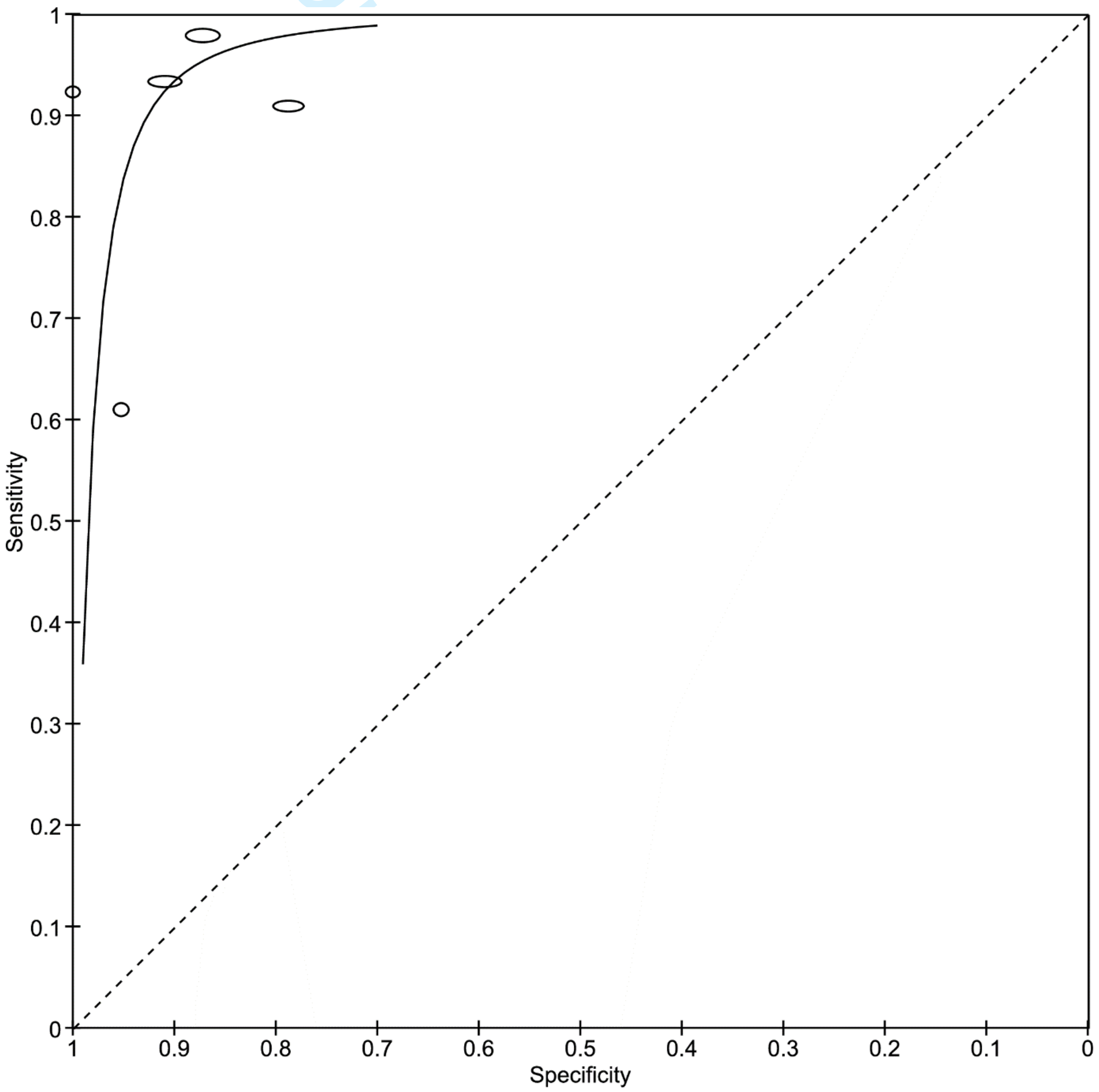

Fig 4. Summary ROC plot of sensitivity versus specificity of handheld echocardiography for definite RHD

Borderline RHD:

Study	TP	FP	FN	TN	Sensitivity (95% CI)	Specificity (95% CI)
Beaton (2014)	8	6	78	4	0.09 [0.04, 0.18]	0.40 [0.12, 0.74]
Beaton (2015)	95	158	38	1076	0.71 [0.63, 0.79]	0.87 [0.85, 0.89]
Mirabel (2015)	27	106	7	1062	0.79 [0.62, 0.91]	0.91 [0.89, 0.93]
Ploutz (2016)	22	194	10	719	0.69 [0.50, 0.84]	0.79 [0.76, 0.81]
Zuhlke (2016)	9	0	4	62	0.69 [0.39, 0.91]	1.00 [0.94, 1.00]

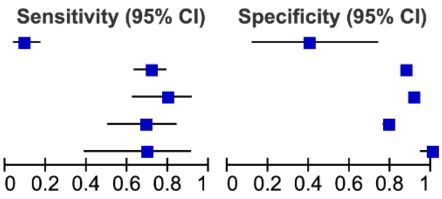

Fig 5. Forest plot of sensitivity and specificity of handheld echocardiography for borderline RHD

For

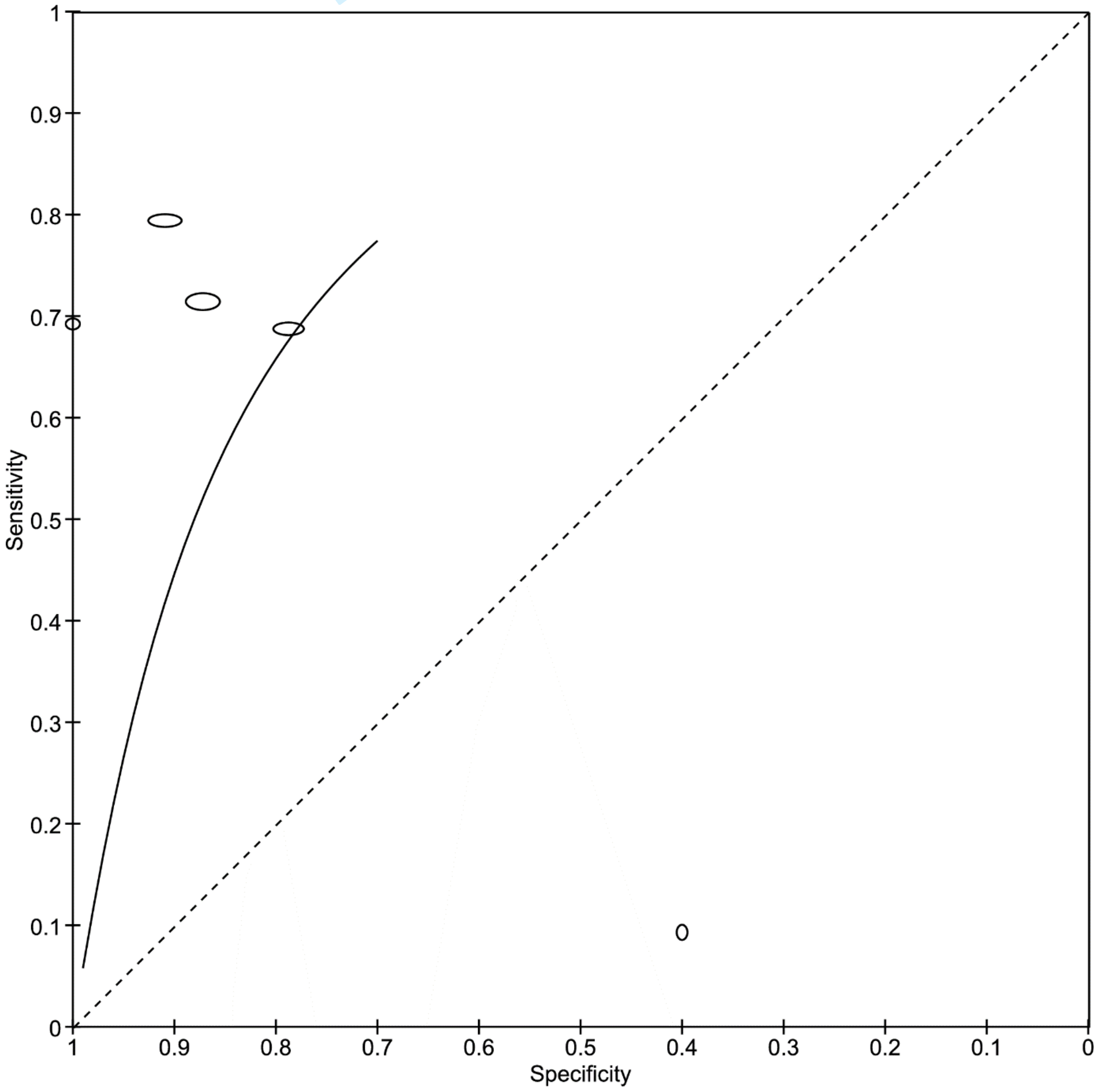

Fig 6. Summary ROC plot of sensitivity versus specificity of handheld echocardiography for borderline RHD

Supplementary file 3. HSROC plot for subgroup analysis of echocardiographer expertise.

Subgroup Analysis:

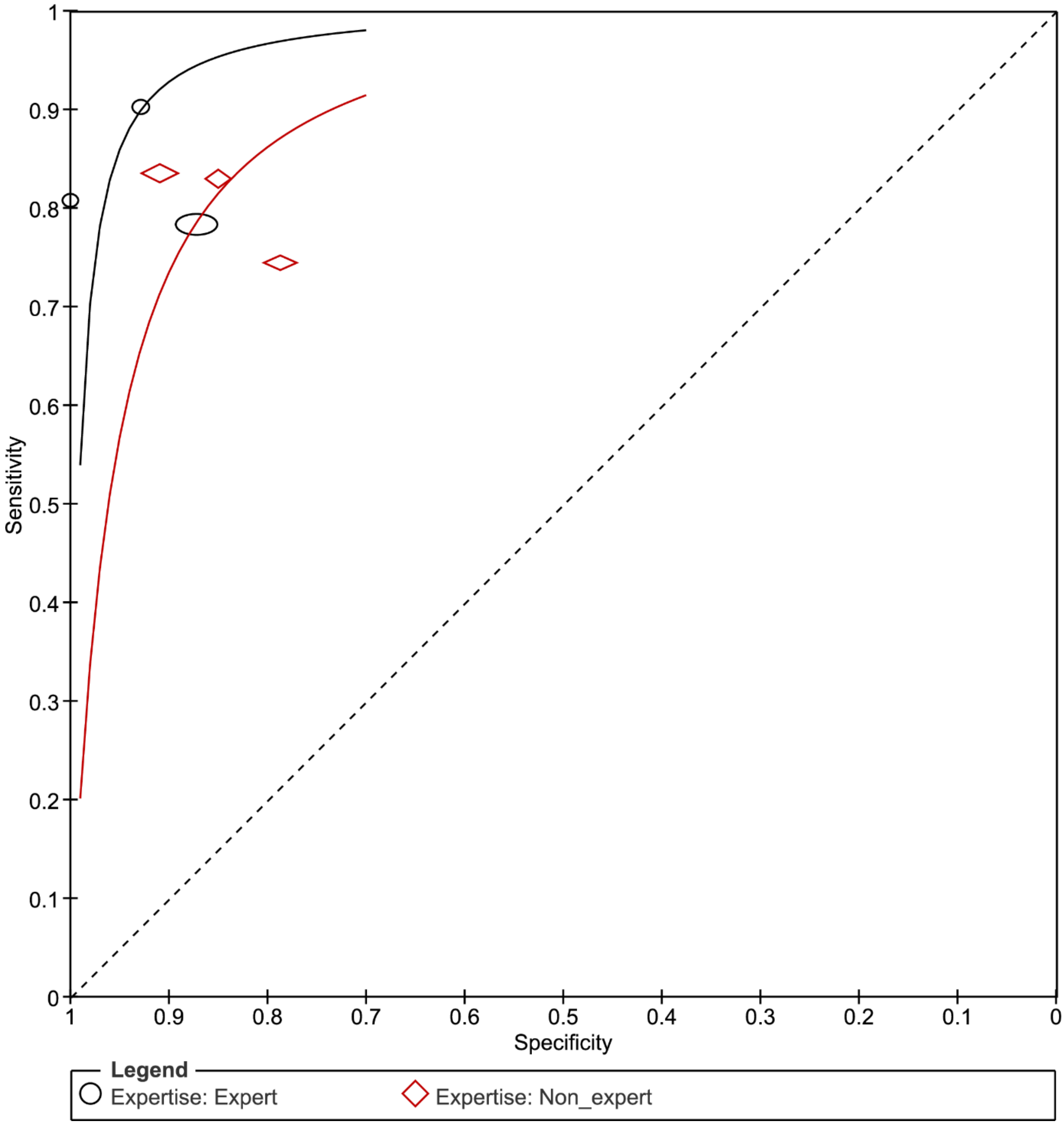

Fig 7. Summary ROC plot of sensitivity versus specificity of handheld echocardiography for any RHD according to echocardiographer expertise

PRISMA-DTA 2018 Checklist

SECTION AND TOPIC	ITEM NO.	DESCRIPTION	REPORTED ON PAGE #
**TITLE**			
Title	1	Identify the report as a systematic review (meta-analysis) of DTA studies.	1
**ABSTRACT**			
Abstract	2	Provide a structured summary including, as applicable: background; objectives; data sources; study eligibility criteria, participants, and interventions; study appraisal and synthesis methods; results; limitations; conclusions and implications of key findings; systematic review registration number.	3 - 4
**INTRODUCTION**			
Rationale	3	Describe the rationale for the review in the context of what is already known.	5 - 6
Clinical role of index test	D1	State the scientific and clinical background, including the intended use and clinical role of the index test, and if applicable, the rationale for minimally acceptable test accuracy (or minimum difference in accuracy for a comparative design).	5
Objectives	4	Provide an explicit statement of questions being addressed in terms of participants, index test, and target conditions.	5
**METHODS**			
Protocol and registration	5	Indicate where the review protocol can be accessed (e.g. web address) and provide trial registration number if available.	6
Eligibility criteria	6	Specify study characteristics (participants, setting, index test, reference standards, target conditions, and study design) and report characteristics (e.g., years considered, language, publication status) used as criteria for eligibility and providing rationale.	6 - 7
Information sources	7	Describe all information sources (e.g. databases with dates of coverage, contact with study authors to identify additional studies) in the search and the date last searched.	6, 9 & 10
Search	8	Present full search strategies for all electronic databases and other sources searched, including any limits used so that they can be repeated.	See supplementary files
Study selection	9	State the process for selecting studies (e.g., screening, eligibility, whether included in systematic review, and, if applicable, included in the meta-analysis).	6 - 8
Data collection process	10	Describe methods of data extraction from reports (e.g., piloted forms, independently, in duplicate) and any processes for obtaining and confirming data from investigators.	7
Definitions for data extraction	11	Provide definitions used in data extraction and classifications of target conditions, index tests, reference standards, and other characteristics (e.g. study design, clinical setting).	See supplementary files for editors
Risk of bias and applicability	12	Describe methods used for assessing risk of bias in individual studies and concerns regarding the applicability to the review question.	8
Diagnostic accuracy measures	13	State the principal diagnostic accuracy measures reported (e.g. sensitivity, specificity) and state the unit of assessment (e.g. per patient vs per lesion).	7

For peer review only - http://bmjopen.bmj.com/site/about/guidelines.xhtml

SECTION AND TOPIC	ITEM NO.	DESCRIPTION	REPORTED ON PAGE #
Synthesis of results	14	Describe the methods of handling the data, combining the results of the studies and describing the variability between studies. This could include, but is not limited to (1) handling of multiple definitions of the target condition, (2) handling of multiple thresholds of test positivity, (3) handling multiple index test readers, (4) handling of indeterminate test results, (5) grouping and comparing tests, and (6) handling of different reference standards.	8 - 9
Meta-analysis	D2	Report statistical methods used for meta-analyses if performed.	8 - 9
Additional analyses	16	Describe the methods of the additional analyses (e.g. sensitivity or subgroup analyses, meta-regression), if done, indicating which were prespecified.	8 - 9
**RESULTS**			
Study selection	17	Provide the numbers of studies screened, assessed for eligibility, included in the review, and included in the meta-analysis if applicable, with reasons for exclusions at each stage, ideally with a flow diagram.	10 - 11
Study characteristics	18	For each included study, provide citations and present key characteristics including (1) participant characteristics (presentation, prior testing), (2) clinical setting, (3) study design, (4) target condition definition, (5) index test, (6) reference standard, (7) sample size, and (8) funding sources.	10 - 12
Risk of bias and applicability	19	Present evaluation of risk of bias and concerns regarding applicability for each study.	13
Results of individual studies	20	For each analysis in each study (e.g. unique combination of index test, reference standard, and positivity threshold), report 2 × 2 data (TP, FP, FN, TN) with estimates of diagnostic accuracy and confidence intervals, ideally with a forest plot or a receiver operating characteristic curve.	See Supplementary files
Synthesis of results	21	Describe test accuracy, including variability; if meta-analysis was done, include results and confidence intervals.	14 - 15
Additional analysis	23	Give results of additional analyses, if done (e.g. sensitivity or subgroup analyses, meta-regression, analysis of index test, failure rates, proportion of inconclusive results, and adverse events).	15 - 16
**DISCUSSION**			
Summary	24	Summarize the main findings including the strength of evidence.	17 - 19
Limitations	25	Discuss limitations from included studies (e.g. risk of bias and concerns regarding applicability) and from the review process (e.g., incomplete retrieval of identified research).	19 - 20
Conclusions	26	Provide a general interpretation of the results in the context of other evidence. Discuss implications for future research and clinical practice (e.g. the intended use and clinical role of the index test).	20 - 22
**FUNDING**			
Funding	27	For the systematic review, describe the sources of funding and other support and the role of the funders.	See funding statement

Abbreviations: FN, false negative; FP, false positive; TN, true negative; TP, true positive

From: McInnes MDF, Moher D, Thombs BD, McGrath TA, Bossuyt PM; and the PRISMA-DTA Group (2018). Preferred Reporting Items for a Systematic Review and Meta-analysis of Diagnostic Test Accuracy Studies: The PRISMA-DTA Statement. JAMA 319(4): 388 - 396. doi: 10.1001/jama.2017.19163.

For peer review only

The PRISMA for Abstracts Checklist

TITLE	CHECKLIST ITEM	REPORTED ON PAGE #
1. Title:	Identify the report as a systematic review, meta-analysis, or both.	1 and 3
**BACKGROUND**		
2. Objectives:	The research question including components such as participants, interventions, comparators, and outcomes.	3
**METHODS**		
3. Eligibility criteria:	Study and report characteristics used as criteria for inclusion.	3
4. Information sources:	Key databases searched and search dates.	3
5. Risk of bias:	Methods of assessing risk of bias.	3
**RESULTS**		
6. Included studies:	Number and type of included studies and participants and relevant characteristics of studies.	3
7. Synthesis of results:	Results for main outcomes (benefits and harms), preferably indicating the number of studies and participants for each. If meta-analysis was done, include summary measures and confidence intervals.	3-4
8. Description of the effect:	Direction of the effect (i.e. which group is favoured) and size of the effect in terms meaningful to clinicians and patients.	N/A
**DISCUSSION**		
9. Strengths and Limitations of evidence:	Brief summary of strengths and limitations of evidence (e.g. inconsistency, imprecision, indirectness, or risk of bias, other supporting or conflicting evidence)	4
10. Interpretation:	General interpretation of the results and important implications	4
**OTHER**		
11. Funding:	Primary source of funding for the review.	N/A
12. Registration:	Registration number and registry name.	4
